# Consensus statement for managing patients with ARDS in low-resource settings: a Delphi study

**DOI:** 10.1016/j.aicoj.2026.100144

**Published:** 2026-08-27

**Authors:** Pedja Kovacevic, Jesús Villar, Boris Tomic, Fabio Silvio Taccone, Giacomo Grasselli, Luigi Camporota, Marlies Ostermann, Ivan Palibrk, Rajesh Mishra, Sasa Dragic, Jose Diaz-Gomez, Danica Momcicevic, Ali Ait Hssain, Biljana Zlojutro, Jihad Mallat, Milka Jandric, Owoniya Temitope, Roman Zahorec, Omar Shahed, Mohamed Boussarsar, F. Joachim Meyer, Ahmed Mukhtar, Marko Kutlesa, Vladimir Krajinovic, John Laffey, Radmilo Jankovic, Samir Jaber, Dejan Markovic, Oluwayemisi Esther Ekor, Rotimi Muyiwa, Rihard Knafelj, Matija Jurjevic, Natasa Milivojevic, Ahmed El Masry, Jasminka Persec, Anusha Cherian, Younes Aissaoui, Oguz Kilickaya, Kent Doi, Daniel Lovric, Wael Alqassem, Natasa Gocic, Aderonke Adesiyan, Reuben Okioma, Ayalew Zewdie, Mumbere Kigayi Jean Pierre, Subhash Acharya, Ognjen Gajic

**Affiliations:** a University Clinical Centre of the Republic of Srpska, Medical Intensive Care Unit, Banja Luka, the Republic of Srpska, Bosnia and Herzegovina; b Faculty of Medicine, University of Banja Luka, Banja Luka, the Republic of Srpska, Bosnia and Herzegovina; c Research Unit at Hospital Universitario Dr. Negrín, Fundación Canaria Instituto de Investigación Sanitaria de Canarias, Las Palmas, Spain, CIBER de Enfermedades Respiratorias, Instituto de Salud Carlos III, Madrid, Spain, Faculty of Health Sciences, Universidad del Atlántico Medio, Tafira, Las Palmas. Spain, Li Ka Shing Knowledge Institute, St. Michael’s Hospital, Toronto, Canada; d Department of Intensive Care, Hôpital Universitaire de Bruxelles (HUB), Université Libre de Bruxelles (ULB), Brussels, Belgium; e Department of Emergency, Fondazione IRCCS Ca’ Granda Ospedale Maggiore Policlinico, Milan, Italy, Department of Pathophysiology and Transplantation, University of Milan, Milan, Italy; f Department of Adult Critical Care, Guy's and St Thomas' NHS Foundation Trust, London, SE1 7EH, United Kingdom; g Department of Critical Care, King's College London, Guy's and St Thomas' NHS Foundation Trust, London, United Kingdom; h Department of Anesthesiology, Reanimatology and Intensive Care, Clinic for Abdominal Surgery, University Clinical Centre of Serbia, 11000 Belgrade, Serbia; i Critical Care, Shaibya Comprehensive Care Clinic, ESICM National Representative of India, Ahmedabad, Gujarat, India; j Integrated Hospital Care Institute (Divisions of Anesthesiology, Critical Care Medicine, Emergency Medicine, Hospital Medicine, Infectious Disease, and Pulmonary Medicine), Cleveland Clinic Abu Dhabi, Abu Dhabi, United Arab Emirates; k Medical Intensive Care Unit, Hamad General Hospital, Doha, Qatar, Department of Medicine, Weill Cornell Medical College, Doha, Qatar; l Critical Care Division, Integrated Hospital Care Institute, Cleveland Clinic Abu Dhabi, 112412, Abu Dhabi, United Arab Emirates and Cleveland Clinic Lerner College of Medicine of Case Western Reserve University, Cleveland, OH, 44106, United States of America; m Department of Anaesthesia and Intensive Care Unit, Obafemi Awolowo University Teaching Hospital Complex (OAUTHC), Ile-Ife, Nigeria; n Department of Anesthesiology and Intensive Medicine, Medical School, Comenius University, Heydukova 10, 812 50 Bratislava, Slovakia; o Division of Critical Care, University of Witwatersrand, Chris Hani Baragwanath Academic Hospital, Johannesburg, South Africa; p Medical Intensive Care Unit, Research Laboratory LR12SP09 "Heart Failure", Farhat Hached University Hospital, Sousse, Tunisia; q München Klinik gGmbH and Medical Faculty, Lung Center Munich, University of Heidelberg, Heidelberg, Germany; r Department of Anesthesia and Critical Care, Cairo University, Cairo, Egypt; s Department for Intensive Care, University Hospital for Infectious Diseases "Dr. Fran Mihaljević", 10000 Zagreb, Croatia; t Anaesthesia and Intensive Care Medicine, School of Medicine, Clinical Sciences Institute, Galway University Hospitals, University of Galway, Galway, Ireland; u University Clinical Center Niš, Department of Anesthesia and Intensive Therapy, Faculty of Medicine, University of Niš, Niš, Serbia; v Department of Anesthesia and Intensive Care Unit, Regional University Hospital of Montpellier, St-Eloi Hospital, University of Montpellier, PhyMedExp, INSERM U1046, CNRS UMR, Montpellier, France; w Department of Anesthesiology, Reanimatology and Intensive Care, Clinic for Cardiac Surgery, University Clinical Centre of Serbia, 11000 Belgrade, Serbia; x University of Cape Coast Ghana, Ghana; y Department of Anaesthesia, Lagos University Teaching Hospital, Idi-Araba, Lagos State, Nigeria; z Center for Internal Intensive Medicine, University Medical Center Ljubljana, Ljubljana, Slovenia; A Clinic of Anesthesiology, Resuscitation and Intensive Care, General Hospital Josip Bencevic, Slavonski Brod, Croatia; B Neurological Intensive Care Unit, University Clinical Centre of Ljubljana, Ljubljana, Slovenia; C Al Adan Hospital, Al Ahmadi, Kuwait; D Clinical Department for Anesthesiology, Reanimatology and Intensive Care Medicine, University Hospital Dubrava, University of Zagreb School of Dental Medicine, 10000 Zagreb, Croatia; E Department of Anesthesiology and Critical Care, Jawaharlal Institute of Postgraduate Medical Education and Research, Puducherry, India; F Department of Critical Care Medicine, Avicenna Military Hospital, Marrakesh, Morocco; G Department of Medicine, Division of Pulmonary and Critical Care Medicine, Mayo Clinic, Rochester, MN, United States of America; H Department of Emergency and Critical Care Medicine, The University of Tokyo, Tokyo, Japan; I Cardiology Clinic, University Hospital Center Zagreb, 10000 Zagreb, Croatia; J Critical Care Department, Prince Mohammed Bin Abdulaziz Hospital, Riyadh, Saudi Arabia; K University Clinical Centre of Vojvodina, Emergency Center, Novi Sad, Serbia; L Africa Health Science University, Rwanda; M Department of Critical Care, The Nairobi Hospital, Nairobi, Kenya; N St Paul's Hospital Millenium Medical College, Addis Ababa, Ethiopia; O Department of Anesthesiology and Intensive Care, HEAL Africa Tertiary Hospital, Goma, North Kivu, Congo; P Department of Critical Care Medicine, MMC, Institute of Medicine, Tribhuvan University, Kathmandu, Nepal

**Keywords:** Acute respiratory distress syndrome, Low resource settings, Mechanical ventilation, Non-invasive ventilation, Therapeutic management

## Abstract

**Background:**

Despite decades of research and advances in critical care, mortality of the Acute Respiratory Distress Syndrome (ARDS) remains high, ranging from 30 to 45% in high-income countries and markedly higher in low-resource settings (LRS), where management guidelines implementation is constrained. An international consensus aimed to provide statements for ARDS management tailored to LRS.

**Methods:**

Since management of critically ill patients is a major public health problem in LRS, the Bosnia and Herzegovina Society of Intensive Care Medicine convened an international, multidisciplinary Steering Committee with expertise in ARDS management. The committee appointed an international panel of clinicians and investigators specialized in the field of ARDS. Through an iterative Delphi process, a panel of 52 experts developed several consensus statements.

**Results:**

After three Delphi rounds, consensus was reached on 44 out of 56 of statements (79%). The survey covered eight domains related to ARDS management in LRS related to: critical care infrastructure, diagnostic tools, respiratory support, other supportive therapies, sedation strategies, weaning, nutrition, and management of complications. Statements were compared with recent ESICM guidelines, highlighting specific LRS considerations.

**Conclusion:**

Using the Delphi method, international experts developed clinical practice statements guiding clinicians on ARDS management in LRS. These statements complemented existing guidelines by addressing areas where evidence is limited, and highlight aspects of ARDS management pertinent for LRS not covered by current international recommendations. Future studies are needed to evaluate the adherence and impact of these statements and to address remaining uncertainties.

## Graphical abstract

**Figure:**
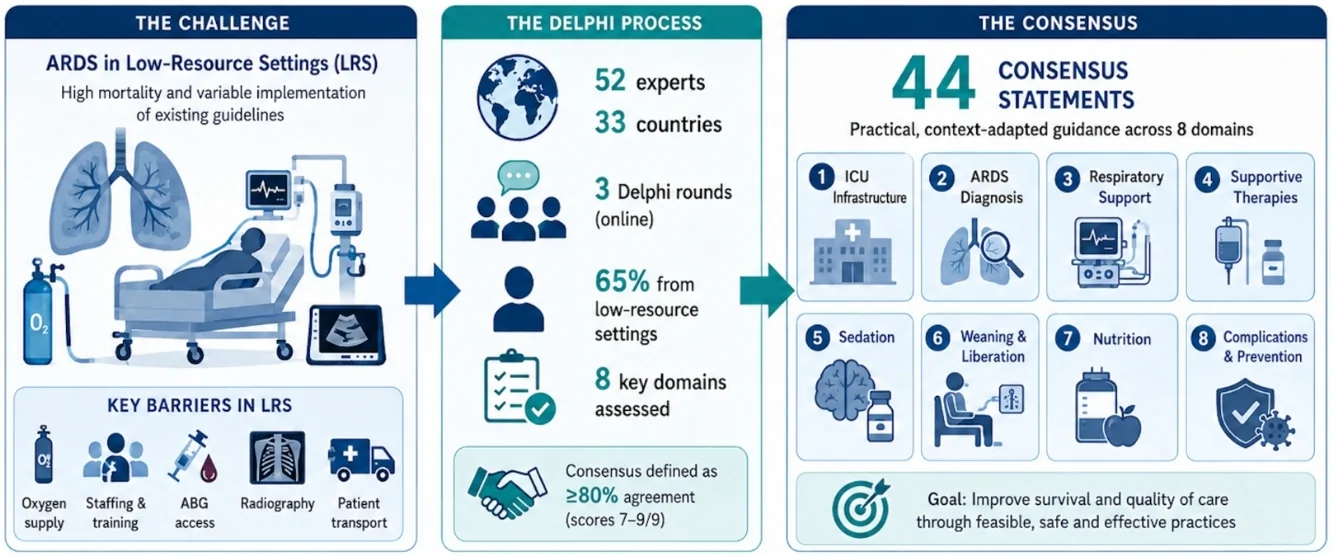

## Introduction

Despite decades of research and advances in supportive critical care, the mortality of patients with the Acute Respiratory Distress Syndrome (ARDS) has remained largely unchanged and unacceptably high [1]. ARDS is a pulmonary inflammatory condition due to multiple pulmonary and systemic processes sharing common clinical and pathological features [2]. Prior to the COVID-19 pandemic, average ARDS mortality varied widely from 30% to 45%, where randomized controlled trials (RCTs) reported lower mortality compared to observational studies [3].

Although these figures primarily originate from developed healthcare systems, largely in high-income countries (HICs), reported ARDS mortality in low-resource settings (LRS) remains higher because relevant infrastructure and human resources are not routinely available [4,5]. In addition, diagnosing ARDS in some LRS remains challenging, as the requirements of conventional diagnostic criteria are frequently difficult to meet because of limited access to arterial blood gas analysis, chest radiography, and intensive care resources [6].

**Fig. 1:**
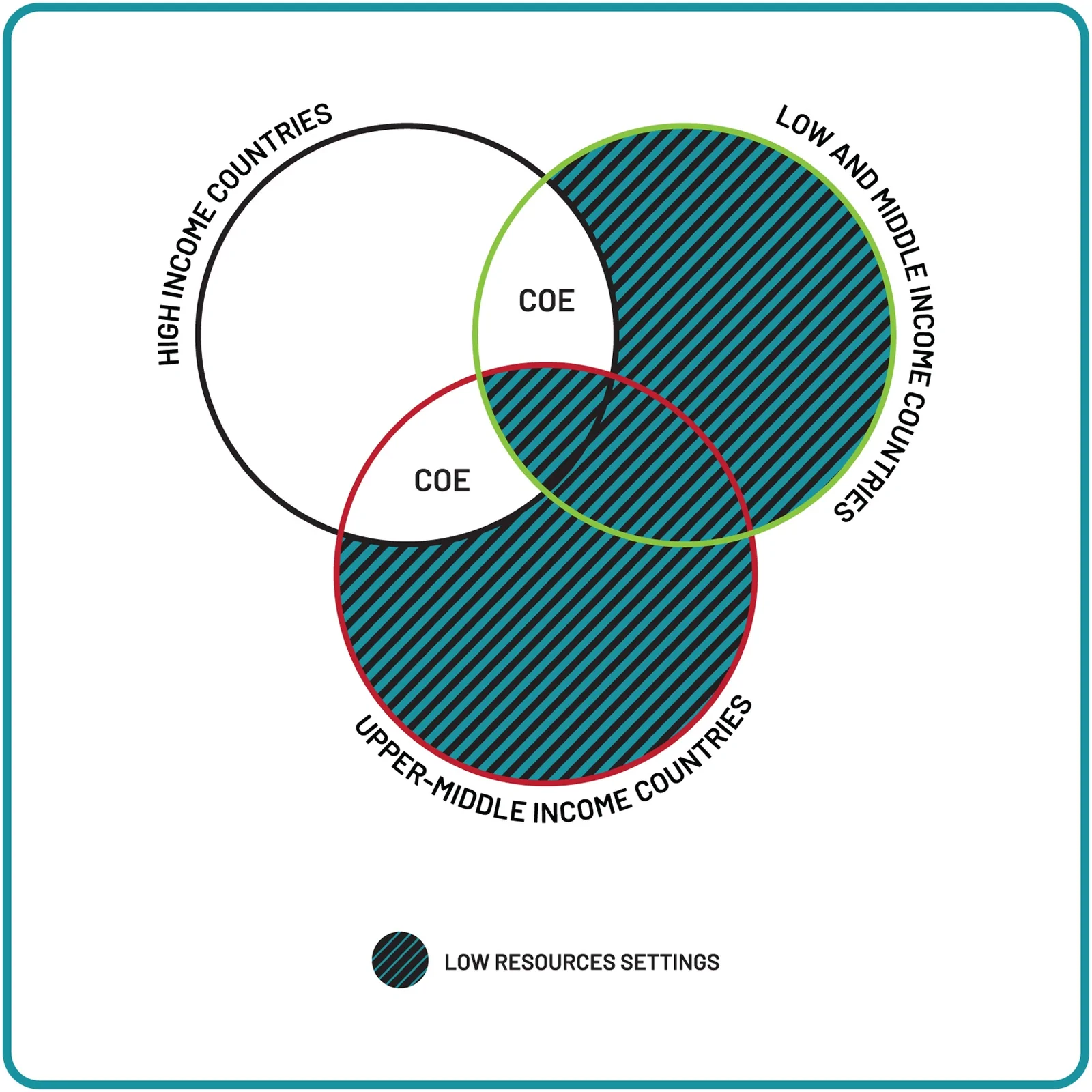
Stylized representation of the relationship between low-resource settings (LRS) and World Bank country income classifications. The figure illustrates the potential mismatch between economic transition and the development of critical care systems. While countries may rapidly progress from low-income to middle-income or upper-middle-income status, the evolution of critical care infrastructure, workforce capacity, education, and service delivery may occur at a much slower pace. Conversely, well-resourced centers of excellence (COEs) may exist within otherwise resource-constrained environments, whereas low-resource settings may also persist within high-income countries.

Notably, while LRS most commonly refers to low- and middle-income countries (LMICs), some high-income countries (HICs) may also contain low-resource settings, whereas well-resourced ICUs can exist in LMICs, albeit relatively infrequently (Fig. 1) [7]. A substantial proportion of the global burden of ARDS in LRS occurs in middle-income countries (MICs). Although low-income countries (LICs) face profound resource constraints, reported ARDS mortality rates are often similar across LICs, MICs, and even some HICs [[7], [8], [9], [10]].

Conversely, many countries rapidly transition from low-income to middle-income or even upper-middle-income status according to World Bank classifications, as observed in Bosnia and Herzegovina and several countries of the former Yugoslavia and Eastern Europe. However, the development of critical care systems rarely progresses at the same pace. This mismatch between economic and healthcare transition may obscure persistent deficiencies in critical care capacity and contribute to preventable mortality. Importantly, a substantial proportion of the world’s population lives in such transitional settings, underscoring the global relevance of this issue [[7], [8], [9], [10], [11]].

Although there are established clinical guidelines and recommendations for the management of ARDS patients, their widespread implementation is limited to developed systems [12]. Several studies have shown that these guidelines are primarily based on evidence generated in affluent, high-resource ICUs within HICs, which are likely unfeasible to apply in LRS [[13], [14], [15]]. The recent COVID-19 pandemic exposed the limitations and challenges in the definition, diagnosis, and management of ARDS patients in LRS [16,17]. Although sites with limited resources could represent a reason for low adherence to existing guidelines, some standard management techniques, such as prone positioning and neuromuscular blockade, are less frequently applied in LRS hospitals despite being low-cost techniques, due to the lack of trained staff with experience [18]. In addition, one of the biggest challenges in those countries is the lack of research, specifically into diagnosis, treatment, and outcome of ARDS patients [19,20].

Given the paucity of context-specific data, we applied a Delphi method to systematically capture expert consensus across diverse healthcare systems. The main objective was to provide recommendations on management strategies for ARDS patients in LRS, where there are some barriers for implementation of existing clinical guidelines (e.g., oxygen supply, staffing ratios, lack of arterial blood gas [ABG] analyzers, limited radiography, and transport constraints), or where specific guidance addressing ARDS management challenges relevant to these settings are lacking.

## Research in context

### Evidence before this study

On January 1, 2025, we started a search in PubMed, Embase, Web of Science, and Google Scholar for studies published in English from Jan 1, 2010, to June 30, 2025, using the terms “acute respiratory distress syndrome” OR “ARDS” AND (“low-resource settings” OR “resource-limited settings” OR “low- and middle-income countries”) AND (“management” OR “guidelines” OR “critical care” OR “Delphi”). Evidence from HICs consistently shows that interventions such as low-tidal volume ventilation, prone positioning, neuromuscular blockade, and conservative fluid strategies improve outcomes in well-resourced ICUs. However, most ARDS research originated from health systems with advanced diagnostics, reliable oxygen delivery, and adequate staffing. Studies from LRS reported considerably higher mortality and important barriers for applying international guidelines, including oxygen shortages, limited monitoring, insufficient trained personnel, and infrastructure constraints. No prior study has systematically developed consensus-based ARDS management statements tailored to these settings. Additionally, expert-driven guidance on diagnostic tools, sedation, liberation from mechanical ventilation, nutrition, and complication prevention in LRS is largely absent. This Delphi exercise has generated a global multidisciplinary consensus, specifically focused on ARDS management in LRS.

### Added value of this study

This international Delphi project represents the first global effort to develop expert clinical practice statements tailored to ARDS management in LRS. Input from 52 experts across 33 countries, 65% from LRS, identified eight domains in which existing ARDS recommendations do not adequately address LRS realities. These recommendations include critical care infrastructure, feasible diagnostic approaches when arterial blood gases or advanced imaging are unavailable, context-appropriate respiratory support strategies, structured sedation and weaning protocols, nutritional practices, and prevention of complications. Our findings highlight areas where current guidelines do not align with LRS needs, but also domains requiring alternative or modified recommendations. This study fills a major evidence gap by providing structured, consensus-based guidance for ARDS management in environments where international guidelines are often difficult or impossible to implement.

### Implications of all the available evidence

Current ARDS guidelines were developed for developed systems and do not address key limitations in LRS. The consensus statements from this study offer recommendations for diagnosing and managing ARDS in LRS. They emphasize critical care infrastructure strengthening, optimization of oxygen delivery, use of simple diagnostic tools such as SpO₂/FiO₂ and lung ultrasound, safe and practical respiratory support approaches, structured sedation, weaning and nutritional strategies. These statements provide a framework for improving ARDS outcomes and guiding training, capacity building, and resource allocation in LRS. Adoption of these recommendations may help reduce global disparities in reported ARDS mortality. Future research should assess feasibility, adherence, and clinical impact in diverse LRS contexts.

## Methods

### Study design - Delphi process

An international multidisciplinary Steering Committee (OG, PK, BT, FST, JV, GG, LC, MO, JD, and OT), composed of members from various geographic regions and both developed systems and LRS, who were experts in managing ARDS patients, including Delphi methodologist (BT) was convened by the Association of Intensivists of Bosnia and Herzegovina Medical Intensive Care Society. The modified Delphi consensus methodology is well described, widely used, and relies on expert opinion to address questions where empirical data are unavailable, insufficient, or not directly applicable. The Delphi process is an interactive survey method in which relevant stakeholders (experts) receive a series of questionnaires (termed rounds) and are asked to rate the importance (a process called assessment weighting, where a numerical value or percentage is assigned to different parts of an evaluation to reflect their differing levels of significance by the members of the expert panel) of each proposed and identified outcome for inclusion in the consensus. This process generally involves at least two rounds of voting on recommendations related to a specific study question (*see Supplemental File*). Voting is guided by results from previous rounds and conducted anonymously to reduce external influence [21]. After reviewing the literature, the Surveylet software (Calibrum Inc., Saint George, Utah, U.S.A.) was selected [22]. This software was used to generate expert consensus and draft statements on the management of ARDS in LRS. For this survey, “LRS” refers to contexts with limited availability of trained healthcare professionals, infrastructure, laboratory diagnostics, and equipment needed to manage ARDS. Panellists characterized their practice environments after reviewing the operational description of LRS provided on the Delphi platform. Classification was therefore based on the resource characteristics of their clinical setting rather than solely on the World Bank income classification of the country of affiliation. Healthcare in LRS differs from that in developed systems not only because of resource constraints, but also due to differences in disease epidemiology, socio-economic context, access to care, and approaches to ARDS recognition and management. The diagnosis of patients with ARDS was defined according to Berlin criteria [23]. Survey rounds were conducted based on previously published Delphi survey studies and reported according to the Accurate Consensus Reporting Document (ACCORD) guidelines [24]. This Delphi study was not prospectively registered. The ACCORD Checklist is provided in the *Supplemental File*.

### Study participants (panelists)

A multidisciplinary and international Delphi panel was assembled using predefined, expertise-based selection criteria. Participants were selected based on demonstrated clinical experience in adult ARDS management and documented involvement in ARDS-related research, education, policy or protocol development, or other relevant professional activities. For the purposes of this study, “expertise” was operationally defined using objective indicators, including relevant research output, academic and/or clinical leadership roles, and professional experience in the field of ARDS. Participants meeting these criteria were considered experts within the context of the Delphi process. Potential panelists were nominated through professional networks and by national and international coordinators of the European Society of Intensive Care Medicine (ESICM), based on predefined objective criteria, including demonstrated clinical experience in ARDS management, academic contributions, and involvement in guideline development or research relevant to the study questions. A deliberate effort was made to ensure broad geographical representation and inclusion of experts from diverse healthcare systems, particularly those from or with substantial professional experience in LRS. Most selected panelists were either from, or had prior professional experience working in, low-resource settings, and/or had previous experience participating in Delphi consensus processes. Initial contact was made via email, providing a brief overview of the study.

### Consensus process

A total of 55 potential experts in the field were invited by email to participate in the Delphi study. Among them, 52 accepted the invitation and were subsequently enrolled in the Delphi panel. Accordingly, response rates, as well as related descriptive statistics, were calculated using 52 as the denominator. The survey questionnaire was distributed to the panel via email. The identities of experts were kept confidential until completion of the Delphi process. Each panelist was asked to rate the importance of the items using a 9-point Likert scale. Evaluation of this scale, which is commonly divided into three categories for core outcome set projects, was as follows: Not Important (1–3), Important but Not Critical (4–6), and Critical (7–9). Also, they were invited to provide additional comments if desired. All participants could re-access the online platform asynchronously to revise their responses during the survey period, which was a specific timeframe from May 15 to August 8, 2025. Panel members could only view their own responses and could not see the responses from other participants.

We predefined consensus as ≥80% of panel ratings between 7 and 9 on a 9-point Likert scale, and strong consensus as ≥90%. Items with <50% agreement were categorized as dissent and discharged. Items with 50–79% agreement were revised using panel comments and resubmitted. The Steering Committee reviewed all free-text comments, including those on statements reaching consensus in Round 1, to determine whether revision was warranted; all comments were retained verbatim in the Supplementary Material for transparency. Stability was defined a priori as (a) median change <1 point between rounds and (b) absolute change in percent ratings (7–9 < 10 percentage points); when both were met, and consensus was achieved, items were closed.

The scope of the project was developed through a qualitative evidence synthesis based on a focused review of the published literature on ARDS management in LRS, together with the published ESICM ARDS guidelines [13]. These guidelines were selected because they represented the first recently published comprehensive European ARDS guideline and provided a practical framework for identifying implementation barriers relevant to LRS. Given that this Delphi initiative was developed in Europe, they served as a natural reference framework for survey development. The literature review revealed a lack of high-quality evidence for managing ARDS in LRS, and the project's goal was to suggest alternative solutions where barriers to evidence-based practice exist. The domains were defined after detailed discussions among the Steering Committee members, following identification of barriers for implementing current international ARDS management guidelines and gaps in the literature related to ARDS in LRS. Although some domains were not exclusive to ARDS, they were intentionally included because infrastructure, monitoring, sedation, infection prevention and nutrition directly influence the feasibility, delivery, and effectiveness of ARDS management in LRS. The initial Delphi survey was internally validated by the research team and underwent pilot testing within the Calibrum platform before distribution to the expert panel. Feedback obtained during pilot testing and subsequent Delphi rounds was used to refine statement wording and improve clarity. No face-to-face meetings were held during the process. The literature search strategy and the corresponding PRISMA flow diagram outlining the studies included in the qualitative evidence synthesis are provided in the *Supplemental File*.

### Ethical consideration

The Ethics Committee of the University Clinical Centre of the Republic of Srpska deemed the study exempt from formal ethical approval and informed consent since it involved no-risk on patients. Panel members gave consent by survey participation. All steering committee members report no conflicts of interest related to industry or medical device manufacturers.

### Data Analysis (consensus and stability)

Quantitative data collected through the Delphi process were analyzed using descriptive statistics. Consensus among experts was defined *a priori* as an agreement reaching a predetermined threshold (e.g., ≥80% agreement).

Consensus for each statement was assessed using the median rating, as a robust measure of central tendency appropriate for ordinal Likert-scale data. The stability and dispersion of panel responses were evaluated using the interquartile range (IQR) and the coefficient of variation (CoV). The IQR was reported as Q1–Q3 and used to describe the extent of convergence of responses around the median. The CoV was calculated as the standard deviation divided by the mean rating, with lower values indicating less relative variability and greater response stability. Together, these measures provided complementary information: the median reflected the overall level of agreement, whereas the IQR and CoV characterized the degree of dispersion, convergence, and stability of ratings across Delphi rounds. Stability was assessed independently of consensus; therefore, persistent stable disagreement was considered a meaningful finding that could reflect genuine differences in resource availability and clinical practice across heterogeneous LRS.

After each Delphi round, the panelists were informed of the results through a consensus report, as well as of the measure of consensus stability for each individual statement.

Qualitative data, including expert comments and suggestions, were analyzed thematically to identify recurring themes and patterns. Data from the last stable questionnaire round for each statement were included for preparing the final statements or recommendations. The Steering Committee developed expert clinical practice statements based on the items that reached consensus and response stability through the Delphi process. The final findings, including the agreed-upon statements and the draft manuscript, were shared with all participating experts for review, feedback, and approval before submission for publication. Additionally, research priorities in this area were identified by analyzing expert feedback collected throughout the Delphi rounds, as well as by addressing gaps highlighted in the existing literature.

## Results

From 55 experts invited to participate, 52 responded in the first round (response rate: 92.3%), and 49 subsequently agreed to participate and completed rounds two and three (response rate: 89.1%). Participating experts represented 33 countries across five continents, with more than two-thirds (65.39%) from LRS, including three (30%) of the ten members of the Steering Committee. The largest proportion of participating experts (48%) had between 40 and 49 years of age, and 12 (22%) participants were female. Most experts were employed at university-affiliated hospitals (76%) or public hospitals (7%), with 17% working in private facilities. Demographic profile of individual experts, including their primary specialty and critical care training, is provided in ***eTables*** and ***eFigures*** (*Supplemental File)*.

Three Delphi rounds were conducted between May 15 and August 9, 2025, with 44 (79%) of the statements and questions achieving consensus and stability. In the first domain, ***Critical Care Infrastructure Requirements for ARDS Management in Low-Resource Settings***, consensus was achieved on all nine statements; five reached consensus in the first round, and after a second round of voting, consensus was reached on the remaining four statements (Table 1). The second domain, ***Diagnostic tools for the identification of ARDS in LRS***, included four statements. Consensus was reached on one statement during the first round, while there was agreement on the other three in the second round (Table 1). ***Treatment-respiratory support*** for patients with ARDS in LRS represented the third domain. Seventeen statements were voted (Table 2); the last was not directly related to treatment but to ARDS definition. Consensus was reached in the first round for five statements, in the second round for 10 statements, while consensus was not achieved for the remaining two statements after the third round. Regarding ***other supportive therapies*** of domain 4 with 11 statements, only four statements achieved consensus after three rounds of voting (Table 3). ***Sedation strategies***, representing domain 5, included three statements. All reached consensus within the first two rounds (Table 3). ***Weaning*** was domain 6, where three of four proposed statements reached consensus after three rounds of voting (Table 3). ***Nutrition and management of complications*** fell under domains 7 and 8. In domain 7, consensus was reached on four statements after three voting rounds. In domain 8, consensus was achieved on two of the three statements.

**Table 1:** List of statements/recommendations for the first and second domains that reached consensus across three Delphi rounds.

	Statements/Recommendations	Stability	Consensus status	IQR	Median	CoV
N^o^	First domain: *Critical Care Infrastructure Requirements for ARDS Management in Low-Resource Settings (LRS)*					
1.	Initial management, resuscitation and stabilization of ARDS patients (first 24–48 hours) should take place in healthcare facilities offering oxygen therapy and at least one non-invasive respiratory support method, such as a level I intensive care unit (ICU) or in a “shock room”.	81,1%	Consensus in the first round	1	8	18.93%
2.	Patients with ARDS—primarily those with mild forms—who require non-invasive respiratory support modalities (such as HFNO or NIV), or those requiring invasive mechanical ventilation without multi-organ dysfunction syndrome (MODS), should be managed in—or transferred to—a level II ICU when a level III ICU is unavailable or overwhelmed. In such cases, support through a Tele-ICU system may provide significant benefit.	88.11%	Consensus in the second round	1	8	11.82%
3.	Management of the most severe forms of ARDS (particularly in patients with multi-organ dysfunction, hemodynamic instability, and/or those requiring advanced life support measures such as V-V ECMO) should be conducted in specialized centers such as Level III ICUs. In countries classified as LRS that lack such centers, health and political authorities should be engaged and encouraged to recognize the necessity of establishing such facilities. In the early phase of developing such services, the use of Tele-ICU systems may provide meaningful support.	89.14%	Consensus in the second round	1	9	10.86%
4.	In LRS, developing standardized protocols for the safe transport of ARDS patients to higher levels of care should include establishing critical care retrieval teams, training transport personnel, ensuring the availability of appropriate transport ventilators, monitoring equipment, and other essential resources for advanced life support during transport, as well as adding communication and coordination between referring and receiving centers.	83.38%	Consensus in the first round	1	9	16.62%
5.	The transfer of ARDS patients from regional centers (level I or II ICUs) to tertiary centers (level III ICUs) should follow a standardized protocol developed and endorsed by local health authorities. This protocol should be publicly available to all stakeholders and must include clear guidance on inter-facility coordination, patient stabilization before transfer, appropriate medical transport resources, and the continuity of medical documentation.	85.69%	Consensus in the first round	1	9	14.30%
6.	Coordination of ARDS patient transfer should occur directly between the receiving physician at the level III ICU and the responsible physician, typically an anesthesiologist or intensivist, at the referring level I or II ICU.	80.29%	Consensus in the first round	1	9	19.70%
7.	In LRS, transporting ARDS patients to Level III ICUs is often challenging due to financial and human resource constraints. Health and political authorities should be encouraged to develop safe transport systems for critically ill patients. When feasible, a mobile expert team from the Level III ICU equipped with advanced support (e.g., V-V ECMO), should be deployed to stabilize and retrieve the patient. Where transport is not feasible, Tele-ICU support may provide critical guidance to lower-level facilities.	81.58%	Consensus in the second round	1	9	18.42%
8.	Safe transfer, as part of ARDS management, requires adequate transport vehicles, appropriate medical equipment, a trained team, and complete medical documentation to ensure both clinical and legal continuity.	84.26%	Consensus in the first round	1	9	15.74%
9.	Ethical considerations, in accordance with local laws, should guide decisions regarding the transport of ARDS patients to Level III ICUs, particularly to avoid transfers of those with a very poor prognosis, while balancing the potential benefits of higher-level care against the associated burdens and costs.	88.54%	Consensus in the second round	1	9	11.46%
**N^o^**	**Second domain: *Diagnostic tools for the identification of ARDS in LRS* statements/recommendations**	**Stability**	**Consensus status**	**IQR**	**Median**	
1.	In the absence of blood gas analyses, ARDS could be diagnosed using SpO₂/FiO₂ ratio, in association with bilateral infiltrates on chest X-ray or lung ultrasound. However, the usefulness of such a definition remains to be demonstrated and should not routinely replace PaO₂/FiO₂ ratio.	80.03%	Consensus in the first round	2	8	19.97%
2.	Lung ultrasound (LUS), when performed by a trained physician, is a valuable diagnostic tool for ARDS, particularly in emergency departments or shock rooms within LRS, where access to chest X-rays may be limited. LUS enables rapid bedside assessment of both pulmonary and cardiac function.	85.43%	Consensus in the second round	1	8	14.57%
3.	The adoption of ultrasound in LRS remains below expected levels and warrants broader promotion. Health and educational authorities are strongly encouraged to integrate ultrasound training across all tiers of medical education - including undergraduate curricula, residency programs, and fellowship - to develop sustainable diagnostic capacity. The implementation of Tele-ICU platforms can play a crucial role in this process by supporting remote training, supervision, and real-time clinical decision-making.	81.67%	Consensus in the second round	2	8	18.33%
4.	Ultrasound provides a valuable, non-invasive method for assessing volume status in ARDS, especially in LRS, as long as adequately trained personnel are available. To ensure sustainable implementation, health and educational authorities should prioritize ongoing ultrasound education, with Tele-ICU support playing a crucial role during the training and scale-up phase.	81.40%	Consensus in the second round	1	8	17.43
5.	The Berlin Definition has long served - and continues to serve - as the foundational framework for diagnosing acute respiratory distress syndrome (ARDS). However, the challenges highlighted by the COVID-19 pandemic have exposed important limitations of this definition. Specifically, it fails to account for the critical roles of non-invasive ventilation (NIV and HFNO) and point-of-care (POC) ultrasound in the modern diagnosis, monitoring, and management of ARDS. A redefinition of ARDS is warranted to integrate these widely adopted clinical practices and recent advancements, to improve diagnostic accuracy and patient outcomes.	84.29%	Consensus in the second round	1	9	15.71%

Abbreviations: ARDS - Acute Respiratory Distress Syndrome, ICU – Intensive Care Unit, HFNO - high-flow nasal oxygen, NIV – non-invasive ventilation, MODS - multi-organ dysfunction syndrome, V-V ECMO - veno-venous extracorporeal membrane oxygenation, LRS - low resource settings, SpO₂ - Peripheral Capillary Oxygen Saturation, FiO₂ - Fraction of Inspired Oxygen, LUS – lung ultrasound, PaO₂- Arterial Partial Pressure of Oxygen.

**Table 2:** List of consensus statements/recommendations for the third domain across the first and second Delphi rounds of voting.

N^o^	Statements/Recommendations	Stability	Consensus status	IQR	Median	COV
	**Third domain: *Treatment—respiratory support***					
1.	In patients with mild to moderate ARDS, non-invasive ventilation (NIV) and high-flow nasal cannula (HFNO) should be considered first-line therapies when hemodynamics are stable and there are no contraindications. Careful patient selection and close monitoring are essential to ensure timely escalation of care at the first signs of clinical deterioration.	87.08%	Consensus in the second round	1	9	12.92%
2.	Upon admission to the intensive care unit, both the patient with ARDS (if able to participate) and their family should be informed about the risks, costs, burdens, and alternatives to intubation, including the potential need for prolonged mechanical ventilation and tracheostomy. They should have the opportunity to participate in decisions regarding the forgoing of life-sustaining treatment, in accordance with local laws and regulations.	83.02%	Consensus in the second round	1	9	16.98%
3.	In LRS, non-invasive ventilation (NIV/HFNO) may be used for patients with severe ARDS for a short period to provide valuable initial time to stabilize the patient, ensure adequate pre-oxygenation, and create conditions for safe intubation. A period of 1–2 hours is an appropriate timeframe to wait for patient stabilization, with continuous monitoring and readiness for rapid and timely intubation if needed.	85.74%	Consensus in the second round	1	9	14.26%
4.	The decision to intubate ARDS patients should be based on clinical response to non-invasive support and the likelihood of benefit, taking into account ethical considerations and local legal frameworks.	80.45%	Consensus in the first round	2	8	19.66%
5.	In LRS, where experience with various ventilation modes may be limited, the mode most familiar to the physician and medical team should be used to ensure safe and effective care.	80.00%	Consent in the first round	1	8	20.14%
6.	In intubated and mechanically ventilated ARDS patients, low tidal volume ventilation (6−8 mL/kg of predicted body weight) should be used as a safe and effective lung-protective strategy.	82.38%	Consensus in the first round	0	9	17.62%
7.	To substantially reduce ventilation errors and improve the implementation of lung-protective strategies, simple nomograms displaying predicted body weight and corresponding tidal volume settings for men and women, as well as toxic^∗^ FiO_2_ concentrations and dangerous^∗∗^ Ppeak and Pplat levels, should be physically attached and prominently visible on all ventilators.	85.44%	Consensus in the second round	1	9	14.56%
8.	In mechanically ventilated ARDS patients, the initial PEEP should be set within the range of 5−10 cm H_2_O, avoiding levels below 5 cm H_2_O, and subsequently titrated according to the patients clinical status, including oxygenation and hemodynamic parameters, to maintain a plateau pressure below 30 cm H_2_O. It is important to consider that in patients with left ventricular failure or right heart failure, PEEP levels may need to be adjusted upward or downward based on clinical response and tolerance.	92.34%	Consensus in the second round	1	9	7.66%
9.	All intubated ARDS patients should have plateau pressure measured as part of their ventilatory management to guide lung-protective strategies. An inspiratory hold of 2 seconds should be performed to obtain accurate measurements (every 6−8 hours or after any major ventilator adjustment).	80.93%	Consensus in the first round	2	8	19.07%
10.	For hemodynamically stable patients with ARDS, following an assessment of ventilator recruitability, a brief recruitment manoeuvre lasting less than 40 seconds should be considered. Subsequently, ventilator settings for PEEP and FiO_2_ should be adjusted to the minimal effective levels necessary to maintain optimal oxygenation. Careful monitoring of the patients clinical status during and after the recruitment manoeuvre is essential for the timely identification of any adverse events.	85.45%	Consensus in the second round	0	9	14.51%
11.	Prolonged recruitment manoeuvres lasting more than 60 seconds should be avoided in low-resource settings (LRS) due to the increased risk of serious adverse effects, including hemodynamic instability, barotrauma, and biotrauma.	86.08%	Consensus in the second round	1	9	13.92%
12.	Prone positioning should be considered in patients with moderate to severe ARDS and severe hypoxemia (defined as a PaO_2_/FiO_2_ ratio below 150 or an SpO_2_/FiO_2_ ratio below 200), provided that all necessary conditions related to staff expertise and patient safety are adequately met.	81.81%	Consensus in the second round	1	9	18.18%
13.	If prone positioning leads to improved oxygenation and arterial blood gases (ABG), intubated patients with moderate to severe ARDS in low-resource settings (LRS) should be maintained in the prone position for more than 12 hours per day, provided that all necessary conditions related to staff expertise and patient safety are met.	86.04%	Consensus in the second round	1	8	13.96%
14.	The prone position requires trained teams to ensure its safe implementation, and staff education on this manoeuvre is essential.	84.60%	Consensus in the first round	1	9	15.39%

Abbreviations: ARDS - Acute Respiratory Distress Syndrome, ICU – Intensive Care Unit, HFNO - high-flow nasal oxygen, NIV – non-invasive ventilation, LRS - low resource settings, SpO₂ - Peripheral Capillary Oxygen Saturation, FiO₂ - Fraction of Inspired Oxygen, LUS – lung ultrasound, PaO₂- Arterial Partial Pressure of Oxygen, POC - point-of-care, PEEP – positive end expiratory pressure, ABG - arterial blood gases, ^∗^ The term “toxic FiO₂ concentrations” refers to prolonged exposure to high inspired oxygen concentrations when lower FiO₂ levels could be achieved through optimization of lung-protective strategies and oxygenation targets, ^∗∗^ The term “dangerous Ppeak and Pplat levels” refers to airway pressure levels associated with an increased risk of ventilator-induced lung injury (VILI) and emphasizes adherence to lung-protective ventilation principles rather than a specific pressure threshold.

**Table 3:** List of consensus statements/recommendations for the fourth, fifth, sixth, seventh, and eighth domains across the first and second Delphi rounds of voting.

N^o^	Statements/Recommendations	Stability	Consensus status	IQR	Median	CoV
	**Fourth domain: *other supportive therapies***					
1.	In patients with ARDS, the duration of corticosteroid therapy should not exceed 7–10 days, which corresponds to the exudative phase of the syndrome. Tapering is unnecessary when corticosteroids are administered within this timeframe.	82.73%	Consensus in the second round	2	8	17.27%
2.	In patients with moderate to severe ARDS, consideration should be given to using neuromuscular blocking agents (NMBAs) during the first 48 hours in cases of persistent ventilator dyssynchrony despite deep sedation. If muscle relaxation remains necessary after this period, based on the clinical condition, ventilator dyssynchrony, and arterial blood gas analysis, intermittent administration of NMBAs is preferred over continuous infusion.	80.29%	Consensus in the second round	2	8	19.70%
3.	Conservative fluid management and achieving a negative fluid balance have demonstrated an advantage over liberal fluid administration in the treatment of stable ARDS. Fluid balance should be closely monitored, aiming for either a neutral or negative balance. If necessary, the use of diuretics or available dialysis procedures can be considered to achieve this goal.	81.95%	Consensus in the first round	1	9	18.05%
4.	The use of V-V ECMO in the treatment of ARDS in LRS should be limited to centers with the highest levels of experience and personnel, with the prerequisite that there is the possibility of adequate and safe transport of these patients (e.g., by special ambulance, helicopter, etc.)."	83.53%	Consensus in the first round	1	9	16.47%
**N^o^**	**Fifth domain: *Sedation Strategies***	**Stability**	**Consensus status**	**IQR**	**Median**	**CoV**
1.	Use the most readily available short-acting sedative (ideally propofol) with basic monitoring (e.g., observation of movement, facial expressions). Assess sedation clinically: monitor for spontaneous movements, eye opening, facial grimacing, and breathing against the ventilator, using monitoring scales with which clinicians in low-resource settings are familiar (e.g., RASS, BPS, etc.).	82.98%	Consensus in the first round	2	8	17.02%
2.	Avoid oversedation whenever possible by using sedation depth monitoring and daily wake-up tests, if ventilator parameters allow, as excessive sedation can increase the risk of prolonged mechanical ventilation and delirium.	80.14%	Consensus in the first round	1	9	19.86%
3.	Prolonged sedation increases the risk of withdrawal syndrome and ICU delirium, particularly with extensive use of benzodiazepines, necessitating careful management and gradual weaning. The presence and support of family members, when feasible, along with other supportive measures such as optimal nutrition and targeted physical therapy, can significantly reduce the incidence of these complications.	88.01%	Consensus in the second round	2	8	26.17%
**N^o^**	**Sixth domain: *Weaning***	**Stability**	**Consensus status**	**IQR**	**Median**	**CoV**
1.	In LRS, spontaneous breathing trials (SBT), preferably using pressure support ventilation, should be the primary method for weaning patients from MV. Weaning may be initiated when clinically appropriate by the attending physician^∗^ - ideally when the patient has stable oxygenation (PEEP 8–10 cm H_2_O, FiO2 < 50%), and in the absence of delirium or significant hemodynamic instability. SBTs should be performed daily when there are no contraindications, with a test duration of 20 to 60 min. During each trial, patients must be closely monitored for signs of intolerance or clinical deterioration.	85.43%	Consensus in the second round	1	8	24.26%
2.	In patients at high risk for reintubation - such as those older than 65 years with multiple comorbidities (e.g. APACHE II **>** 12), post-extubation hypercapnia (PaCO2 **>** 45 mmHg), ineffective cough, or a history of multiple weaning failures – non-invasive ventilatory support (NIV or HFNO) should be initiated within the first 24 hours following extubation. During this period, patients should be closely monitored and regularly reassessed.	80%	Consensus in the second round	1	8	20.02%
3.	Close monitoring of oxygenation (SpO_2_, PaO_2_/FiO_2_), ventilation (pH, PaCO_2_), and signs of increased work of breathing is crucial during the weaning process to detect failure early and prevent complications.	80.14%	Consensus in the first round	1	9	19.86%
**N^o^**	**Seventh domain: *Nutrition***	**Stability**	**Consensus status**	**IQR**	**Median**	**CoV**
1.	Critically ill patients, especially in low-resource setting, are at risk of malnutrition and vitamin deficiencies. Risk assessment should be performed using validated tools such as the mNUTRIC score, NRS-2002, or Subjective Global Assessment (SGA) to identify patients at risk of malnutrition.	82.20%	Consensus in the second round	1	9	17.80%
2.	In LRS, where indirect calorimetry or other precise assessment tools are unavailable, nutritional needs in ARDS patients can be estimated at 20–25 kcal/kg/day for energy and 1.2−2 g/kg/day for protein. If predictive equations are used to estimate the energy need, hypocaloric nutrition (below 70% estimated needs) should be preferred over isocaloric nutrition for the first week of ICU stay.	84.06%	Consensus in the second round	2	8	15.93%
3.	In hemodynamically stable patients with ARDS, enteral nutrition (EN) should be initiated within 48 hours of ICU admission. Initial nutrition should begin with a low-dose trophic approach (6–10 kcal/kg/day), with progressive advancement based on patient tolerance. Caloric and protein intake targets should be approached gradually, increasing intake by 5–10% of the calculated total caloric needs per day, aiming to reach full requirements by days 5–7 of ICU stay, as tolerated. Parenteral nutrition (PN) should only be considered when EN is contraindicated or not tolerated after 3−7 days.	80.24%	Consensus in the second round	1	9	19.76%
4.	Enteral nutrition via nasogastric tube should be the standard approach. In LRS settings, where feeding pumps are often unavailable and there is a frequent shortage of nursing staff, gravity-assisted feeding is a practical alternative. Feeding volumes should be adjusted according to patient tolerance, and regular assessment for intolerance (gastric residual volume, abdominal distension, vomiting, and diarrhea) remains essential.	87.07%	Consensus in the second round	1	8	12.93%
**N^o^**	**Eight domain: *Management of complications***	**Stability**	**Consensus status**	**IQR**	**Median**	**CoV**
1.	In ARDS patients on mechanical ventilation, delirium prevention should primarily focus on minimizing sedation to the lowest necessary levels, ensuring adequate analgesia, implementing early extubation strategies or early tracheotomy, and promoting daily cognitive stimulation, as well as proper sleep-wake cycle management. All available resources in LRS, such as early rehabilitation, should be engaged to support delirium prevention.	81.15%	Consensus in the first round	1	9	18.85%
2.	In Low Resource Settings (LRS), preventing hospital-acquired infections in ARDS patients on mechanical ventilation should primarily focus on basic infection control principles, such as strict hand hygiene, minimizing unnecessary interventions, and avoiding the opening of the ventilator circuit.	80%	Consensus in the first round	1	9	25.57%

ARDS - Acute Respiratory Distress Syndrome, ICU – Intensive Care Unit, HFNO - high-flow nasal oxygen, MV – mechanical ventilation, NIV – non-invasive ventilation, V-V ECMO - veno-venous extracorporeal membrane oxygenation, LRS - low resource settings, SpO₂ - Peripheral Capillary Oxygen Saturation, FiO₂ - Fraction of Inspired Oxygen, LUS – lung ultrasound, PaO₂- Arterial Partial Pressure of Oxygen, NMBAs - neuromuscular blocking agents, SBT - spontaneous breathing trials, EN - enteral nutrition, PN - parenteral nutrition, SGA - subjective Global Assessment, PaCO_2_ - Arterial Partial Pressure of Carbon Dioxide, APACHE II - Acute Physiology and Chronic Health Evaluation II, PEEP – positive end expiratory pressure, RASS - the Richmond Agitation–Sedation Scale, BPS - Behavioral Pain Scale, mNUTRIC score - Modified Nutrition Risk in the Critically Ill Score, NRS-2002 - Nutritional Risk Screening 2002, ^∗^ In many LRS, weaning decisions are primarily physician-led because critical care nurses, respiratory therapists, and dedicated weaning teams are often unavailable.

The Delphi process concluded with 44 of 56 proposed statements reaching consensus, while 12 remained non-consensus findings. These included two statements on respiratory support, seven on other supportive therapies, and one each on weaning, nutrition, and complication management. Analysis of panellist comments indicated that disagreement mainly reflected uncertainty in the supporting evidence, variation in local feasibility and resource availability, differences in safety and monitoring requirements, and the inclusion of multiple concepts within individual statements. Detailed voting results, statement evolution, panellist comments, and the final status of all consensus and non-consensus statements are provided in the Supplementary Material. Fig. 3 summarizes the alignment between the present Delphi statements, the ESICM ARDS guidelines, and other international recommendations relevant to ARDS management in LRS.

## Discussion

To our knowledge, this is the first global Delphi study convened among international experts to reach a consensus on ARDS management in LRS. The panel specifically addressed barriers (resource-variability) such as oxygen supply limitations, staffing shortages, lack of arterial blood gas analysis, limited radiography, and transport constraints, which are more characteristic of the poorest settings within the LRS spectrum, where the affected population is approximately tenfold smaller. Consensus statements mostly aligned with ESICM recommendations, although our statements included additional guidance for infrastructure and organization, diagnostic tools for ARDS detection, sedation strategies, weaning, nutrition, and management of complications (Fig. 3). However, consensus was more difficult to achieve for recommendations addressing interventions whose feasibility depended heavily on local infrastructure, workforce capacity, and resource availability across participating healthcare systems.

The 12 non-consensus statements represent important areas of uncertainty rather than unsuccessful outcomes of the Delphi process. Disagreement involved awake prone positioning and criteria for repeated proning, selected supportive therapies, including corticosteroids, neuromuscular blockade, inhaled vasodilators, and early or percutaneous tracheostomy, as well as specific approaches to weaning, locally prepared enteral formulas, and pressure-injury prevention. Panellist comments suggested that non-consensus reflected limited or context-dependent evidence, variation in staff expertise and monitoring capacity, safety concerns, differences in resource availability, and, in some cases, the inclusion of multiple concepts within a single statement. These findings identify priorities for future context-specific feasibility and implementation research and suggest that complex statements should be separated into individual components in future Delphi studies.

**Fig. 2:**
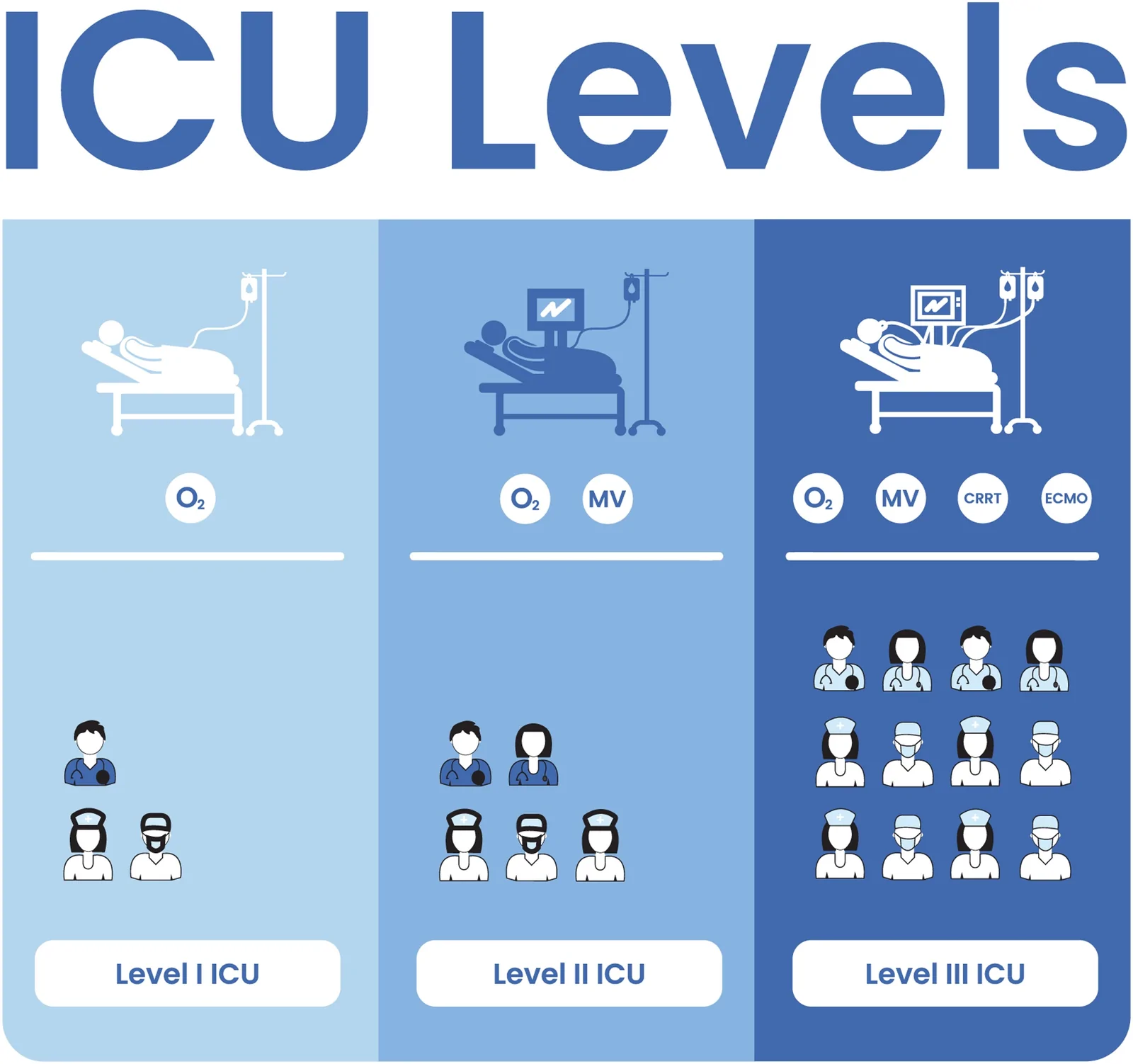
Levels of ICU.

The domain ***Critical Care Infrastructure Requirements for ARDS Management in LRS*** emphasizes the importance of defining ICU levels and clarifying their respective roles, functions, and implementation within LRS (Fig. 2). While previous literature has rarely examined the impact of ICU level classification on ARDS outcomes, most studies have focused primarily on the level of care required for patients receiving V-V ECMO [13,25]. This consensus report introduces a structured framework, particularly relevant for health systems where such stratification is absent. These recommendations are therefore not only crucial for improving ARDS management but also for strengthening critical care organization and capacity building in LRS [26]. This is particularly relevant in many LRS, where transfer to higher-level centres is often limited by the lack of available ICU beds, transport resources, and safe referral pathways. Importantly, growing evidence suggests that multidisciplinary models of organization and care delivery in LRS are associated with significant improvements in outcomes among critically ill patients, as well as enhanced overall health-system performance [27]. Beyond equipment and infrastructure limitations, the establishment of well-organized multidisciplinary care teams may represent a practical and achievable approach to strengthening critical care services and improving the implementation of evidence-based ARDS management in LRS.

**Fig. 3:**
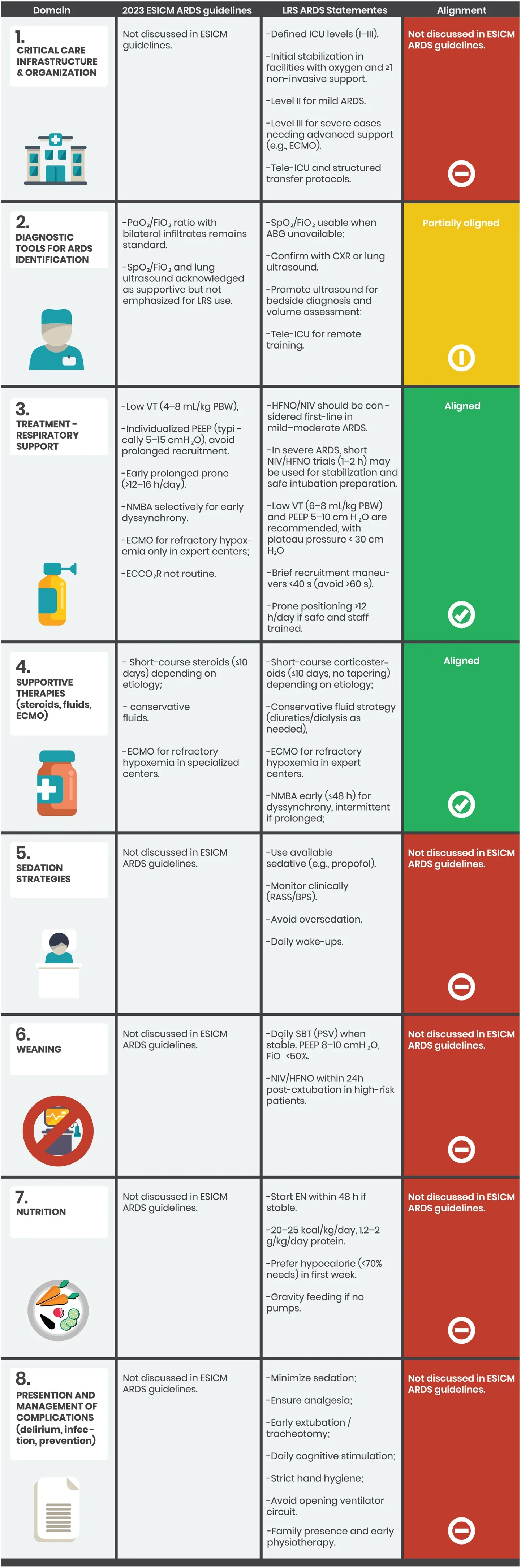
Comparison between the ESICM ARDS guidelines and the Delphi consensus statements for ARDS management in LRS across eight key domains.

Within the ***respiratory support domain***, it is important to emphasize that Fig. 2 positions HFNC and NIV within Level I ICU care, including High Dependency Units (HDUs), thereby illustrating the appropriate role of these therapeutic modalities within the healthcare system and helping reserve higher-level ICU capacity for patients requiring invasive mechanical ventilation. Although a clear consensus was achieved for Respiratory Support Domain statement #5, it is important to emphasize that, in addition to selecting ventilatory modes with which clinicians are most familiar, these modes should also be capable of delivering and maintaining lung-protective ventilation strategies in patients with ARDS [28]. Regarding Statement #8 within the Respiratory Support Domain, it is important to recognize that although plateau pressure remains a key target of lung-protective ventilation and is supported by current guidelines, driving pressure may provide additional physiological insight, particularly in patients requiring higher levels of PEEP. Accordingly, when feasible, driving pressure may serve as a useful complementary parameter to optimize ventilatory management while maintaining established lung-protective ventilation principles [29,30]. Although recruitment maneuvers (statements #10 and #11) may improve oxygenation in selected patients, evidence from recent trials suggests that aggressive recruitment strategies using high airway pressures may be associated with increased harm [31]. This consideration may be particularly important in LRS, where the capacity to manage procedure-related complications is often limited. The successful implementation of all respiratory support strategies ultimately depends on a reliable oxygen ecosystem, including adequate oxygen supply, monitoring of oxygen purity, and sufficient flow delivery capacity. This consideration is particularly relevant in the most LRS, where interruptions in oxygen supply and reliance on cylinders or oxygen concentrators remain common. Nevertheless, these settings account for only a small proportion of LRS critical care environments worldwide, many of which are located in middle- and upper-middle-income countries.

The domain addressing ***diagnostic tools for ARDS identification in LRS*** reveals only partial alignment between ESICM recommendations and LRS consensus statements. ESICM guidelines defined ARDS using the PaO₂/FiO₂ ratio with bilateral infiltrates as the standard diagnosis and acknowledge the help of SpO₂/FiO₂ and lung ultrasound merely as supportive tools, without addressing their feasibility or utility in LRS, a position consistent with several prior studies [[32], [33], [34], [35], [36], [37], [38]]. In contrast, the LRS statements emphasize the practicality of these alternative tools, promoting SpO₂/FiO₂ and lung ultrasound as feasible and cost-effective options in environments lacking tools for arterial blood gas analysis or radiographic imaging. These recommendations are aligned with the recently published Global Definition of ARDS for resource-constrained settings [[35], [36], [37], [38], [39]]. Nevertheless, important limitations of the SpO₂/FiO₂ ratio should be acknowledged, including reduced accuracy in patients with shock and the potential influence of skin pigmentation on pulse oximetry measurements. Therefore, further validation across diverse populations and healthcare settings remains warranted. Furthermore, the integration of telemedicine and remote education (e.g., the CERTAIN checklist) was highlighted as an additional means of enhancing diagnostic capacity and clinician training in LRS, with growing evidence demonstrating that these approaches are cost-effective, scalable, and feasible across resource-constrained environments. However, the effectiveness and sustainability of telemedicine programs may vary across LRS because of differences in technological infrastructure, available resources, and access to ongoing expert support. Structured programs such as CERTAIN provide a framework for standardized multidisciplinary care, clinician education, and quality improvement, helping to reduce practice variability and support the implementation of evidence-based critical care in low-resource settings [40,41]. Regarding the use of neuromuscular blocking agents (***Other supportive therapies domain*** *#2*) in patients with moderate-to-severe ARDS, it is important to emphasize that adequate deep sedation and analgesia are essential prerequisites for their safe administration. Although continuous sedative infusions may not always be readily available in some extremely resource-limited settings, every effort should be made to ensure an appropriate depth of sedation before and during neuromuscular blockade to prevent patient distress, awareness, and ventilator dyssynchrony.

The domain addressing ***sedation*** and ***weaning*** in ARDS highlights a gap in ESICM guidelines, which provide no specific recommendations on these aspects of care. The LRS consensus proposes the use of propofol as the preferred sedative agent, despite its known potential side effects, along with clinical monitoring using the Richmond Agitation–Sedation Scale (RASS) and Behavioral Pain Scale (BPS) scores, avoidance of oversedation, and implementation of daily awakening trials. These measures are consistent with evidence from prior studies reporting that lighter, protocolized sedation, and daily awakening improve outcomes and facilitate earlier weaning in critically ill patients [[42], [43], [44], [45], [46]]. In addition, daily paired spontaneous awakening trials (SATs) and spontaneous breathing trials (SBTs) are evidence-based interventions that facilitate liberation from mechanical ventilation and may improve outcomes when incorporated into routine care [47]. Particular attention should be given to high-risk patients, in whom prophylactic NIV initiated immediately after extubation may be preferable to delayed application, as postponing noninvasive support until respiratory deterioration occurs may increase the risk of reintubation and adverse outcomes.

***Nutrition,*** as well as ***the prevention and management of complications,*** were not addressed in ESICM ARDS guidelines. The LRS expert consensus recommends initiating enteral nutrition (EN) within 48 hours if patients are hemodynamically stable, providing 1.2–2 kcal/kg/day, preferring a hypocaloric regimen (<70% of calculated needs) during the first week, and using gravity feeding when infusion pumps are unavailable. Although studies focusing exclusively on nutritional strategies in ARDS are limited, particularly in LRS, these recommendations are generally consistent with broader critical care nutrition guidelines and evidence supporting early, cautious initiation of enteral feeding in critically ill patients [[48], [49], [50]]. It is important to note that no consensus was achieved regarding the preparation of enteral formulas in hospital settings, although many experts emphasized this as a critical and unmet need, particularly given that a substantial proportion of patients in LRS are already malnourished at the time of ICU admission. This consideration may be particularly important in LRS, where pre-existing malnutrition is common and may substantially influence the clinical course and outcomes of critically ill patients. Therefore, beyond the provision of enteral nutrition, early identification and appropriate management of underlying malnutrition should be regarded as an important component of ARDS care in LRS. The domain addressing the prevention and management of complications in ARDS patients within LRS emphasizes early mobilization, infection control, and patient-centered supportive care as key components of improved outcomes. Building on the previously discussed principles of sedation and analgesia management [[42], [43], [44], [45], [46]], the consensus highlighted the benefits of early weaning from mechanical ventilation or tracheotomy [[51], [52], [53]], although no agreement was reached regarding the optimal timing (*early vs. late*), with many LRS experts favoring tracheotomy as early as feasible. Daily cognitive stimulation, family presence, and regular physiotherapy were recognized as essential components of comprehensive care, consistent with studies showing their role in reducing delirium and accelerating recovery [54,55]. Maintaining strict hand hygiene and avoiding unnecessary opening of the ventilator circuit were emphasized as practical and cost-effective measures to prevent hospital-acquired infections [56,57].

Our study has several strengths. It included an international panel of experts with extensive experience in ARDS management across diverse healthcare and resource settings, addressed important knowledge gaps relevant to ARDS care in LRS, and used an anonymous Delphi process to reduce group influence. Nevertheless, several limitations should be acknowledged. The panel was relatively small and selected through purposive, convenience-based sampling and professional networks, which may have introduced selection bias and contributed to regional and gender imbalances. Limited gender diversity may also have restricted the range of perspectives represented and reduced the external validity of the consensus findings. Although approximately two-thirds of participants worked in, or had substantial experience in, LRS, the heterogeneity of participating healthcare systems, including differences in infrastructure, workforce, diagnostics, therapies, and local practice, may have limited generalizability and contributed to persistent disagreement in some domains. Although the relatively low and variable clinical exposure to ARDS across healthcare settings supports the use of a Delphi approach, differences in individual panellists’ case volume and experience, which were not systematically quantified, may have influenced the diversity of perspectives, consensus stability, and external validity of the findings. The panel included only physicians, with no participation from nurses, physiotherapists, patients, or public representatives, thereby limiting multidisciplinary and access-to-care perspectives. Only three Delphi rounds were conducted, and further rounds might have clarified unresolved issues. In addition, Steering Committee refinement of statement wording between rounds may have encouraged convergence, while some statements combined multiple clinical and organizational concepts, potentially producing ambiguous agreement ratings and limiting interpretation of consensus and non-consensus findings. The Delphi process also did not specifically examine resource variability, multidisciplinary care systems, standardized monitoring, checklist-based approaches, or under-sedation practices in mechanically ventilated patients. Future context-specific clinical and implementation research in LRS should validate and further refine these statements.

Accordingly, these statements should be viewed as context-adapted practice statements, rather than prescriptive guidelines, that complement existing ESICM recommendations, draw on broader critical care literature and expert consensus where ARDS-specific evidence is limited, and are most applicable to facility-based low-resource settings with some degree of critical care capacity.

In conclusion, using a Delphi approach, world experts reached consensus on clinical practice statements for managing ARDS in LRS. These statements offer guidance for critical decision-making in patient care where evidence is limited and complement existing global guidelines. They provide practical recommendations in public health for managing critically ill patients with ARDS while awaiting ICU admission, during safe patient transfer, and for context-specific considerations in low-resource environments. Future research should evaluate the feasibility, implementation, and impact of these practice statements, as well as address remaining uncertainties.

## Authors contributions

The Steering Committee (SC) comprised OG, PK, BT, FST, JV, GG, LC, MO, and JD. The SC was responsible for developing the survey questionnaires for each round of the Delphi process, for analyzing the responses, and formulating the expert clinical practice statements derived from items that achieved both consensus and stability. Also, PK, OG, JV, FST, GG, LC, MO, and JD contributed to the conceptualization and design of the study, data curation, project administration, validation of underlying data, and drafting of the manuscript. BT served as the Delphi methodology expert, conducting the literature review and performing the formal statistical analysis. He was responsible for overseeing the Delphi study until consensus was achieved and did not have voting rights.

Approximately two-thirds of the panel members worked in low-resource settings or had substantial professional experience in resource-constrained critical care environments (IP, SD, RM, DM, AAH, BZ, JM, MJ, OT, RZ, OS, MB, FJM, AM, MK, VK, JL, RJ, SJ, DM, OEE, RM, RK, MJ, AEM, JP, AC, YA, OK, KD, DL, WA, NG, AA, RO, AZ, MK, JP, SA). These experts actively participated in all Delphi rounds, completing the survey questionnaires and providing insightful comments, which were instrumental in shaping and refining the final expert clinical practice statements.

## Funding

None.

## Data sharing statement

The data presented in this study are available on request from the corresponding author.

## Declaration of competing interest

The authors report no conflicts of interest related to this work.
