# Supplementary material for: Consensus statement for managing patients with ARDS in low-resource settings: a Delphi study
Source: Ann Intensive Care. 2026 Aug 27;16:100144. doi: 10.1016/j.aicoj.2026.100144 (PMC13564132; doi:10.1016/j.aicoj.2026.100144)
Supplement: Supplementary file 1 [file mmc1.docx]

**SUPPLEMENTAL FILE**

*This Supplemental File has been provided by the authors for additional information about their work*

**Consensus Statement for Managing Patients with ARDS in Low-Resource Settings: A Delphi Study**

Pedja Kovacevic^1,2*^, Jesús Villar^2^, Boris Tomic^1,2^, Fabio Silvio Taccone^3^, Giacomo Grasselli^5^, Luigi Camporota^6^, Marlies Ostermann^7^, Ivan Palibrk^8^, Rajesh Mishra^9^, Sasa Dragic^1,2^, Jose Diaz-Gomez^10^, Danica Momcicevic^1,2^, Ali Ait Hssain^11^, Biljana Zlojutro^1,2^, Jihad Mallat^12^, Milka Jandric^1,2^, Owoniya Temitope^13^, Roman Zahorec^14^, Omar Shahed^15^, Mohamed Boussarsar^16^, F. Joachim Meyer^17^, Ahmed Mukhtar^18^, Marko Kutlesa^19^, Vladimir Krajinovic^19^, John Laffey^20^, Radmilo Jankovic^21^, Samir Jaber^22^, Dejan Markovic^23^, Oluwayemisi Esther Ekor^24^, Christian Owoo^25^, Rotimi Muyiwa^26^, Rihard Knafelj^27^, Matija Jurjevic^28^, Natasa Milivojevic^29^, Ahmed El Masry^30^, Jasminka Persec^31^, Anusha Cherian^32^, Younes Aissaoui^33^, Oguz Kilickaya^34^, Kent Doi^35^, Daniel Lovric^36^, Wael Alqassem^37^, Dafna Shai Garcia^38^, Natasa Gocic^39^, Aderonke Adesiyan^40^, Reuben Okioma^41^, Ayalew Zewdie^42^, Mumbere Kigayi^43^, Subhash Acharya^44^, Ognjen Gajic^45^

From

1- *University Clinical Centre of the Republic of Srpska, Medical Intensive Care Unit, Banja Luka, the Republic of Srpska, Bosnia and Herzegovina.*
2- *Faculty of Medicine, University of Banja Luka, Banja Luka, the Republic of Srpska, Bosnia and Herzegovina.*
3- *Research Unit at Hospital Universitario Dr. Negrín, Fundación Canaria Instituto de Investigación Sanitaria de Canarias, Las Palmas, Spain; CIBER de Enfermedades Respiratorias, Instituto de Salud Carlos III, Madrid, Spain; Faculty of Health Sciences, Universidad del Atlántico Medio, Tafira, Las Palmas. Spain; Li Ka Shing Knowledge Institute, St. Michael’s Hospital, Toronto, Canada.*
4- *Department of Intensive Care, Hôpital Universitaire de Bruxelles (HUB), Université Libre de Bruxelles (ULB), Brussels, Belgium.*
5- *Department of Emergency, Fondazione IRCCS Ca’ Granda Ospedale Maggiore Policlinico, Milan, Italy; Department of Pathophysiology and Transplantation, University of Milan, Milan, Italy*
6- *Department of Adult Critical Care, Guy's and St Thomas' NHS Foundation Trust, London, SE1 7EH, UK.*
7- *Department of Critical Care, King's College London, Guy's and St Thomas' NHS Foundation Trust, London, UK.*
8- *Department of Anesthesiology, Reanimatology and Intensive Care, Clinic for Abdominal Surgery, University Clinical Centre of Serbia, 11000 Belgrade, Serbia.*
9- *Critical Care, Shaibya Comprehensive Care Clinic, Ahmedabad, Gujarat, India, ESICM national representative of India.*
10- *Integrated Hospital Care Institute (Divisions of Anesthesiology, Critical Care Medicine, Emergency Medicine, Hospital Medicine, Infectious Disease, and Pulmonary Medicine), Cleveland Clinic Abu Dhabi, Abu Dhabi, United Arab Emirates.*
11- *Medical Intensive Care Unit, Hamad General Hospital, Doha, Qatar; Department of Medicine, Weill Cornell Medical College, Doha, Qatar.*
12- *Critical Care Division, Integrated Hospital Care Institute, Cleveland Clinic Abu Dhabi, 112412, Abu Dhabi, United Arab Emirates and Cleveland Clinic Lerner College of Medicine of Case Western Reserve University, Cleveland, OH, 44106, USA.*
13- *Department of Anaesthesia and Intensive Care Unit, Obafemi Awolowo University Teaching Hospital Complex (OAUTHC), Ile-Ife Nigeria.*
14- *Department of Anesthesiology and Intensive Medicine, Medical School, Comenius University, Heydukova 10, 812 50 Bratislava, Slovakia.*
15- *Division of Critical Care, University of Witwatersrand, Chris Hani Baragwanath Academic Hospital, Johannesburg, South Africa.*
16- *Medical Intensive Care Unit, Research Laboratory LR12SP09 "Heart Failure", Farhat Hached University Hospital, Sousse, Tunisia*
17- *München Klinik gGmbH and Medical Faculty, Lung Center Munich, University of Heidelberg, Heidelberg, Germany.*
18- *Department of Anesthesia and Critical Care, Cairo University, Cairo, Egypt*
19- *Department for Intensive Care, University Hospital for Infectious Diseases "Dr. Fran Mihaljević", 10000 Zagreb, Croatia.*
20- *Anaesthesia and Intensive Care Medicine, School of Medicine, Clinical Sciences Institute, Galway University Hospitals, University of Galway, Galway, Ireland.*
21- *University Clinical Center Niš, Department of Anesthesia and Intensive Therapy, Faculty of Medicine, University of Niš, Niš, Serbia.*
22- *Department of Anesthesia and Intensive Care Unit, Regional University Hospital of Montpellier, St-Eloi Hospital, University of Montpellier, PhyMedExp, INSERM U1046, CNRS UMR, Montpellier, France.*
23- *Department of Anesthesiology, Reanimatology and Intensive Care, Clinic for Cardiac Surgery, University Clinical Centre of Serbia, 11000 Belgrade, Serbia.*
24- *University of Cape Coast Ghana*
25- *Department of Anaesthesia, Lagos University Teaching Hospital, Idi-Araba, Lagos State, Nigeria.*
26- *Center for Internal Intensive Medicine, University Medical Center Ljubljana, Ljubljana, Slovenia.*
27- *Clinic of anesthesiology, resuscitation and intensive care, General Hospital Josip Bencevic, Slavonski Brod, Croatia.*
28- *Neurological Intensive Care Unit, University Clinical Centre of Ljubljana, Ljubljana, Slovenia.*
29- *Al Adan Hospital, Al Ahmadi, Kuwait*
30- *Clinical Department for Anesthesiology, Reanimatology and Intensive Care Medicine, University Hospital Dubrava, University of Zagreb School of Dental Medicine, 10000 Zagreb, Croatia.*
31- *Department of Anesthesiology and Critical Care, Jawaharlal Institute of Postgraduate Medical Education and Research, Puducherry, India*
32- *Department of Critical Care Medicine, Avicenna Military Hospital, Marrakesh, Morocco*
33- *Department of Medicine, Division of Pulmonary and Critical Care Medicine, Mayo Clinic, Rochester MN, USA.*
34- *Department of Emergency and Critical Care Medicine, The University of Tokyo, Tokyo, Japan.*
35- *Cardiology Clinic, University Hospital Center Zagreb, 10000 Zagreb, Croatia.*
36- *Critical Care Department, Prince Mohammed Bin Abdulaziz Hospital, Riyadh, Saudi Arabia,*
37- *University Clinical Centre of Vojvodina, Emergency Center, Novi Sad, Serbia.*
38- *Africa Health Science University, Rwanda*
39- *Department of Critical Care, The Nairobi Hospital, Nairobi, Kenya.*
40- *St Paul's Hospital Millenium Medical College, Addis Ababa, Ethiopia*
41- *Department of Anesthesiology and Intensive Care, HEAL Africa Tertiary Hospital, Goma, North Kivu, Democratic Republic of Congo*
42- *Department of Critical Care Medicine, MMC, Institute of Medicine, Tribhuvan University, Kathmandu, Nepal*
43- *Department of Medicine, Division of Pulmonary and Critical Care Medicine, Mayo Clinic, Rochester, MN, USA.*

*** Corresponding author:**

Pedja Kovacevic:

Dvanaest Beba bb, Banja Luka, 78000, Bosnia and Herzegovina,

tel. +38751342603; Fax: +38751342507. E-mail: [pedja.kovacevic@med.unibl.org](mailto:pedja.kovacevic@med.unibl.org),

**Electronic supplement**

Table of Contents

[1.	Methods	5](#_Toc231236794)

[2.	Results	3](#_Toc231236795)

[Demographics	3](#_Toc231236796)

[1.	Consent statement	3](#_Toc231236797)

[2.	The country where the voting member works	4](#_Toc231236798)

[3.	Years of experience working in an intensive care unit (ICU)	5](#_Toc231236799)

[4.	Age	6](#_Toc231236800)

[5.	Sex	7](#_Toc231236801)

[6.	Primary specialty	8](#_Toc231236802)

[7.	Official training in intensive care medicine	10](#_Toc231236803)

[8.	Type of intensive care unit	11](#_Toc231236804)

[9.	Type of institution experts are currently working in	12](#_Toc231236805)

[10.	Resource settings	13](#_Toc231236806)

[ROUND 1	14](#_Toc231236807)

[Domain 1: Critical Care Infrastructure Requirements for ARDS Management in Low-Resource Settings (LRS)	14](#_Toc231236808)

[Domain 2: Diagnostic tools for the identification of ARDS in LRS	21](#_Toc231236809)

[Domain 3: treatment – respiratory support	24](#_Toc231236810)

[Domain 4: other supportive therapies	39](#_Toc231236811)

[Domain 5: Sedation Strategies	46](#_Toc231236812)

[Domain 8: Management of complications	53](#_Toc231236813)

[ROUND 2	54](#_Toc231236814)

[Domain 1 - Critical Care Infrastructure Requirements for ARDS Management in Low-Resource Settings (LRS)	54](#_Toc231236815)

[Domain 2 - Diagnostic tools for the identification of ARDS in LRS	60](#_Toc231236816)

[Domain 3 - Treatment – respiratory support	66](#_Toc231236817)

[Domain 7 – Nutrition	84](#_Toc231236818)

[ROUND 3	98](#_Toc231236819)

[Domain 3 - Treatment – respiratory support	98](#_Toc231236820)

[Domain 4 - Other supportive therapies	101](#_Toc231236821)

[Domain 7 – Nutrition	103](#_Toc231236822)

[e-Table 10. ACCORD (ACurate Consensus Reporting Document) checklist for reporting Delphi studies	104](#_Toc231236823)

[RESEARCH IN CONTEXT	107](#_Toc231236824)

[e-Figure 11. Prisma Flowchart of studies included for Qualitative Evidence Synthesis	108](#_Toc231236825)

[e-Table 11. Final studies included for drafting statements of the Delphi rounds	109](#_Toc231236826)

[References	155](#_Toc231236827)

# 1. Methods

The initial, first round of the Delphi study was conducted using the commercial Delphi platform Surveylet (Calibrum Inc., Utah, U.S.A.). In that round, there were a total of 55 questions organized into 8 different domains. The questions were developed with the input from the Steering Committee, specifically established for this study. The question types included multiple-choice and Likert scale-based statements (choices ranged from 1 to 9 using a horizontal slider). The coefficient of variation was used as a measure of stability, with a stability threshold set at ≥ 80%, while the mean served as a measure of consensus.

An invitation to participate in the study was sent to 55 panelists in the field of intensive care medicine from various parts of the world and different resource settings. Developed systems (DS) (34.61%) and Low Resource Settings (LRS) (65.39%). The first round of the study took place over two weeks, from June 15 to June 30, 2025. From the 55 invited panelists, four did not respond to the invitation to participate (7.3%). A total of 52 participants completed the questionnaire. Fifty-two panelists, 92.3%, finished the first round within the designated timeframe. Two responded but did not complete the first round, 3.6% (and were therefore excluded from the rest of the study). One expert did not fill out the questionnaire correctly, 1.8%, despite clear instructions on how to complete it. A detailed report of all the questions and their comments is presented in full detail below.

**eTable 1.** Definitions and thresholds for consensus status

| **Consensus status** | **Definition and decision** |
| --- | --- |
| Consensus achieved | ≥80% of panellists rated the statement from 7 to 9 |
| Strong consensus | ≥90% of panellists rated the statement from 7 to 9 |
| Consensus not achieved in an interim round | 50–79% of panellists rated the statement from 7 to 9 in Round 1 or Round 2; the statement was revised based on panellist comments and resubmitted in the subsequent round |
| Dissent | <50% of panellists rated the statement from 7 to 9; the statement was discontinued and not resubmitted |
| Final non-consensus | <80% of panellists rated the statement from 7 to 9 after the final Delphi round; the statement was retained and reported as a final non-consensus finding |

*Panel Selection and Definition of Low-Resource Settings*

Panel members were selected using predefined expertise-based eligibility criteria established by the Steering Committee before participant recruitment. To be eligible for participation, candidates were required to meet all of the following criteria: (1) more than five years of clinical experience in the care of critically ill adult patients; (2) more than one year of professional work or active collaboration within low-resource critical care environments; and (3) more than five years of involvement in educational, research, quality-improvement, guideline-development, leadership, or other academic activities relevant to critical care medicine and/or ARDS. Additional consideration was given to previous participation in international consensus processes, professional society activities, and contributions to ARDS-related publications. Potential panelists were identified through professional networks and through recommendations from national and international critical care leaders, including representatives of scientific societies. The Steering Committee reviewed all nominations to ensure diversity in geographic representation, professional background, and experience across both resource-constrained and well-resourced healthcare systems.

For this study, classification as working in a low-resource setting (LRS) was based on self-declaration by participants. Before completing the survey, all panelists were provided with a standardized operational description of LRS through the Delphi platform. This definition described LRS as healthcare environments characterized by limitations in trained healthcare personnel, infrastructure, laboratory diagnostics, monitoring capabilities, equipment, and other resources required for the diagnosis and management of ARDS. Participants were asked to determine whether their primary clinical environment met these characteristics. This classification was not based solely on World Bank income categories, recognizing that resource limitations may exist within high-income countries and that well-resourced critical care units may also exist within low- and middle-income countries.

# 2.  Results

# Demographics

## 1. Consent statement

If you are happy to take part in this study, please tick I agree and then click on the arrow button at the bottom of the page to be taken to the next page.

|  | 1.     If you are happy to take part in this study, please tick I agree and then click on the arrow button at the bottom of the page to be taken to the next page. |
| --- | --- |
| I agree | 93.22% |
| Did not respond to the invitation | 6.78% |

**eFigure 1.** Consent statement

## 2. The country where the voting member works

**eFigure 2.** The country where the voting member works (United States, Germany, United Kingdom, Ireland, Japan, France, Belgium, Italy, Spain, Slovenia, Croatia, United Arab Emirates, Saudi Arabia, Kuwait, Slovakia, Chile, Panama, Libya, South Africa, Colombia, Serbia, Bosnia and Herzegovina, Tunisia, Egypt, India, Ghana, Nigeria, Kenya, Nepal, Ethiopia, Congo)

## 3. Years of experience working in an intensive care unit (ICU)

**eTable 2.** Years of experience working in an intensive care unit (ICU)

|  | **Experience** | **%** |
| --- | --- | --- |
| **Years of experience working in ICU** | 10-19 years | 54 % |
|  | 20-29 years | 39 % |
|  | 30-39 years | 6 % |
|  | above 40 years | 2 % |

|  | Years of experience working in intensive care unit |
| --- | --- |
| 10-19 years | 54% |
| 20-29 years | 39% |
| 30-39 years | 6% |
| above 40 years | 2% |

**eFigure 3.** Years of experience working in intensive care unit

## 4. Age

**eTable 3.** Age of experts

|  | **Years** | **%** |
| --- | --- | --- |
| **Age** | 30-39 years | 4% |
|  | 40-49 years | 48% |
|  | 50-60 years | 41% |
|  | Above 60 years | 7% |

|  | Age |
| --- | --- |
| 30-39 years | 4% |
| 40-49 years | 48% |
| 50-60 years | 41% |
| Above 60 years | 7% |

**eFigure 4.** Age of experts

## 5. Sex

**eTable 4.** Sex

| **Sex** | **n** | **%** |
| --- | --- | --- |
| Male | 42 | 78 |
| Female | 12 | 22 |

|  | Sex |
| --- | --- |
| Male | 78% |
| Female | 22% |

**eFigure 5**. Sex

## 6. Primary specialty

|  | Primary specialty |
| --- | --- |
| Anesthesiology | 41% |
| Internal medicine | 7% |
| Pulmonology | 6% |
| Neurology | 4% |
| Critical care medicine | 39% |
| Infectious diseases | 4% |

**eFigure 6.** Primary specialty

## 7. Official training in intensive care medicine

**eTable 5.** Official training in intensive care medicine

|  | Answer | % |
| --- | --- | --- |
| Official training in intensive care medicine (subspeciality) | Yes | 81.48 |
|  | No | 18.51 |

|  | Official training in intensive care medicine (subspeciality) |
| --- | --- |
| Yes | 81.48% |
| No | 18.51% |

**eFigure 7.** Official training in intensive care medicine

## 8. Type of intensive care unit

**eTable 6.** Type of intensive care unit

|  | ICU | % |
| --- | --- | --- |
| Type of intensive care unit | Medical ICU | 26 |
|  | Surgical ICU | 9 |
|  | Mix ICU | 59 |
|  | Research Unit | 2 |
|  | NeuroICU | 2 |
|  | Cardiovascular ICU | 2 |

|  | Type of intensive care unit |
| --- | --- |
| Medical ICU | 26% |
| Surgical ICU | 9% |
| Mix ICU | 59% |
| NeuroICU | 2% |
| Cardiovascular ICU | 2% |
| Research Unit | 2% |

**eFigure 8.** Type of intensive care unit

## 9. Type of institution experts are currently working in

**eTable 7.** Type of institution experts are working in

|  | **Type of hospital** | % |
| --- | --- | --- |
| **What type of institution you are currently working with?** | University-affiliated hospital | 76% |
|  | Public hospital | 7% |
|  | Private hospital | 17% |

|  | Type of hospital |
| --- | --- |
| University-affiliated hospital | 76% |
| Public hospital | 7% |
| Private hospital | 17% |

**eFigure 9.** Type of institution experts are working in

## 10. Resource settings

**eTable 8.** Resource settings

| Resource settings | n | % |
| --- | --- | --- |
| DS* | 18 | 34.61% |
| LRS** | 34 | 65.39% |
| In total | 52 | 100% |
| DS* – Developed systems  LRS** – Low resource settings | | |

|  | Resource settings |
| --- | --- |
| HIC | 34.61% |
| LRS | 65.39% |

**eFigure 10.** Resource settings

# ROUND 1

## Domain 1: Critical Care Infrastructure Requirements for ARDS Management in Low-Resource Settings (LRS)

**Statements**

**1.** Initial menagement, resuscitation and stabilization of all patients with ARDS (first 24-48 hours) should take place in healthcare facilities that offer oxygen therapy and at least one non-invasive respiratory support method such as a level I intensive care unit (ICU) or in a shock room.

| Status | Consensus | Agreement |
| --- | --- | --- |
| Consensus achieved | Yes | 81.07% |

**Anonymous comments**

1. At least in the High Dependence Unit (HDU).
2. The time course of ARDS will be different in each case, not all the patients should be treated at level I. Certainly, the initial treatment can be conducted at level I.
3. This is very important to optimize.
4. In some settings, resource constraints may require initial stabilization elsewhere before transfer, So, while this is ideal, it might not be always feasible everywhere. Also, depending on the clinical context (mild or severe ARDS), some patients might require high level of care.
5. It is important to emphasize that in there are LRS within the developed world. Certain areas or populations face limitations in accessing essential resources, such as adequate healthcare, education, or infrastructure, despite being located in a country with generally high standards of living. These limitations can be due to factors like geographic location, socioeconomic disparities, or specific vulnerabilities within the population.
6. This would ensure enhanced patient safety.
7. Such service available in few private places but not in public hospitals and considered very expensive
8. I would add “...and at least one non-invasive respiratory support method and short-term invasive MV...“

**2.** Patients with ARDS requiring mechanical ventilation should be managed in, or transferred to, a level II **ICU.**

| Status | Consensus | Agreement |
| --- | --- | --- |
| Consensus not achieved | No | 68.94% |

**Anonymous comments**

1. Shall be admitted to a level III ICU, not in Level II.
2. ARDS patients requiring MV should be transferred to level III ICU. I would accept to be transferred to level II ICU IF level II ICU had ventilatory support MV and trained personal.
3. Should be transferred to level III ICU.
4. MV available everywhere but with limited expertise.
5. This is must to get appropriate care and improve outcome.
6. Patients with ARDS requiring mechanical ventilation typically have moderate to severe disease and require IMV with lung protective strategies. The providers should be skilled with appropriate monitoring ventilatory parameters, proning, etc.
7. Weighted against potential complications during transport. If available, consider activating retrieval team.
8. Mechanical ventilation in an ARDS patient may imply a severe pathology which can not be managed in a level I ICU.

3. Management of the most severe forms of ARDS, particularly in patients with multi-organ dysfunction, hemodynamic instability, and/or those requiring advanced life support measures such as V-V ECMO treatment, should be conducted in specialized centers such as Level III ICUs. Countries classified as LRS that lack such centers should develop a strategy for transferring these patients to neighboring countries with the necessary capacity and specialized ARDS treatment centers.

| Status | Consensus | Agreement |
| --- | --- | --- |
| Consensus not achieved | No | 72.64% |

**Anonymous comments**

1. This may be difficult and not practical most of the times. In countries which cannot support these patients even helicopters or air transfer may involve a lot of logistic and administrative difficulties along with laws that may be different in other countries.
2. Cost of transporting patients is very high. This resource should rather be used for making those services available in these countries.
3. I suggest the most severe forms of ARDS should be managed in both level II and II ICUs. Be reminded that COST is a major issue in LRS and lack of insurance coverage. Most patients pay out of pocket and they can't afford medical evacuation to another facility with ECMO etc. There is also the associated risk with the transport of critically ill patients. Telemedicine can be introduced and advocacy to administrators initiated to get these ICUs adequately equipped. This can work out well if there is support from NGOs for the transfer.
4. It is economically more justified and cost-effective to make your own ECMO center.
5. Most critical cases of advanced severe ARDS should be treated in ICU 3. level. The transport of patients to the neighbor countries is impractical.

**4.**	In LRS, developing standardized protocols for the safe transport of ARDS patients to higher levels of care should include establishing critical care retrieval teams, training transport personnel, ensuring the availability of appropriate transport ventilators, monitoring equipment, and other essential resources for advanced life support during transport, as well as adding communication and coordination between referring and receiving centers.

| Status | Consensus | Agreement |
| --- | --- | --- |
| Consensus achieved | Yes | 83.38% |

**Anonymous comments**

1. These resources should be assigned to training and building capacity in each of the LRS countries
2. That is certainly necessary.
3. I fully agree.
4. Completely agree that protocols help to take timely decisions.

**5.**	The transfer of ARDS patients from regional centers (level I or II ICUs) to tertiary centers (level III ICUs) should follow a standardized protocol developed and endorsed by local health authorities. This protocol should be publicly available to all stakeholders and must include clear guidance on inter-facility coordination, patient stabilization before transfer, appropriate medical transport resources, and the continuity of medical documentation.

| Status | Consensus | Agreement |
| --- | --- | --- |
| Consensus achieved | Yes | 85.70% |

**Anonymous comments**

1. The health authority need to define who is level 2 or 3 to avoid useless competitions.
2. I agree. Full agreement.
3. This has been done in LRS (name of country concealed for protection of anonymity) and has worked !!

**6.** Coordination of ARDS patient transfer should occur directly between the receiving physician at the level III ICU and the responsible physician, typically an anesthesiologist or intensivist, at the referring level I or II ICU.

| Status | Consensus | Agreement |
| --- | --- | --- |
| Consensus achieved | Yes | 80.30% |

**Anonymous comments**

1. The coordination of the patients transport between ICU in different countries should be directed through directors (head) of the departments. Only then as written.
2. An anesthesiologist or intensivist may not always be available universally, especially in low-resource settings, rural areas, or regions with different ICU staffing models. Pulmonologists, emergency physicians, or general practitioners may handle ICU patients. Direct coordination will definitely help preventing miscommunication that might occur when transfers are arranged only through administrative channels.
3. I agree that communication between the transferring and the receiving physician is ESSENTIAL.
4. A telemedicine may be used, if possible.
5. The receiving primary clinician (e.g. surgeon or other who will be required to perform surgical interventions) should also be part of the referral process to ensure appropriate holistic care once the patient arrives at the level III ICU.

**7.** Transport of ARDS patients to a level III ICU can be organized in two ways: (1) after initial stabilization at the referring center using locally available resources, or (2) by dispatching a mobile expert team from the level III ICU potentially equipped with advanced support (such as V-V ECMO) to stabilize and retrieve the patient.

| Status | Consensus | Agreement |
| --- | --- | --- |
| Consensus not achieved | No | 76.15% |

**Anonymous comments**

1. Mobile expert team might not be feasible due to cost and personnel constrains. Tele ICU should be encouraged to connect experts at hub to peripheral, level 2 centers as spokes to guide initial management.
2. These 2 options are functions of the level of stability of the patient (e.g. severe hypoxemia with hemodynamic instability), the distance between the 2 facilities and how robust and effective the referral system is.
3. Either way is fine.
4. Option 2 will be preferable.
5. Yes, full agreements.
6. Option 2 is ideal, but is unlikely to be funded in our setting. A nationally available guideline/transport protocol will be useful in this instance.
7. both are alternatives depending on patient condition.

**8.** Safe transfer, as part of ARDS management, requires adequate transport vehicles, appropriate medical equipment, a trained team, and complete medical documentation to ensure both clinical and legal continuity.

| Status | Consensus | Agreement |
| --- | --- | --- |
| Consensus achieved | Yes | 84.26% |

**Anonymous comments**

1. Agreement.

**9.** Ethical considerations, in accordance with local laws, should be taken into account when evaluating the risk-benefit balance, burden, and costs of treating and transporting ARDS patients. This involves assessing whether transport is appropriate for patients with a condition that, despite all interventions, is likely to result in a poor outcome based on their current clinical status and prognosis, while considering both the potential benefits of higher-level care and the ethical implications of imposing potentially futile interventions on patients.

| Status | Consensus | Agreement |
| --- | --- | --- |
| Consensus not achieved | No | 78.10% |

**Anonymous comments**

1. Patients with highly morbid conditions or moribund patients in LRS should be excluded when transporting critically ill patients.
2. Refer Point 3 above.
3. It is necessary to set clear indications for the VV ECMO. If there is no such condition, patients will arrive late at the tertiary institution, and the outcome will be the same as if they had not been transported to the tertiary institution. It is necessary to transport the patient in a timely manner.
4. full agreement.
5. Absolutely!

## Domain 2: Diagnostic tools for the identification of ARDS in LRS

**Statements**

**1.** In the absence of gas analyses, ARDS could be diagnosed using the SpO2/FiO2 ratio, in association with bilateral infiltrates demonstrated on chest X-ray or lung ultrasound. However, the usefulness of such a definition remains to be demonstrated and should not routinely replace PaO2/FiO2 ratio.

| Status | Consensus | Agreement |
| --- | --- | --- |
| Consensus achieved | Yes | 80.03% |

**Anonymous comments**

1. We suspect that PaO_2_/FiO_2_ ratio will not be eliminated from future definitions of ARDS. Of note, a standardized level of FiO_2_ and PEEP has never been a condition for defining hypoxemia under MV. We suspect that none of proposed indices of oxygenation for ARDS categorization and prediction of outcome will be useful to make clinical decisions unless assessed or calculated using standardized ventilatory settings. Although the S/F ratio is less invasive there are inaccuracies in SpO_2_ measurements, particularly among patients with darker skin and those in shock and/or with poor distal perfusion. Unfortunately, SpO_2_ is affected by several variables such as changes in temperature, pH, PaCO_2_, concentration of 2,3-diphosphoglycerate and carboxyhemoglobin, and its measurement is influenced by ethnicity, although none of these variables affect PaO_2_. SpO_2_/FiO_2_ ratio contains all the problems of PaO_2_/FiO_2_ ratio, with the added problem that the 95% confidence interval for SpO_2_ vs. SaO_2_ is ± 5% when patient is desaturated, and PaO_2_ values could fluctuate >300 mmHg when SpO_2_ is ≥97%.
2. I think there is growing evidence base supporting SpO2/FiO2 use as a validated surrogate and the clinicians might feel comfortable using it when P/F ratio is not readily available.
3. Any diagnostics are good. Where there is no gas analyzer, the patient has no conditions for treatment.
4. Yes, I agree.
5. Given the current knowledge base, SpO_2_/FiO_2_ could be used as suggested by the Kigali modification. Efforts should be made to better understand possible limitations such as the effect of poor perfusion states and skin color. Ideally, these should be driven by LMIC.
6. Is SpO_2_/FiO_2_ well validated? How accurate is it and how efficient is it as a tool in the management of ARDS bearing in mind factors that can affect readings of SpO_2_. I agree it should not replace PaO_2_/FiO_2._
7. Apply ONLY in the absence of gas analyses.
8. I suggest the recommendation is broken into 2, separating the role of S/F ratio from the usefulness comparison to P/F statement.

**2.** The use of lung ultrasound (LU) by a trained physician should be considered a valuable diagnostic tool for ARDS in accordance with the global definition, particularly in low-resource settings (LRS) where access to chest X-rays may be limited. LU findings, such as bilateral lung consolidations or B-lines (comet-tail artifacts), are indicative of interstitial pulmonary edema and align with other diagnostic criteria for ARDS.

| Status | Consensus | Agreement |
| --- | --- | --- |
| Consensus not achieved | No | 76.22% |

**Anonymous comments**

1. Lung ultrasound is not always available in LRS hospitals.
2. The inclusion of the chest radiograph criterion remains a question given its moderate-to-poor reliability and limited availability in some settings. Other approaches to radiography in ARDS include computed tomography (CT) scanning (that are uncommon in LRS) and lung ultrasound (US). The use of US requires operating characteristics less known and requires training in image acquisition to meet the definition criteria.
3. I can hardly imagine not having an XR and even a CT scan in my country.
4. I would not mention the global definition, which is not the official definition of ARDS.
5. This is really difficult to do. Where X ray is limited, it is not a place to treat patients with pneumonia, let alone ARDS.
6. Mobile chest Xray, blood acid-base balance and blood gas analysis are all basic investigation in all ICUs in European countries. You need it all for right diagnosis according Berlin criteria.
7. Should be trained to differentiate other causes of B lines with clinical correlation. Also, it should incorporate ECHO training. Tele ICU could also help with diagnosing B lines.
8. With cardiac function assessment.
9. POCUS is the future of Critical Care Medicine. The use of LU should be encouraged in our ICUs.

**3.** The use of lung ultrasound for the diagnosis and monitoring of ARDS in low-resource settings remains below expectations and should be further encouraged and standardized.

| Status | Consensus | Agreement |
| --- | --- | --- |
| Consensus not achieved | No | 75.09% |

**Anonymous comments**

1. I strongly agree with this statement. In my opinion, even greater efforts should be made to promote the adoption of lung ultrasound through the development of standardized protocols, structured training programs, and its integration into evidence-based guidelines. This approach would help ensure consistent and effective use of this valuable tool, particularly in low-resource settings where alternatives may be limited.
2. Agreement.
3. Absolutely!
4. Doctors are not routinely trained in its use and interpretation.
5. The use of ultrasound is certainly a necessary part of the knowledge of a modern doctor.
6. Understanding its current use in a LRS should be a priority for it to be optimized.
4. Lung ultrasound in addition to diagnosis can guide targeted volume management of patients with ARDS in addition to conservative fluid balance and lung protective strategies.

| Status | Consensus | Agreement |
| --- | --- | --- |
| Consensus not achieved | No | 73.69% |

**Anonymous comments**

1. The use of US requires operating characteristics less known in LRS and requires training in image acquisition to meet the definition criteria.
2. No use of US in ARDS diagnosis or management.
3. lung and heart ultrasound.
4. I agree that lung ultrasound can guide volume management, but it is only one piece of the puzzle. Other metrics and tools should be taken into account and combined with lung ultrasound findings while guiding targeted volume management.
5. Agreement.
6. There is data estimating EVLW and lung ultrasound.
7. The problem is to have trained people, if you are thinking in a place that doesn't have a CT scan, hardly will have a trained person in Lung ultrasound.

## Domain 3: treatment – respiratory support

**Statements**

**1.** Non-invasive ventilation (NIV) and high-flow nasal cannula (HFNC) should be considered first-line therapy for patients with mild/moderate ARDS with stable hemodynamics and where there are no contraindications. This approach is particularly practical and valuable in LRS, where it can be implemented with minimal monitoring and does not require highly specialized personnel.

| Status | Consensus | Agreement |
| --- | --- | --- |
| Consensus not achieved | No | 79.35% |

**Anonymous comments**

1. I would consider this recommendation if broken into 2 parts separating the core statement from the second part that seems to disregard the significant risk of NIV by less qualified or experienced providers.
2. The use of HFNO has increased in the past decade, particularly during the COVID-19 pandemic. Proponents suggest that the ARDS definition should be modified to allow patients on HFNO to be eligible for the oxygenation criteria even though they are not ventilated with PEEP 5 cmH_2_O (as required by the Berlin definition). This approach has not been validated since in many cases those patients had simply hypoxemic ARF. Also, the exact FiO_2_ is difficult, if not impossible, to be determined in patients on NIV or HFNO. In addition, many patients require intubation and mechanical ventilation IMMEDIATELY.
3. NIV can be more complex to manage and perform adequately than invasive mechanical ventilation.
4. Not sure that NIV in ARDS pts does not require trained personnel.
5. However, this is practice is monitoring intensive and health care workers should be present constantly monitoring to escalate care as soon as deterioration is recognized.
6. I strongly agree with this statement, however I would highlight the need for careful patient selection and close monitoring to detect failing cases early in the statement, since delayed intubation may worsen outcomes.
7. Full agreement.
8. In Selected centers.
9. We need to provide an estimated timeframe for response assessment for NIV and HFNC. A suggested approach to non-response will provide a safety net, avoiding delayed invasive mechanical ventilation and the associated harms of delay.
2. Patients who fail any form of non-invasive ventilation should, along with their closest family members, be informed about the risks, costs, burdens, and alternatives to intubation, including the potential need for prolonged mechanical ventilation and tracheostomy. Based on this information, and in accordance with local laws and regulations, they should be given the opportunity to decide whether to forgo such forms of life-sustaining treatment.

| Status | Consensus | Agreement |
| --- | --- | --- |
| Consensus not achieved | No | 78.19% |

**Anonymous comments**

1. This can make it difficult to treat the patient. It can delay intubation and the use of mechanical ventilation. It is not easy to obtain the consent of the patient and family members. Sometimes the patient is not conscious, the family does not come, etc.
2. I agree with the first part of statement. But, according to the second part of the statement, in many LRS, such as Bosnia and Hercegovina, patient or relatives do not have right do decide to e.g. stop prolonged ventilation, or stop life-sustaining treatment.
3. The use of HFNO has increased in the past decade, particularly during the COVID-19 pandemic. Proponents suggest that the ARDS definition should be modified to allow patients on HFNO to be eligible for the oxygenation criterion even though they are not ventilated with PEEP 5 cmH2O (as required by the Berlin definition). This approach has not been validated since in many cases those patients had simply hypoxemic ARF. Also, the exact FiO2 is difficult, if not impossible, to be determined in patients on NIV or HFNO.
4. Yes I agree.
3. Noninvasive ventilation (NIV/HFNC) in LRS could be used for patients with severe ARDS for short time to provide valuable initial time to stabilize the patient, ensure adequate pre-oxygenation, and create conditions for safe intubation. However, recognition of NIV/HFNC failure should be the priority to avoid harmful delays in definitive airway management.

| Status | Consensus | Agreement |
| --- | --- | --- |
| Consensus not achieved | No | 76.80% |

**Anonymous comments**

1. The use of HFNO has increased in the past decade, particularly during the COVID-19 pandemic. Proponents suggest that the ARDS definition should be modified to allow patients on HFNO to be eligible for the oxygenation criteria even though they are not ventilated with PEEP 5 cmH2O (as required by the Berlin definition). This approach has not been validated since in many cases those patients had simply hypoxemic ARF. Also, the exact FiO2 is difficult, if not impossible, to be determined in patients on NIV or HFNO.
2. Based on my experience in low-resource settings, teams often overlook patients receiving NIV or HFNC support and underestimate the importance of close monitoring to promptly identify the need for intubation. Maybe, incorporating recommendations for a maximum duration of NIV/HFNC use and establishing clear monitoring protocols to ensure timely escalation of care would be valuable additions to this statement.
3. Maybe provide some indications on tools or score to use to predict NIV/HFNC failure?
4. Time interval for 1-2 hours to test effectiveness of NIV /HFNC.
5. It is extremely important to recognize the need for intubation and mechanical ventilation in time.

**4.** Prone positioning may be utilized in awake ARDS patients who received non-invasive support (NIV/HFNC) to improve oxygenation and enhance lung-protective strategies.

| Status | Consensus | Agreement |
| --- | --- | --- |
| Consensus not achieved | No | 74.59% |

**Anonymous comments**

1. According to the severity of disease, participation of the patient, agitation and skills of the team
2. This is pending further research confirming usefulness outside COVID-19
3. Semi prone is OK.
4. If the expertise, safety and staffing requirements are available should be added to the recommendations for me to consider it
5. very cheap and effective treatment should be done.
6. I agree with this statement, but patient tolerance is one of the key aspects of prone positioning and this should be closely monitored. And it is not a substitute for escalation to invasive mechanical ventilation when needed.
7. Yes, but with significant risk for patients.
8. Should this be only in awake patients? could also be applicable in intubated patients

**5.** The decision to intubate ARDS patients should be based on clinical response to non-invasive support and the likelihood of benefit, taking into account ethical considerations and local legal frameworks.

| Status | Consensus | Agreement |
| --- | --- | --- |
| Consensus achieved | Yes | 80.45% |

**Anonymous comments**

1. I think that the decision about intubation and mechanical ventilation of a patient with ARDS should not be related to the probability of benefit, ethical considerations and local legal frameworks.
2. Many patients require intubation and MV immediately.
3. Full agreement.
6. In LRS, where experience with various ventilation modes may be limited, the mode most familiar to the physician and medical team should be used to ensure safe and effective care.

| Status | Consensus | Agreement |
| --- | --- | --- |
| Consensus achieved | Yes | 80.00% |

**Anonymous comments**

1. There is disconnection between the conceptual model of ARDS—a specific type of inflammation and the lack of measures of inflammation in ARDS definitions. Diagnostic accuracy in ARDS is challenging without a universal reference standard. Future work in refining the ARDS definition should carefully consider other facets of validity. Clinicians may wish to use the broader umbrella syndrome of acute hypoxemic respiratory failure when deciding to implement certain therapeutic strategies, particularly those that are not directed against specific ARDS mechanisms. However, many hypoxemic patients who improve after 24 h of standard intensive care do not have ARDS. We do not cure ARDS in 24 h.
2. They could also be guided by experts by utilizing Tele ICU.
3. I fully agree and support this statement. On the other hand, I sometimes wonder if this might become a barrier in front of ongoing training and capacity building to expand mechanical ventilation skills over time. Because a small subset of ARDS cases may benefit from advanced (fancy!) or less commonly used ventilation modes. When feasible, consultation with more experienced centers should be encouraged and this may help improve outcomes and support skill development of the local physicians.
4. It would be useful to survey this to ensure appropriate educational programs are in place.
7. In intubated and mechanically ventilated ARDS patients, low tidal volume ventilation (6-8 mL/kg of predicted body weight) should be used as a safe and effective lung-protective strategy."

| Status | Consensus | Agreement |
| --- | --- | --- |
| Consensus achieved | Yes | 82.38% |

**Anonymous comments**

1. I prefer to use 4 - 6 ml/kg.
2. I am of the opinion that where there is a mechanical ventilation device, intensivists and anesthesiologists need to be better trained in its use.
3. 4 -6ml/kg
4. Current standard (conventional) MV include low tidal ventilation for all patients with acute respiratory failure. All mammals are scaled similarly and the Vy is about 6.2 ml/kg “ideal“ body weigh.
5. Agree
8. Simple nomograms showing predicted body weight and corresponding tidal volume settings for both men and women should be prominently displayed on each ventilator in LRS. This practice would significantly reduce the risk of errors and improve the implementation of lung-protective ventilation strategies."

| Status | Consensus | Agreement |
| --- | --- | --- |
| Consensus not achieved | No | 78.32% |

**Anonymous comments**

1. Okay, that's the basics. But where are the FiO2, PEEP, the frequency, plateau pressure,.
2. not only in LRS, but in all the world...
3. In fact, I strongly recommend to all MV industries to include the height and the sex in all ventilators, and develop an algorithm that calculate the recommended VT. Also, I recommend the design of alarms in ICU patients that are ventilated, showing the total volumes applied.
4. Yes, good suggestion.

In mechanically ventilated ARDS patients, the initial PEEP should be set at 10cm H2O, and later titrated according to the patients clinical status (oxygenation and hemodynamic status) so that the plateau pressure is less than 30 cm H2O). It should be kept in mind that in patients with left ventricular failure or right heart failure, PEEP levels may need to be adjusted upward or downward based on clinical response and tolerance."

| Status | Consensus | Agreement |
| --- | --- | --- |
| Consensus not achieved | No | 75.20% |

**Anonymous comments**

1. I would give a range: 5 to 10 cm of water. Higher PEEP should be considered in specific settings. Maybe avoid lower values than 5
2. This would be very frivolous. Operating a mechanical ventilation machine is a serious science. The parameters change from hour to hour. Where there is no trained staff, patients with ARDS should not be treated.
3. I am not sure about the strong evidence that supports initial PEEP should be 10 cmH2O.
4. The requirement for PEEP should not be removed regardless of oxygen delivery device used. PEEP is a routine approach even in the operating rooms. If you removed PEEP, patients labeled as ARDS, do not have ARDS. We need to standardize the level of PEEP and the level of FiO_2_ to calculate PaO_2_/FiO_2_. A standardized level of FiO_2_ and PEEP has never been a condition for defining hypoxemia under MV. We suspect that none of proposed indices of oxygenation for ARDS categorization and prediction of outcome will be useful to make clinical decisions unless assessed or calculated using standardized ventilatory settings. In patients fulfilling ARDS criteria, assessment at 24 h on PEEP 10 cmH_2_O with FiO_2_ 0.5 for 30 min caused PaO_2_/FiO_2_ ratio to increase, such that more than a third of patients no longer met ARDS criteria.
5. This appears to be a practical approach.
6. Full agreement.
9. All intubated ARDS patients should have plateau pressure measured as part of their ventilatory management to guide lung-protective strategies. An inspiratory hold of 2 seconds should be performed to obtain accurate measurements (every 6-8 hours or after any major ventilator adjustment).

| Status | Consensus | Agreement |
| --- | --- | --- |
| Consensus achieved | Yes | 80.93% |

**Anonymous comments**

1. no need to hold breath for this.
2. I'm disoriented. I don't know who is treating the patient, a nurse, a doctor, or someone else. Maybe I take mechanical ventilation too seriously.
3. 0.5-1 seconds is enough.
4. Short paralysis of the patient?
5. Good idea.
6. Lung protective ventilation includes the use of low VT (4-8 ml/kg PBW), a plateau pressure <29 cmH2O, and moderate levels of PEEP (8-12 cmH2O).
10. A short recruitment maneuver lasting less than 40 seconds should be considered to improve oxygenation in patients with severe hypoxemia (requiring FiO_2_ greater than 80%).

| Status | Consensus | Agreement |
| --- | --- | --- |
| Consensus not achieved | No | 68.14% |

**Anonymous comments**

1. It depends on the etiology of hypoxemia. For instance, recruitment will never be conducted in severe hypoxemia in PE.
2. I do not prefer the short-term RM as it's effect is short termed and you should do recruitment only for the recruitable lung. So you need a tool to tell you that the lung is recruitable or not like the PV tool. If recruitable we should implement the long-term RM as optimized PEEP or proning. If I applied short term RM then stopped then the patient will be hypoxic following stopping it. Additionally, the hemodynamic effects might be compromised as well. If the lung is not recruitable this might lead to VILI and pneumothorax which is harmful.
3. this depends on the patient, hemodynamics and suitability for prone position.
4. No sense. High risk.
5. If the expertise exists.
6. Check on the ventilator if the lungs are recruitable.
7. The side effects and harmful effects of resulting hemodynamic instability following RM should be monitored.
8. I agree with this statement. However, since some patients may deteriorate due to hypotension or worsening compliance, careful patient selection, close monitoring, timely de-escalation, and an individualized titration approach should be emphasized when considering recruitment maneuvers.
9. Collapsed or atelectatic areas of the lung can be re-expanded by the application of a brief period of high transpulmonary pressure followed by the application of adequate levels of PEEP to maintain the new aerated regions open.
10. And this is a very serious procedure. Maybe do a “little“ recruitment.
11. Prolonged recruitment maneuvers lasting more than 60 seconds should be avoided to minimize the risk of ventilator-induced lung injury and hemodynamic instability."

| Status | Consensus | Agreement |
| --- | --- | --- |
| Consensus not achieved | No | 77.24% |

**Anonymous comments**

1. Concerns with the ART trial design, methodology, data analyses, and results—in addition to possible major differences with health care systems—provide solid arguments to question the results of ART trial.
2. Yes, You are right.
3. It can be used in intensive care units, but routine use is not recommended.
4. Despite doing this practice but the same concern. If I am planning to do.
5. The mechanical ventilation device should be located where trained personnel are located.
6. In my opinion it should not be done.
12. Intubated patients with moderate-to-severe ARDS who have severe hypoxemia (with P/F ratio ≤150 or the equivalent S/F ratio ≤200), should be placed in the prone position to improve oxygenation."

| Status | Consensus | Agreement |
| --- | --- | --- |
| Consensus not achieved | No | 78.35% |

**Anonymous comments**

1. Expertise, staffing and safety considerations are important.
2. After few hours of stabilization?
3. In principle, I support this recommendation but would say “prone positioning should be considered unless there are important contraindications.
4. To improve mortality than oxygenation.
5. According to the PROSEVA trial, it seems that candidate patients for beneficial effects of prone ventilation are those with PaO2/FiO20.5 and moderate level of PEEP. However, the PROSEVA trail MUST be validated because it reported the LOWEST 28-day mortality rate EVER REPORTED!!!!!!!!!!. I do not trust the Use of SpO2/FiO2 instead of PaO2/FiO2 for the reason explained above in the P/F section.
6. Despite.
7. Yes, full agreement.
8. Contraindications (e.g. hemodynamic instability) to prone positioning should be considered.
13. Intubated patients with moderate-to-severe ARDS should be kept in the prone position for 16 hours daily."

| Status | Consensus | Agreement |
| --- | --- | --- |
| Consensus not achieved | No | 74.20% |

**Anonymous comments**

1. Same comments as 13.
2. Technical problems may cause some harm of prone. 16 hours prone can be done in some limited places.
3. If possible, and if it's the only option.
4. It depends on the proning was successful and improving the ABG. If PF ratio improved following the proning we should continue. But if not improved there is no benefit to continue the proning.
5. At least >12 hours, on lung protective ventilation. In NIV, we do not the time to stop progression or decrease mortality.
6. I agree with this statement, but some centers may adjust duration based on individual patient tolerance, staffing, and resources especially in LRS. Optimal duration time is still a topic of ongoing research.
7. Taking in consideration the prevention of skin ulcers.
8. Sometimes consider extended lying position for 24 hours due to lack of medical staff in the LRS.
9. Optimal 20-22 hours.
14. The prone position requires trained teams to ensure its safe implementation, and staff education on this maneuver is essential.

| Status | Consensus | Agreement |
| --- | --- | --- |
| Consensus achieved | Yes | 84.60% |

**Anonymous comments**

1. Trained teams and enough personnel in the LRS.
2. Agreement.
3. Prone positioning needs more staff that are trained. This could be a challenge in LRS where shortage of personnel is a major issue.
15. In patients with severe ARDS, prone positioning should be applied at least four times during the first 72 hours, with each session lasting 16 to 18 hours. In patients requiring FiO_2_ of 0.6 or lower in the supine position, prone positioning may no longer be necessary.

| Status | Consensus | Agreement |
| --- | --- | --- |
| Consensus not achieved | No | 73.25% |

**Anonymous comments**

1. Any evidence to support these numbers?
2. RCT are needed to recommend that particular protocol.
3. This sounds very categorical. I would say that the response to prone positioning should aways be assessed and the intervention should be repeated as often as the patient shows benefit.
4. If there is severe ARDS, but patient maintains good saturation at FiO_2_ of less than or equal to 0.6, I will rather do more of chest physio.
5. I agree that a FiO_2_ level of 0.6 is reasonable, but clinical judgement often guides this decision.
6. Yes, You are right.
7. Could we provide guidance around repeated supination of proned patients who are responding well to this therapy and have no indication to be placed in the supine position during this initial period.
16. The new global definition of ARDS (2024) expands the 2012 Berlin criteria to improve applicability across diverse clinical and resource settings. Key updates include:
    - Diagnostic Categories (nonintubated ARDS - Patients on high-flow nasal oxygen (HFNO ≥30 L/min) or noninvasive ventilation (NIV) meeting oxygenation criteria, Intubated ARDS - Aligns with Berlin criteria but incorporates broader imaging options. Resource-limited settings: Adopts the Kigali modification, allowing diagnosis without advanced respiratory support or arterial blood gas (ABG) measurements.
    - Oxygenation Criteria - accepts SpO_2_/FiO_2_ less or equal to 315 (if SpO_2_ is less or equal to 97%) alongside PaO_2_/FiO_2_ less or equal to 300.
    - Imaging - Adds lung ultrasound (bilateral B-lines/consolidation) to chest radiograph/CT.
      - Timing - Retains the Berlin requirement: acute onset (within 1 week of known insult) This new global consensus definition demonstrate greater applicability in LRS then the Berlin definition of ARDS.

| Status | Consensus | Agreement |
| --- | --- | --- |
| Consensus not achieved | No | 76.62% |

**Anonymous comments**

1. The so-called “global definition“ of ARDS is not global; it has not been supported by any professional society. In addition, patients on NIV of HFNO could improve dramatically after a RM, after initial resuscitation, and stabilization. If ARDS is an “intense inflammatory condition of the lung“ cannot last <24 hours. There is disconnection between the conceptual model of ARDS—a specific type of inflammation and the lack of measures of inflammation in ARDS definitions. Diagnostic accuracy in ARDS is challenging without a universal reference standard. Future work in refining the ARDS definition should carefully consider other facets of validity. Clinicians may wish to use the broader umbrella syndrome of acute hypoxemic respiratory failure when deciding to implement certain therapeutic strategies, particularly those that are not directed against specific ARDS mechanisms. However, many hypoxemic patients who improve after 24 h of standard intensive care do not have ARDS. We do not cure ARDS in 24h.
2. this definition remains to be validated and agreed by societies.
3. Doubtful.
4. Some LRS don't have Ultrasound machines.
5. Most hospitals I know do not have a HFNC.
6. Both definitions of ARDS have their place and role, but - Imaging: LU is operator dependent, and has some limitation such is obesity- SpO_2_/FiO_2_ ratio: is not so reliable as PaO_2_/FiO_2_.

## Domain 4: other supportive therapies

**Statements**

1. Based on the knowledge gained during the COVID-19 pandemic, corticosteroids, such as dexamethasone (at doses less than 20 mg) or equivalent doses of other corticosteroids, should be considered in the management of ARDS, provided there are no contraindications.

| Status | Consensus | Agreement |
| --- | --- | --- |
| Consensus not achieved | No | 72.04% |

**Anonymous comments**

1. No strong evidence in other ARDS.
2. Only in moderate-severe and non-influenza.
3. early?
4. According to the DEXA-ARDS trial, and several metanalysis, this recommendation is strong.
5. Yes, for limited time of exudative phase of ARDS (early phase 5-7 days).
6. Some concerns regarding Flu ARDS regarding steroids but I prefer to give the steroids if sever ARDS.

**2.** The duration of corticosteroid therapy in ARDS should not exceed 10 days, and tapering is not necessary if corticosteroids are administered for 10 days or less.

| Status | Consensus | Agreement |
| --- | --- | --- |
| Consensus not achieved | No | 74.42% |

**Anonymous comments**

1. No evidence.
2. According to the DEXA-ARDS trial (Lancet Respir Med 2020) is correct.
3. Around one week is sufficient, with de-escalation.

**3.** The use of neuromuscular blocking agents (NMBA) should be considered in deeply sedated patients during the early course of ARDS, ideally within the first 48 hours and for a short duration.

| Status | Consensus | Agreement |
| --- | --- | --- |
| Consensus not achieved | No | 78.51% |

**Anonymous comments**

1. I would only consider NMBA if there is ventilatory dyssynchrony despite deep sedaton.
2. Requires training.
3. and moderate severe.
4. The ACURASYS trial showed that in patients with moderate-to-severe ARDS, a strategy of 48 hours of deep sedation with muscle paralysis induced by an intravenous infusion of cisatracurium resulted in a lower incidence of barotrauma and higher adjusted overall survival at 90 days than deep sedation alone. These results were unexpected, since the intervention was performed only for the first 2 days, yet the Kaplan–Meier survival curves were virtually superimposable for about 18 days before they separated.
5. If necessary.
6. This only has evidence for cisatracurium and extending this to all NMBA leads to overuse of NMBA and increased incidence of CINM, and increased LOS MV and LOS ICU.
7. Agreement.

**4.** The use of neuromuscular blockade in the form of continuous infusion for the treatment of ARDS should be limited to 48 hours from intubation. After that, if muscle relaxation is still needed, intermittent administration should be preferred to reduce lung injury caused by patient-ventilator asynchronies.

| Status | Consensus | Agreement |
| --- | --- | --- |
| Consensus not achieved | No | 70.74% |

**Anonymous comments**

1. No evidence for this.
2. Dependence on the clinical picture, the results of gas analyses.
3. I believe this statement is accurate, but in situations where there is a shortage of bedside healthcare personnel, it is sometimes impossible to ensure timely intermittent administration of neuromuscular blockade.
4. The ROSE trial was performed to reexamine the benefits of cisatracurium-induced paralysis in patients early after the onset of ARDS. Patients with moderate-to-severe ARDS were assigned either to a 48-hour continuous infusion of cisatracurium with deep sedation or to a usual-care approach with light sedation and without routine NMBA blockade. The results were markedly different from those of the ACURASYS trial.
5. Also, for patients with increased respiratory effort and this should be objectively measured in some way.
6. Agree.

**5.** The use of neuromuscular blocking agents plays a significant role in the management of ARDS and should be considered in cases of patient/ventilator asynchrony, particularly during the initial hours of mechanical ventilation, until optimal ventilator settings are achieved.

| Status | Consensus | Agreement |
| --- | --- | --- |
| Consensus not achieved | No | 69.60% |

**Anonymous comments**

1. Dependence on the clinical picture, the results of gas analyses.
2. Not necessary. This shall be used very rarely and cautiously. Adequate sedation with Fentanyl and Propofol with adequate doses can manage more than 90% of patients with Asynchrony or when proning.
3. This has not yet been proven unequivocally, especially in LRS where training may be inadequate
4. I recommend that NMBA not be used routinely in patients with moderate-to-severe ARDS. There is a rationale to consider neuromuscular blocking agents in any patient with ARDS who, despite carefully implemented ventilatory and sedation strategies, has a ventilatory pattern that confers a predisposition to VILI. NMBA may also be considered in patients with increased respiratory drive that could generate potentially injurious transpulmonary pressure swings. NMBA can sometimes be used, when physiologically and clinically indicated.

**6**. The use of neuromuscular blocking agents is particularly important in LRS during the initial treatment of ARDS in level I and II intensive care units, as well as during transport to level III ICUs, where a greater number of experts can optimize mechanical ventilation without the use of neuromuscular blockade.

| Status | Consensus | Agreement |
| --- | --- | --- |
| Consensus not achieved | No | 69.78% |

**Anonymous comments**

1. It is irrespective to the LRS. I do not agree.
2. question is not clear.

**7.** The monitoring of neuromuscular blockade during mechanical ventilation of patients with ARDS in LRS should rely on clinical examination (e.g., presence of signs of patient-ventilator asynchrony).

| Status | Consensus | Agreement |
| --- | --- | --- |
| Consensus not achieved | No | 61.74% |

**Anonymous comments**

1. TOF can be used.
2. TOF and NM Monitoring shall be used.
3. Even in LRS there can be anesthesia rooms with TOF availability.
4. All this requires well-trained personnel.
5. While I agree with this, basic waveform interpretation and regular sedation assessment should also be encouraged.
6. And the objective measurement of respiratory effort using at least expiratory occlusion pressure.

**8**. Conservative fluid management and achieving a negative fluid balance have demonstrated an advantage over liberal fluid administration in the treatment of stable ARDS. Fluid balance should be closely monitored, aiming for either a neutral or negative balance. If necessary, the use of diuretics or available dialysis procedures can be considered to achieve this goal.

| Status | Consensus | Agreement |
| --- | --- | --- |
| Consensus achieved | Yes | 81.95% |

**Anonymous comments**

1. In patients without signs of hypovolemia and adequate cardiac output and tissue perfusion!!!!
2. All this requires well-trained personnel. It is necessary to urgently transport the patient to a higher center.

**9**. In LRS, the use of inhaled vasodilators is generally not recommended due to limited access to monitoring equipment and the potential for adverse effects."

| Status | Consensus | Agreement |
| --- | --- | --- |
| Consensus not achieved | No | 69.22% |

**Anonymous comments**

1. Could still be used as live-saving intervention to transport the patient to level III centers.
2. Should be limited to centers with the highest levels of experience, personnel and equipment.

**10**. The use of V-V ECMO in the treatment of ARDS in LRS should be limited to centers with the highest levels of experience and personnel, with the prerequisite that there is the possibility of adequate and safe transport of these patients (e.g., by special ambulance, helicopter, etc.).

| Status | Consensus | Agreement |
| --- | --- | --- |
| Consensus achieved | Yes | 83.53% |

**Anonymous comments**

1. No comments on this question

**11**. Early tracheostomy, particularly percutaneous, may provide clinical benefits for patients with ARDS being treated in LRS, especially when performed within the first 7 to 10 days and in patients with ventilator parameters including FiO2 below 60% and PEEP below 8.

| Status | Consensus | Agreement |
| --- | --- | --- |
| Consensus not achieved | No | 63.12% |

**Anonymous comments**

1. No evidence.
2. Expertise in percutaneous tracheostomy may not be available in LRS. Also, extubation and commencement of NIV may be considered if indicated otherwise tracheostomy is good.
3. this is variable and sometimes waiting up to 14 days may be able to save tracheostomies.

## Domain 5: Sedation Strategies

**Statements**

**1**. Use the most readily available short -acting sedative (ideally propofol) with basic monitoring (e.g., observation of movement, facial expressions). Assess sedation clinically: monitor for spontaneous movements, eye opening, facial grimacing, and breathing against the ventilator, using monitoring scales with which clinicians in low-resource settings are familiar (e.g., RASS, BPS, etc.).

| Status | Consensus | Agreement |
| --- | --- | --- |
| Consensus achieved | Yes | 82.98% |

**Anonymous comments**

1. Include also BPS score
2. Is propofol long acting or mistake?
3. Propofol is short acting, not long acting.
4. I agree with the statement, however assuming the availability of propofol might not always be true. Also in combination with short-acting opioids (like fentanyl) might increase the quality of sedation and decrease the dose of propofol.
5. Centers and countries should develop and validate such score (for example: CAM ICU score for delirium assessment) translated to language that the patient understands. Visual cards can also be used
6. Correction a long-active sedative with short-acting sedative (propofol)
7. In LRS it is not possible. Too much is expected from LRS. In my opinion, LRS does not have the ability to treat ARDS.

**2**. Avoid oversedation whenever possible by using sedation depth monitoring and daily wake-up tests, if ventilator parameters allow, as excessive sedation can increase the risk of prolonged mechanical ventilation and delirium.

| Status |  | Consensus | Agreement |
| --- | --- | --- | --- |
| Consensus achieved |  | Yes | 80.14% |

**Anonymous comments**

1. Not clear if we need to recommend that in severe ARDS. As awakening can increase asynchrony. and derecruited patient increase oxygen consumption…In the weaning phase it makes sense.
2. Agreement.
3. Unless patient is paralyzed.
4. Too much is expected from LRS. In my opinion, LRS does not have the ability to treat ARDS.
5. Avoid benzodiazepines if feasible.

**3**. Prolonged ventilation increases the risk of withdrawal syndrome when sedatives are interrupted, necessitating careful management and gradual weaning to reduce the likelihood of complications.

| Status | Consensus | Agreement |
| --- | --- | --- |
| Consensus not achieved | No | 73.82% |

**Anonymous comments**

1. Not clear statement. Withdrawal syndrome is related to prolonged sedation more than ventilation.
2. this is true to Dexmedetomidine and possibly benzodiazepines, not for propofol.
3. Prolonged sedation? not ventilation.
4. However there are other factors as well like nutrition and physiotherapy that are often neglected.

**Domain 6: Weaning**

**Statements**

**1**. Spontaneous breathing trials (SBT) should be the primary method for weaning from MV in LRS, particularly using simple techniques such as the T-piece trial or pressure support ventilation when advanced ventilator modes are unavailable. In general, prerequisites for the SBT included a partial reversal of the underlying cause of ARDS, a PaO2/FiO2 ≥200 mmHg with PEEP ≤10 cmH2O and FiO2 lower or equal to 0.4, no vasopressors, continuous sedation minimized, and ability to cough during tracheal aspirations. The duration of the SBT should be at least 30 min and no longer than 120 min.

| Status | Consensus | Agreement |
| --- | --- | --- |
| Consensus not achieved | No | 76.61% |

**Anonymous comments**

1. unclear whether this regards just letting the patient breathe spontaneously on ventilator in a mode. such as ASB, or a breathing trial with T-piece and oxygen. I don't agree with breathing trials on just T-piece and if done, no more than 20-30 min. We test these kinds of patients with high flow connected to ET tube or tracheostomy and wean from mechanical ventilation to HFNO almost always.
2. Those are very strict criteria for SBT.
3. Absence of delirium, tachycardia also can be considered.
4. FiO2 <50% is good enough.

**2**. Weaning should not be attempted until the patient’s oxygenation is stable with FiO2 ≤ 0.6 and PEEP ≤10 cm H2O, and the patient shows no signs of hemodynamic instability or excessive sedation.

| Status | Consensus | Agreement |
| --- | --- | --- |
| Consensus not achieved | No | 70.75% |

**Anonymous comments**

1. Weaning of MV could be started when the attending physician considered it clinically appropriate. In general, prerequisites for the SBT include a partial reversal of the underlying cause of AHRF, a PaO_2_/FiO_2_ >200 mmHg with PEEP<10 cmH_2_O and FiO_2_≤0.4, no vasopressors, continuous sedation minimized, and ability to cough during tracheal aspirations. Spontaneous ventilation should be tested with a T-piece or with pressure support at 8 cmH2O. The duration of the SBT must be at least 30 min and no longer than 120 min.
2. We do not attempt wean before FiO2 is 40% or bellow and PEEP 7 or bellow, and FiO2 is always weaned before PEEP.
3. It is OK to try “some“ weaning, such as changing to more spontaneous mode.
4. Weaning shall be tried every day despite of Fio2 or PEEP.

**3**. Close monitoring of oxygenation (SpO2, PaO2/FiO2), ventilation (pH, PaCO2), and signs of increased work of breathing is crucial during the weaning process to detect failure early and prevent complications.

| Status | Consensus | Agreement |
| --- | --- | --- |
| Consensus achieved | Yes | 80.14% |

**Anonymous comments**

1. I find ultrasound scoring also to be useful.
2. Agreement.

**4**. Since the rate of reintubation after extubation for all indications is estimated at about 20%, patients at high risk for reintubation (≥65 years of age, hypercapnic (PaCO2 ≥45 mmHg after extubation), or ineffective cough ad excessive secretions, with one or more weaning failures, with more than one comorbid condition, with upper airway obstruction, or with APACHE II score ≥12 on the day of extubation], non-invasive ventilatory support (HFNC or CPAP/PSV) for 24 to 48 hours is indicated until stable or requiring reintubation.

| Status | Consensus | Agreement |
| --- | --- | --- |
| Consensus not achieved | No | 70.39% |

**Anonymous comments**

1. Since the rate of reintubation after extubation for all indications is estimated at about 20% (*Kacmarek RM, Respir Care 2019*), patients at high risk for reintubation [>65 years of age, hypercapnic (PaCO2 >45 mmHg after extubation), or ineffective cough ad excessive secretions, with &#8805;1 weaning failure, with more than one comorbid condition, with upper airway obstruction, or with APACHE II score >12 on the day of extubation], non-invasive ventilatory support for 24 to 48 hours is indicated until stable or requiring reintubation.
2. The recommendation is difficult to understand. The statement lacks clarity and is strenuous to read.
3. Could be used/indicated Should be reassessed closely. The timing of 24 to 48 h seems complicate factor to use.
4. Not really if there are secretions, if there are secretions one can try high flow or keep her intubated

**Domain 7: Nutrition**

**Statements**

**1**. In hemodynamically stable ARDS patients, EN should be started within 24–48 hours of ICU admission. Initiation should be delayed in the presence of uncontrolled shock, life-threatening respiratory or metabolic derangements, active GI bleeding, or high gastric aspirate (>500 mL/6 h). EN is contraindicated in bowel ischemia, obstruction, abdominal compartment syndrome, or high-output fistula without distal access. An individualized approach should guide timing based on clinical stability and risk.

| Status | Consensus | Agreement |
| --- | --- | --- |
| Consensus not achieved | No | 73.83% |

**Anonymous comments**

1. Initiation shall not be delayed.
2. Initial dose is important - low dose better (6-10 kcal/kg/day)?
3. “Trophic“ (small amount) can be done without checking the aspirate. In general, there is no need for full feeding during the first week.
4. Gastric aspirate of 250mls every 4 hours.
5. assessment for risk of malnourishment should be done. All patients should be considered to be at risk for malnourishment. they may also present with vitamin deficiencies which would need replacement/ supplementation.
6. Good recommendation but needs to be simplified for clarity.

**2**. Enteral nutrition via nasogastric tube should be the standard approach, with continuous feeding preferred over bolus. Enteral nutrition is preferred over parenteral nutrition (PN), which should be considered only if enteral nutrition is contraindicated or not tolerated after 3–7 days. In LRS, gravity-assisted continuous or bolus feeding may be used, with volume adjusted according to patient tolerance.

| Status | Consensus | Agreement |
| --- | --- | --- |
| Consensus not achieved | No | 76.17% |

**Anonymous comments**

1. Continuous feeding may not be feasible in LRS as feeding pump is not available in this setting.
2. And regular monitor (gastric residual volume, abdominal distension, diarrhea, vomiting) should be done.

**3**. In LRS, where indirect calorimetry or other precise assessment tools are unavailable, nutritional needs in ARDS patients can be estimated at 20–25 kcal/kg/day for energy and 1.2–1.5 g/kg/day for protein. However, these targets should be approached gradually, starting at approximately 50–70% of estimated needs and increasing by 10–15% per day, aiming to reach full requirements by days 5 to 7 of ICU stay, as tolerated.

| Status | Consensus | Agreement |
| --- | --- | --- |
| Consensus not achieved | No | 73.91% |

**Anonymous comments**

1. Nutrition in LRS depends on availability and costs. In case od hospital-prepared food (and the lack of commercial formulas), problem is calculation of calorie/proteins, it could be a problem. It is possible only in consultation with hospital nutritionist.

**4**. In LRS, nutritional needs in ARDS patients should be estimated at 20–25 kcal/kg/day and 1.2–1.5 g/kg/day of protein. When commercial feeds are unavailable, hospitals should use safe local ingredients to prepare blenderized enteral formulas with adequate nutrient content.

| Status | Consensus | Agreement |
| --- | --- | --- |
| Consensus not achieved | No | 69.60% |

**Anonymous comments**

1. Constitution of blenderised feeds should be done in consultation of a dietician.
2. Adjusted with progressive increase of caloric intake.
3. No experience here.
4. Same as previous comment.

## Domain 8: Management of complications

**Statements**

**1**. In LRS, pressure ulcer prevention should primarily rely on regular patient repositioning every 2 hours, the use of available pressure redistribution tools (such as improvised cushions, towels, or foam pads), maintaining skin hygiene, and ensuring adequate nutritional support, as these measures are most feasible in resource-limited environments.

| Status | Consensus | Agreement |
| --- | --- | --- |
| Consensus not achieved | No | 74.43% |

**Anonymous comments**

1. Modified air mattresses and barrel beds can be used.
2. Too much is expected from LRS. In my opinion, LRS does not have the ability to treat ARDS.

**2**. In ARDS patients on mechanical ventilation, delirium prevention should primarily focus on minimizing sedation to the lowest necessary levels, ensuring adequate analgesia, implementing early extubation strategies or early tracheotomy, and promoting daily cognitive stimulation, as well as proper sleep-wake cycle management. All available resources in LRS, such as early rehabilitation, should be engaged to support delirium prevention.

| Status | Consensus | Agreement |
| --- | --- | --- |
| Consensus achieved | Yes | 81.15% |

**Anonymous comments**

1. Also Physiotherapy.
2. Engaging family and liberal visitation helps.

**3**. In Low Resource Settings (LRS), preventing hospital-acquired infections in ARDS patients on mechanical ventilation should primarily focus on basic infection control principles, such as strict hand hygiene, minimizing unnecessary interventions, and avoiding the opening of the ventilator circuit.

| Status | Consensus | Agreement |
| --- | --- | --- |
| Consensus achieved | Yes | 80% |

**Anonymous comments**

1. I would add: oral hygiene, head elevation, and use of protective equipment
2. Too much is expected from LRS. In my opinion, LRS does not have the ability to treat ARDS.

# ROUND 2

## Domain 1 - Critical Care Infrastructure Requirements for ARDS Management in Low-Resource Settings (LRS)

**Statements**

1. Patients with ARDS - primarily those with mild forms - who require non-invasive respiratory support modalities (such as HFNO or NIV), or those requiring invasive MV without multi-organ dysfunction syndrome (MODS), should be managed - or transferred to - a level II ICU when a level III ICU is unavailable or overwhelmed. In such cases, support through a Tele-ICU system may provide significant benefit.

| Status | Consensus | Agreement |
| --- | --- | --- |
| Consensus achieved | Yes | 88.18% |

**Anonymous comments**

1. Even though it will help to get space at LIII, in most hospital down referals is hard to implement. If that patient again becomes worse, then need to go back there. This depends on the country ICU availability and their closeness to each other.
2. Time course of ARDS will be different in each case, not all the patients should be treated at level I. Certainly, the initial treatment can be conducted at level I.
3. At least in HDU.
4. Consider distinguishing between HFNO and NIV - the former could be managed at a lower level ICU compared t the latter.
5. It is important to emphasize that in there are “low-resource settings“ within the developed world. Certain areas or populations face limitations in accessing essential resources, such as adequate healthcare, education, or infrastructure, despite being located in a country with generally high standards of living. These limitations can be due to factors like geographic location, socioeconomic disparities, or specific vulnerabilities within the population.
6. In some settings, resource constraints may require initial stabilization elsewhere before transfer, So, while this is ideal, it might not be always feasible everywhere. Also, depending on the clinical context (mild or severe ARDS), some patients might require high level of care.
7. Patients with mild ARDS or those requiring invasive ventilation without multi-organ failure can often be safely managed in Level II ICUs, provided that essential monitoring and respiratory support capabilities are available. The integration of Tele-ICU systems in these settings can enhance clinical oversight, facilitate timely escalation decisions, and ensure adherence to evidence-based protocols, thereby maintaining care standards when Level III ICU beds are unavailable or overwhelmed.
8. Level III could not be available at all, leaving no choice for patient management.
9. this is very important to optimize and relocate resources.
10. I agree that patients with mild to moderate ARDS who require non-invasive respiratory support, or those requiring invasive mechanical ventilation without multi-organ dysfunction syndrome (MODS), can be safely managed in a Level II ICU when a Level III ICU is either unavailable or operating at full capacity. In such cases, the Tele-ICU support can indeed offer substantial clinical and logistical benefits by extending specialist expertise and improving care coordination across ICU levels.
11. I would add “...and at least one non-invasive respiratory support method AND SHORT-TERM. “INVASIVE MECHANICAL VENTILATION...“
12. This would ensure enhanced patient safety.
13. Absolutely. Reduce the pressure to level III as well.
2. Management of the most severe forms of ARDS (particularly in patients with multi-organ dysfunction, hemodynamic instability, and/or those requiring advanced life support measures such as V-V ECMO) should be conducted in specialized centers such as Level III ICUs. In countries classified as LRS that lack such centers, health and governmental health authorities should be engaged and encouraged to recognize the necessity of establishing such facilities. In the early phase of developing such services, the use of Tele-ICU systems may provide meaningful support."

| Status | Consensus | Agreement |
| --- | --- | --- |
| Consensus achieved | Yes | 89.14% |

**Anonymous comments**

1. Should be transferred to level III ICU.
2. ECMO can only be delivered realistically in a subset of Level III ICUs. Not sure its helpful t group ECMO with patients requiring conventional IMV and pressors.
3. MV in an ARDS patient may imply a severe pathology which cannot be managed in a level 1ICU
4. In low-resource settings, the absence of such centers remains a major gap in care equity. Encouraging health authorities to invest in building these specialized facilities is essential for long-term capacity building. Meanwhile, leveraging Tele-ICU systems can serve as a practical interim solution, enabling remote expertise, protocol guidance, and improved outcomes during the early phases of service development.
5. ARDS patients requiring MV should be transferred to level III ICU. I would accept to be transferred to level II ICU IF level II ICU had ventilatory support MV and trained personal. If such ICUs have tele-ICU systems, it MUST have specialized transfer.
6. Completely agree.
7. Patients with ARDS requiring mechanical ventilation typically have moderate to severe disease and require IMV with lung protective strategies. The providers should be skilled with appropriate monitoring ventilatory parameters, proning, etc.
8. this is must to get appropriate care and improve outcome.
9. I agree in this situation that the Tele-ICU platforms can provide vital support by enabling remote specialist input, facilitating clinical capacity building, standardizing protocol implementation, and offering real-time oversight of patient care.
10. Weighted against potential complications during transport. If available, consider activating retrieval team.
11. Shall be admitted to a level III ICU, not in Level II.
12. Strongly agreed.
13. The existence of such support centers is necessary. Also, their existence should be included in the protocol for the care of ARDS patients.
14. I agree however LIC have issues of connectivity to internet and cellular system especially in remote area.
3. In LRS, transporting ARDS patients to Level III ICUs is often challenging due to financial and human resource constraints. Health and political authorities should be encouraged to develop safe transport systems for critically ill patients. When feasible, a mobile expert team from the Level III ICU equipped with advanced support (e.g., V-V ECMO), should be deployed to stabilize and retrieve the patient. Where transport is not feasible, Tele-ICU support may provide critical guidance to lower-level facilities."

| Status | Consensus | Agreement |
| --- | --- | --- |
| Consensus achieved | Yes | 81.58% |

**Anonymous comments**

1. This may be difficult and not practical most of the times. In countries which cannot support these patients even helicopters or air transfer may involve a lot of logistic and administrative difficulties along with laws that may be different in other countries
2. Cost of transporting patients is very high. These resources should rather be used for making those services available in these countries
3. I suggest the most severe forms of ARDS should be managed in both level II and II ICUs. Be reminded that COST is a major issue in LRS and lack of insurance coverage. Most patients pay out of pocket and they can't afford medical evacuation to another facility with ECMO etc. There is also the associated risk with the transport of critically ill patients. Telemedicine can be introduced and advocacy to administrators initiated to get these ICUs adequately equipped. This can work out well if there is support from NGOs for the transfer.
4. This would been very helpful but I am not convinced of it's feasibility and it may rather distract from other more practical and cost-efficient recommendations
5. It is economically more justified and cost-effective to make your own ECMO center.
6. Developing structured critical care transport systems, supported by policy and funding, is essential to ensure equitable access to advanced interventions. The concept of deploying mobile ICU teams with capabilities such as V-V ECMO is an effective strategy seen in high-performing systems and may be adapted gradually. Until such infrastructure is available, Tele-ICU support can offer real-time clinical guidance, help optimize local resources, and potentially improve patient outcomes when transport is delayed or not feasible.
7. Most critical cases of advanced severe ARDS should be treated in ICU 3. level. The transport of patients to the neighbor countries is impractical.
8. This statement is true globally, and not just in LRS countries. A mobile ICU team, even if not ECMO competent, is very useful here.
9. Most LRSs don't have Level III ICUs.
10. It would be challenging, but somebody from the LRS center should be responsible for the lack of care.
11. In such situations, the establishment of a centralized committee specialized in ARDS management, supported by Tele-ICU infrastructure, can provide real-time expert guidance to the local treating teams. This model enhances clinical decision-making and contributes to improved patient outcomes, particularly in settings where direct access to specialized care is limited.

**4**.	Ethical considerations, in accordance with local laws, should guide decisions regarding the transport of ARDS patients to Level III ICUs, particularly to avoid transfers of those with a very poor prognosis, while balancing the potential benefits of higher-level care against the associated burdens and costs."

| Status | Consensus | Agreement |
| --- | --- | --- |
| Consensus achieved | Yes | 88.54% |

**Anonymous comments**

1. These resources should be assigned to training and building capacity in each of the LRS countries.
2. If we do not have level III ICU, adequate legal regulations governing such a sensitive area as critical care patients transport is unlikely.
3. Agreed.
4. I agree with that statement, but it is not critical.
5. Assessment of survival and performing futile practices should be assessed according to the country law and proper communication is crucial while such decision made with family.
6. Ethical considerations, in accordance with local laws, should guide decisions regarding the transport of ARDS patients to Level III ICUs, particularly to avoid transfers of those with a very poor prognosis, while balancing the potential benefits of higher-level care against the associated burdens and costs.
7. In many LRS countries, ethical regulations are lacking.
8. I agree that ethical considerations, aligned with local laws and regulations, as well as the anticipated clinical outcome (e.g., poor prognosis), should guide decisions regarding the transfer of ARDS patients to Level III ICUs. In addition, it is essential to carefully evaluate the safety and feasibility of transport, ensuring that the benefits of higher-level care justify the risks associated with patient transfer. Their should be an eligibility criterion for safe transfer for the critically ill cases.
9. Neighboring LRS could form liaisons and form an ARDS “telemedicine“ joint decision upon initial and further treatment.
10. That is certainly necessary.
11. I fully agree.

## Domain 2 - Diagnostic tools for the identification of ARDS in LRS

**Statements**

**1.** Lung ultrasound (LU), when performed by a trained physician, is a valuable diagnostic tool for ARDS, particularly in emergency departments or shock rooms within LRS, where access to chest X-rays may be limited. LU enables rapid bedside assessment of both pulmonary and cardiac function.

| Status | Consensus | Agreement |
| --- | --- | --- |
| Consensus achieved | Yes | 85.43% |

**Anonymous comments**

1. We suspect that PaO_2_/FiO_2_ ratio will be not eliminated from future definitions of ARDS. Of note, a standardized level of FiO_2_ and PEEP has never been a condition for defining hypoxemia under MV. We suspect that none of proposed indices of oxygenation for ARDS categorization and prediction of outcome will be useful to make clinical decisions unless assessed or calculated using standardized ventilatory settings. Although the S/F ratio is less invasive there are inaccuracies in SpO2 measurements, particularly among patients with darker skin and those in shock and/or with poor distal perfusion. Unfortunately, SpO_2_ is affected by several variables such as changes in temperature, pH, PaCO2, concentration of 2,3-diphosphoglycerate and carboxyhemoglobin, and its measurement is influenced by ethnicity, although none of these variables affect PaO2. SpO_2_/FiO_2_ ratio contains all the problems of the PaO_2_/FiO_2_ ratio, with the added problem that the 95% confidence interval for SpO_2_ vs. SaO_2_ when patient is desaturated, and PaO_2_ values could fluctuate.
2. I agree that lung ultrasound (LU) is a valuable tool for the diagnosis and management of ARDS, particularly in low-resource settings (LRS). LU enables rapid bedside assessment of both pulmonary pathology and cardiac function, supporting timely and informed clinical decisions. I would like to emphasize the importance of regular and structured training to enhance the practical application of LU and improve the accuracy of image interpretation among clinicians. I would prefer to change the statement of a trained physician to a skilled physician in LU.
3. SpO_2_/FiO_2_ could be used as suggested by the Kigali modification, keeping in mind its limitation - SpO_2_ <97% (oxyhemoglobin dissociation curve).
4. LUS may be a particularly useful diagnostic modality within LRS.
5. I think there is growing evidence base supporting SpO_2_/FiO_2_ use as a validated surrogate and the clinicians might feel comfortable using it when P/F ratio is not readily available.
6. LU is a sensitive tool for the diagnosis and severity assessment of ARDS.
7. Any diagnostics are good. Where there is no gas analyzer, the patient has no conditions for treatment.
8. Is SpO_2_/FiO_2_ well validated? How accurate is it and how efficient is it as a tool in the management of ARDS bearing in mind factors that can affect readings of SpO_2_. I agree it should not replace PaO_2_/FiO_2_ ratio.
9. LU provides immediate, bedside evaluation of pulmonary pathology and basic cardiac function makes it particularly valuable in emergency or triage areas where radiologic resources are scarce. When performed by trained clinicians, LU can support early recognition of ARDS, guide initial management, and help differentiate between respiratory and hemodynamic causes of distress—ultimately improving triage accuracy and patient outcomes.
10. Yes, I agree.
11. LU alone cannot assess cardiac function.
12. Very important.
13. Apply ONLY in the absence of gas analyses.
14. I suggest the recommendation is broken into 2, separating the role of S/F ratio from the usefulness comparison to P/F statement.
15. Given the current knowledge base, Sp02/Fi02 could be used as suggested by the Kigali modification. Efforts should be made to better understand possible limitations such as the effect of poor perfusion states and skin color. Ideally, these should be driven by LMIC.
16. Many doctors are not yet trained in LU. Short training is mandatory for practitioners in LIS.
3. The adoption of ultrasound in LRS remains below expected levels and warrants broader promotion. Health and educational authorities are strongly encouraged to integrate ultrasound training across all tiers of medical education - including undergraduate curricula, residency programs, and fellowship - to develop sustainable diagnostic capacity. The implementation of Tele-ICU platforms can play a crucial role in this process by supporting remote training, supervision, and real-time clinical decision-making.

| Status | Consensus | Agreement |
| --- | --- | --- |
| Consensus achieved | Yes | 81.67% |

**Anonymous comments**

1. The inclusion of the chest radiograph criterion remains a question given its moderate-to-poor reliability and limited availability in some settings. Other approaches to radiography in ARDS include computed tomography (CT) scanning (that are uncommon in LRS) and lung ultrasound (US). The use of US requires operating characteristics less known and requires training in image acquisition to meet the definition criteria.
2. Although I appreciate the very important role of LU in the management of the critically ill and ARDS. This recommendation as currently written seems far-reaching and may be problematic to implement in most LRS coupled with the potential resistance from attempt to integrate in undergraduate curricula which are currently stretched.Also, it I have frequently seen it distract from most Emergency medicine trainees and specialists from applying the ABCDE approach to the assessment and management of the critically ill and go straight to the ultrasound even before touching the patient delaying initial resuscitation.
3. Lung ultrasound are not always available in LRS hospitals.
4. I would not mention the global definition, which is not the official definition of ARDS.
5. This is really difficult to do. Where X ray is limited, it is not a place to treat patients with pneumonia, let alone ARDS. Interpretation of a chest X-ray is easier. It is also possible to send the image to other doctors.
6. Mobile chest Xray, blood acid-base balance and blood gas analysis are all basic investigation in all ICUs in European countries. You need it all for right diagnosis of ARDS according Berlin criteria.
7. Some statements, such as this, may be too long to get level of stability required, as one may agree with some, but not all, elements of the statement.
8. LU competencies could be acquired quickly.
9. Should be trained to differentiate other causes of B lines with clinical correlation. Also should incorporate ECHO training. Tele ICU could also help with diagnosing B lines.
10. Promoting ultrasound through structured integration into all levels of medical education is a sustainable strategy to build long-term capacity. Embedding this skill early ensures future clinicians are proficient in point-of-care diagnostics. Additionally, leveraging Tele-ICU platforms to deliver remote mentorship, hands-on supervision, and real-time decision support can accelerate skill acquisition and ensure quality assurance, particularly in geographically remote or underserved areas.
11. Tele and AI play imp role.
12. Indeed, healthcare authorities should adopt a comprehensive strategy to promote the integration of POCUS training across all levels of critical care. Physicians managing critically ill patients, particularly those with ARDS, must undergo regular POCUS training with defined competency benchmarks to ensure safe and effective bedside assessments and interpretation.The Tele-ICU platform offers an ideal model to support this initiative, especially in settings where time, personnel, or geographic constraints limit access to conventional training by enabling remote education, mentorship, and real-time clinical supervision.
13. Is ultrasound more easily available than a simple chest X-ray?

14. POCUS is the future of Critical Care Medicine. The use of LU should be encouraged in our ICUs.

**3.** Ultrasound provides a valuable, non-invasive method for assessing volume status in ARDS patients, especially in LRS, as long as adequately trained personnel are available. To ensure sustainable implementation, health and educational authorities should prioritize ongoing ultrasound education, with Tele-ICU support playing a crucial role during the training and scale-up phase.

| Status | Consensus | Agreement |
| --- | --- | --- |
| Consensus achieved | Yes | 81.40% |

**Anonymous comments**

1. We need more observational studies and RCTs to know whether ultrasound is better that a portable CXR
2. Suggest remove the reference to Tele-ICU here, as it complicates the statement.
3. I strongly agree with this statement. In my opinion, even greater efforts should be made to promote the adoption of lung ultrasound through the development of standardized protocols, structured training programs, and its integration into evidence-based guidelines. This approach would help ensure consistent and effective use of this valuable tool, particularly in low-resource settings where alternatives may be limited.
4. Ultrasound is a reliable alternative to invasive cardiac output measurement. Additionally, pulse pressure variation can be a valuable tool for assessing fluid responsiveness, provided its limitations are acknowledged—particularly in patients with ARDS
5. Even though US is helpful, physical finding and thorough evaluation is also crucial
6. Accurate assessment of volume status in ARDS patients often requires advanced hemodynamic monitoring, which is typically unavailable in many low-resource settings. This recommendation appropriately highlights the value of ultrasound as a practical, non-invasive alternative for bedside evaluation. When performed by trained personnel, ultrasound can provide real-time insights into volume responsiveness and cardiac function, supporting safer fluid management. To ensure sustainable implementation, ongoing education and skill development should be prioritized by health and academic institutions, with Tele-ICU support serving as a vital enabler during the training and scale-up phases.
7. agreement
8. I strongly believe that ultrasound should be a mandatory component in the management of all ARDS cases, regardless of whether care is delivered in low-resource settings (LRS) or high-resource settings (HRS). Its non-invasive nature, ability to be performed bedside, repeatability, and capacity to provide valuable information on both pulmonary and cardiac function make it a useful tool during ARDS management. As previously emphasized, healthcare authorities must prioritize the implementation of sustainable POCUS training programs, integrated across all levels of care. The Tele-ICU platforms can further enhance this effort by supporting remote training, continuous supervision, and real-time clinical guidance, thereby improving both compliance with best practices and patient outcomes.
9. Doctors are not routinely trained in its use and interpretation.
10. The use of ultrasound is certainly a necessary part of the knowledge of a modern doctor. I further emphasize the need for a suitable X-ray room.
11. Absolutely!
12. Understanding its current use in a LRS should be a priority for it to be optimized.

## Domain 3 - Treatment – respiratory support

**1.** In patients with mild to moderate ARDS, non-invasive ventilation (NIV) and high-flow nasal cannula (HFNC) should be considered first-line therapies when hemodynamics are stable and there are no contraindications. Careful patient selection and close monitoring are essential to ensure timely escalation of care at the first signs of clinical deterioration.

| Status | Consensus | Agreement |
| --- | --- | --- |
| Consensus achieved | Yes | 87.08% |

**Anonymous comments**

1. I would consider this recommendation if broken into 2 parts separating the core statement from the second part that seems to disregard the significant risk of NIV by less qualified or experienced providers
2. Here it depends mainly on the clinical situation of the patient. If patient hypoxemic or very tachypneic, we might discuss intubation from start. Another point is how long we should use that… it dam be deleterious to always propose that as a «should».
3. Using both HF and NIV/CPAP requires exact protocols to be able to compare different methods, especially limiting the FiO2 on HF outside of emergency situations (aka preparing for ETI or NIV). Also, using NIV in ARDS can be very challenging; it is more complicated to keep stable spontaneous breathing in acute ARDS than to use controlled IMV.
4. In LRS, there is a lack of modern ventilators and personnel trained in critical care. The use of HFNO has increased in the past decade, particularly during the COVID-19 pandemic. Proponents suggest that the ARDS definition should be modified to allow patients on HFNO to be eligible for the oxygenation criterion even though they are not ventilated with PEEP &#8805;5 cmH2O (as required by the Berlin definition). This approach has not been validated since in many cases those patients had simply hypoxemic ARF. Also, the exact FiO2 is difficult, if not impossible, to be determined in patients on NIV or HFNO. In addition, many patients require intubation and mechanical ventilation IMMEDIATELY.
5. NIV can be more complex to manage and perform adequately than invasive mechanical ventilation.
6. Agree with this statement.
7. I strongly agree with this statement, however I would highlight the need for careful patient selection and close monitoring to detect failing cases early in the statement, since delayed intubation may worsen outcomes.
8. However, if high-flow nasal cannula (HFNC) or non-invasive ventilation (NIV) is used in patients with ARDS, close monitoring is essential to avoid delaying tracheal intubation and the initiation of invasive mechanical ventilation.
9. However, this is practice is monitoring intensive and health care workers should be present constantly monitoring to escalate care as soon as deterioration is recognized.
10. Not sure that NIV in ARDS pts does not require trained personnel.
11. HFNC is a simple, non-invasive tool that can effectively support oxygenation in mild to moderate ARDS and potentially prevent the need for intubation. In resource-limited settings, its use can help preserve critical care resources by reducing the burden on invasive ventilation. However, its success depends on appropriate patient selection and continuous monitoring. Early recognition of clinical deterioration and timely escalation to invasive support when needed are crucial to avoid delays that may negatively impact outcomes.
12. Full agreement.
13. Strongly agree.
14. I fully support this approach. When implementing non-invasive ventilation (NIV) in patients with ARDS, it is imperative that clinicians maintain a high level of vigilance for patient self-inflicted lung injury (P-SILI). Continuous assessment of respiratory effort, tidal volume, and work of breathing is crucial to avoid worsening lung injury in spontaneously breathing patients. Early recognition of excessive inspiratory effort is key to making timely decisions regarding escalation to invasive mechanical ventilation, thereby preventing delayed intubation and the progression of lung damage. To strengthen the clinical vigilance and ensure early intervention, there is a pressing need for the development and implementation of a dedicated clinical assessment tool for P-SILI.
15. We need to provide an estimated timeframe for response assessment for NIV and HFNC. A suggested approach to non-response will provide a safety net, avoiding delayed invasive mechanical ventilation and the associated harms of delay.

**2.** Upon admission to the intensive care unit, both the patient with ARDS (if able to participate) and their family should be informed about the risks, costs, burdens, and alternatives to intubation, including the potential need for prolonged mechanical ventilation and tracheostomy. They should have the opportunity to participate in decisions regarding the forgoing of life-sustaining treatment, in accordance with local laws and regulations.

| Status | Consensus | Agreement |
| --- | --- | --- |
| Consensus achieved | Yes | 83.02% |

**Anonymous comments**

1. This can make it difficult to treat the patient. It can delay intubation and the use of mechanical ventilation. It is not easy to obtain the consent of the patient and family members. Sometimes the patient is not conscious, the family does not come, etc.
2. I agree with the first part of statement. But, according to the second part of the statement, in many LRS, such as Bosnia and Hercegovina, patient or relatives do not have right do decide to e.g. stop prolonged ventilation, or stop life-sustaining treatment.
3. Agree with this statement.
4. I think it is very important.
5. Early, transparent discussions with patients and families about the risks, burdens, and potential trajectories of mechanical ventilation—including the possibility of prolonged support or tracheostomy are essential for informed consent and shared decision-making. Involving families in advance care planning fosters trust, aligns treatment with patient values, and helps guide decisions about the initiation or limitation of life-sustaining interventions, all within the framework of local laws and cultural norms.
6. Yes I agree.
7. As longer as is the family who will pay for the care, it must be associated to decision making and be informed continuously on what is going on to their patient.
8. The use of HFNO has increased in the past decade, particularly during the COVID-19 pandemic. Proponents suggest that the ARDS definition should be modified to allow patients on HFNO to be eligible for the oxygenation criterion even though they are not ventilated with PEEP &#8805;5 cmH2O (as required by the Berlin definition). This approach has not been validated since in many cases those patients had simply hypoxemic ARF. Also, the exact FiO2 is difficult, if not impossible, to be determined in patients on NIV or HFNO. Patient/relatives preferences should be taken in CONSIDERATION.
9. Effective communication between the healthcare team and the patient and/or their family is essential to ensure that care aligns with the patient’s best interests and personal wishes.
10. Family engagement is key.
11. A family-centered care approach is essential, ensuring that patients and their families are actively involved in shared decision-making, especially regarding the initiation or continuation of life-sustaining treatments. This should by guided by local laws, cultural values, and ethical standards.
12. A clear therapeutic plan is crucial and, as such, must be communicated to the patient and their relatives, letting them know what will be done (if needed) and what will not be done (withholding).

**3.** In LRS, noninvasive ventilation (NIV/HFNC) may be used for patients with severe ARDS for a short period to provide valuable initial time to stabilize the patient, ensure adequate pre-oxygenation, and create conditions for safe intubation. A period of 1 to 2 hours is an appropriate timeframe to wait for patient stabilization, with continuous monitoring and readiness for rapid and timely intubation if needed.

| Status | Consensus | Agreement |
| --- | --- | --- |
| Consensus achieved | Yes | 85.74% |

**Anonymous comments**

1. The use of HFNO has increased in the past decade, particularly during the COVID-19 pandemic. Proponents suggest that the ARDS definition should be modified to allow patients on HFNO to be eligible for the oxygenation criterion even though they are not ventilated with PEEP 5 cmH_2_O (as required by the Berlin definition). This approach has not been validated since in many cases those patients had simply hypoxemic ARF. Also, the exact FiO_2_ is difficult, if not impossible, to be determined in patients on NIV or HFNO.
2. Question 1 and 3 are similar but the severity changes. However the definition of severity on p/f ratio or sao2/fio2 is difficult to predict.
3. Agree with this statement.
4. Maybe provide some indications on tools or score to use to predict NIV/HFNC failure?
5. Based on my experience in low-resource settings, teams often overlook patients receiving NIV or HFNC support and underestimate the importance of close monitoring to promptly identify the need for intubation. Maybe, incorporating recommendations for a maximum duration of NIV/HFNC use and establishing clear monitoring protocols to ensure timely escalation of care would be valuable additions to this statement.
6. A defined window of 1 to 2 hours is appropriate, as it allows for pre-oxygenation and preparation while minimizing delays. Prolonging non-invasive support beyond this period in patients who are not improving may increase the risk of complications, including self-inflicted lung injury and higher mortality. Continuous monitoring and readiness to intubate are essential to ensure patient safety and optimize outcomes.
7. Time interval for 1-2 hours to test effectiveness of NIV /HFNC.
8. A short trial of non-invasive ventilation (NIV or HFNC) may be attempted under close monitoring, but without delaying tracheal intubation, as such a delay could lead to increased risk of mortality in these patients.
9. I agree with the statement. But we need a validated tool for clinical decision-support to assist physicians in determining the optimal timing for intubation in patients with ARDS in the LRS. This is crucial to avoid delays in transitioning from non-invasive to invasive ventilation, as such delays may lead to worse outcomes due to progressive hypoxemia, increased work of breathing, or patient self-inflicted lung injury (P-SILI).
10. The use of ROX (for HFNC) and HACOR (NIV) is mandatory when assessing therapeutic success or failure. Scoring assessment should be performed at regular time intervals.
11. It is extremely important to recognize the need for intubation and mechanical ventilation in time,

**5.** To substantially reduce ventilation errors and improve the implementation of lung-protective strategies, simple nomograms displaying predicted body weight and corresponding tidal volume settings for men and women, as well as toxic FiO2 concentrations and dangerous Ppeak and Pplat levels, should be physically attached and prominently visible on all ventilators.

| Status | Consensus | Agreement |
| --- | --- | --- |
| Consensus achieved | Yes | 85.44% |

**Anonymous comments**

1. Not practical in LRS
2. I think that the decision about intubation and mechanical ventilation of a patient with ARDS should not be related to the probability of benefit, ethical considerations and local legal frameworks.
3. There is no consensus on what toxic FiO_2_ is; the same goes for peak pressure.
4. Agree with this statement.
5. Not sure what a toxic FiO_2_ is. There is no cutoff or definition in the literature about a toxic FiO_2_. Also, I would remove the Ppeak pressure and replace it with Driving pressure.
6. Many patients require intubation and MV immediately.
7. This recommendation offers a practical and impactful solution to enhance adherence to lung-protective ventilation strategies. By displaying nomograms with predicted body weight and corresponding tidal volumes, as well as thresholds for harmful FiO2, peak, and plateau pressures, clinicians are provided with immediate visual guidance at the bedside. This can help minimize human error, particularly in high-stress or resource-limited environments, and reinforces best practices in real time. Simple visual tools like these can play a critical role in standardizing care and improving patient safety across all ICU settings.
8. It is logical to think that nomograms for PBW, plateau pressure, FiO_2_, PaO_2_, etc. are like checking list in the airplanes!
9. Including Also Driving pressure.
10. Implementing such an approach can help standardize the management of ARDS, which is valuable. However, ventilation strategies must be individualized, as each patient presents with unique pathophysiology and evolving clinical dynamics. The concept of static ventilation is now outdated. We are in the era of dynamic MV, where support is tailored in real-time to the patient’s lung mechanics, emphasizing adaptive, dynamic lung-protective strategies that respond to ongoing changes in lung compliance, driving pressure, and patient effort and consequently achieving the targeted mechanical power. If the dynamic ventilation strategy is available, we can encourage using it in the initial stages of invasive MV.
11. Full agreement.

**6.** In mechanically ventilated ARDS patients, the initial PEEP should be set within the range of 5-10 cm H2O, avoiding levels below 5 cm H2O, and subsequently titrated according to the patients clinical status, including oxygenation and hemodynamic parameters, to maintain a plateau pressure below 30 cm H2O. It is important to consider that in patients with left ventricular failure or right heart failure, PEEP levels may need to be adjusted upward or downward based on clinical response and tolerance.

| Status | Consensus | Agreement |
| --- | --- | --- |
| Consensus achieved | Yes | 92.34% |

**Anonymous comments**

1. If under initial PEEP we mean the PEEP set at intubation and immediately corrected using specific methods (LR assessment - LRM, PV loops, TPP etc.), then yes. PEEP individualization is the cornerstone of mechanical ventilation in ARDS, regardless of whether it's 5, 10, 15 or 25cmH2O.
2. This is again quite a complex statement, which may be why there is dissent. One can agree with most but not all components (e.g. suggestion to avoid PEEP <5)
3. I fully agree and support this statement. On the other hand, I sometimes wonder if this might become a barrier in front of ongoing training and capacity building to expand mechanical ventilation skills over time. Because a small subset of ARDS cases may benefit from advanced (fancy!) or less commonly used ventilation modes. When feasible, consultation with more experienced centers should be encouraged and this may help improve outcomes and support skill development of the local physicians.
4. We should not forget the driving pressure to keep it less than 15 cmH_2_O whenever possible.
5. they could also be guided by experts by utilizing Tele ICU
6. It would be useful to survey this to ensure appropriate educational programs are in place
7. There is disconnection between the conceptual model of ARDS—a specific type of inflammation and the lack of measures of inflammation in ARDS definitions. Diagnostic accuracy in ARDS is challenging without a universal reference standard. Future work in refining the ARDS definition should carefully consider other facets of validity. Clinicians may wish to use the broader umbrella syndrome of acute hypoxemic respiratory failure when deciding to implement certain therapeutic strategies, particularly those that are not directed against specific ARDS mechanisms. However, many hypoxemic patients who improve after 24 h of standard intensive care do not have ARDS. We do not cure ARDS in 24 h.
8. The effects of PEEP are more pronounced on a failing right ventricle than on the left, as PEEP can significantly increase right ventricular afterload by raising pulmonary vascular resistance. Therefore, right ventricular function should be closely monitored using ultrasound, with particular attention to signs of acute *cor pulmonale*.
9. The statement emphasizes the starting PEEP and plateau pressure (<30 cm H2O) and subsequently the targeted plateau pressure which are a recognized as a core elements of the lung-protective ventilation strategy and known predictors of patient outcomes. The statement stress on the hemodynamic PEEP effect during RV and LV failure but the targets of the PEEP and plateau pressure during MV during RV and LV failure need to be defined. We are lacking the protocol for RV protective ventilation strategy and LV protective ventilation strategy.
10. This recommendation outlines a safe and individualized approach to PEEP management in mechanically ventilated ARDS patients. Starting within the 5–10 cmH_2_O range helps avoid the risks associated with both under-recruitment and excessive intrathoracic pressure. Titration based on oxygenation, hemodynamics, and maintenance of plateau pressure <30 cmH_2_O aligns with lung-protective ventilation principles. Importantly, the recommendation acknowledges the need to adjust PEEP in the context of cardiac comorbidities such as left or right heart failure where overly high or low PEEP can adversely impact preload, afterload, or pulmonary pressures. Tailoring PEEP to individual physiology is key to optimizing outcomes.

**7.** For hemodynamically stable patients with ARDS, following an assessment of ventilator recruitability, a brief recruitment maneuver lasting less than 40 seconds should be considered. Subsequently, ventilator settings for PEEP and FiO2 should be adjusted to the minimal effective levels necessary to maintain optimal oxygenation. Careful monitoring of the patient’s clinical status during and after the recruitment maneuver is essential for the timely identification of any adverse events.

| Status | Consensus | Agreement |
| --- | --- | --- |
| Consensus achieved | Yes | 85.45% |

**Anonymous comments**

1. Recruitment maneuvers (RMs) should not be performed routinely but rather reserved for carefully selected patients - particularly those with severe hypoxemia - as automatic or indiscriminate use may be harmful.
2. Since the Arnal ICM study in 2011. We know that RM should not last more than 10-15 seconds to ensure almost maximum possible recruitment with minimal hemodynamic compromise. This study has never been disputed.
3. The current Recommendations do not recommend routine use of RMs in ARDS patients.
4. I prefer to use 4 - 6 mls / kg
5. I am of the opinion that where there is a mechanical ventilation device, intensivists and anesthesiologists need to be better trained in its use.
6. Agree with this statement - have increased score to 8.
7. 4 -6ml/kg.
8. Many critical care physicians use brief RM to open up the lungs. Current standard (conventional) MV include low tidal ventilation for all patients with acute respiratory failure. All mammals are scaled similarly and the VT is about 6.2 ml/kg “ideal“ body weight.
9. The trigger for the RM should be defined such as hypoxemia (PaO_2_ or SpO_2_ or P/F ratio) level. The recruitable lung needs to be defined and a validated tool or protocol to assess the recruitabilty needs to be defined. The hemodynamic stability needs to be defined as well (SBP or MAP Target, Vasopressor requirements).
10. RM when appropriately applied, such maneuvers may improve alveolar recruitment and oxygenation without prolonged exposure to high airway pressures. Emphasis on limiting duration to under 40 seconds minimizes the risk of barotrauma and hemodynamic compromise. Adjusting PEEP and FiO_2_; to the lowest effective levels post-recruitment aligns with lung-protective strategies. Close clinical monitoring during and after the maneuver is essential to detect adverse effects and ensure patient safety.
11. Agree.

**8.** Prolonged RMs lasting more than 60 seconds should be avoided in low-resource settings (LRS) due to the increased risk of serious adverse effects, including hemodynamic instability, barotrauma, and biotrauma.

| Status | Consensus | Agreement |
| --- | --- | --- |
| Consensus achieved | Yes | 86.08% |

**Anonymous comments**

1. Since the Arnal ICM study in 2011. We know that RMs should not last more than 10-15 seconds to ensure almost maximum possible recruitment with minimal hemodynamic compromise. This study has never been disputed.
2. Okay, that's the basics. But where are the FiO2, PEEP, the frequency, plateau pressure....
3. Agree with this statement - although it does not just apply to LRS.
4. not only in LRS, but in all the world...
5. This recommendation is supported by growing evidence that prolonged recruitment maneuvers—especially those lasting more than 60 seconds—can cause harm, including barotrauma, hemodynamic instability, and increased biotrauma, without consistent benefit in improving clinical outcomes. The ART trial (Alveolar Recruitment for ARDS Trial, NEJM 2017) is a key study in this context: it evaluated an aggressive recruitment and PEEP titration strategy in moderate-to-severe ARDS and was terminated early due to increased mortality in the intervention group, largely attributed to higher rates of hypotension and barotrauma. These findings suggest that prolonged recruitment maneuvers may introduce more risk than benefit, particularly in settings where close hemodynamic monitoring and rapid intervention capabilities are limited. Therefore, avoiding such maneuvers in low-resource settings aligns with a safer, evidence-based approach to ARDS management.
6. In fact, I strongly recommend to all MV industries to include the height and the sex in all ventilators, and to develop algorithms calculating the recommended VT. Also, I recommend the design of alarms in ICU patients that are ventilated, showing the tidal volumes applied.
7. Totally agree with the statement. It is evidence based. Based on the ART trial which demonstrated increased mortality and barotrauma associated with aggressive prolonged recruitment maneuvers.
8. Should not be used might be better.
9. Yes, good suggestion.

**9.** Prone positioning should be considered in patients with moderate to severe ARDS and severe hypoxemia (defined as a PaO2/FiO2 ratio below 150 or an SpO2/FiO2 ratio below 200), provided that all necessary conditions related to staff expertise and patient safety are adequately met.

| Status | Consensus | Agreement |
| --- | --- | --- |
| Consensus achieved | Yes | 81.81% |

**Anonymous comments**

1. I would give a range: 5 to 10 cm of water. Higher PEEP should be considered in specific settings. Maybe avoid lower values than 5.
2. This would be very frivolous. Operating a mechanical ventilation machine is a serious science. The parameters change from hour to hour. Where there is no trained staff, patients with ARDS should not be treated.
3. I am not sure about the strong evidence that supports initial PEEP should be 10 cmH_2_O.
4. Agree with this statement.
5. The requirement for PEEP should not be removed regardless of oxygen delivery device used. PEEP is a routine approach even in the operating rooms. If you removed PEEP, patients labeled as ARDS, do not have ARDS. We need to standardize the level of PEEP and the level of FiO_2_ to calculate PaO_2_/FiO_2_. A standardized level of FiO_2_ and PEEP has never been a condition for defining hypoxemia under MV. We suspect that none of proposed indices of oxygenation for ARDS categorization and prediction of outcome will be useful to make clinical decisions unless assessed or calculated using standardized ventilatory settings. In patients fulfilling ARDS criteria, assessment at 24 h on PEEP≤10 cmH_2_O with FiO_2_≤0.5 for 30 min caused PaO_2_/FiO_2_ ratio to increase, such that more than a third of patients no longer met ARDS criteria. If patients do not have gas-exchange compatible with ARDS, they DO NOT HAVE ARDS. ARDS is nor curable in less that 24 hours, if you accept the definition as “an intense inflammatory process of the lungs“.
6. There is a clear and undeniable benefit of prone ventilation in moderate and severe ARDS.
7. Should be used than considered.
8. This appears to be a practical approach.
9. Despite its proven benefits, prone positioning remains underutilized in low-resource settings. When appropriately applied, it can significantly improve oxygenation and gas exchange, and may also reduce right ventricular strain by decreasing pulmonary vascular resistance.
10. Totally agree. Despite that the Use of SpO_2_/FiO_2_ as a cutoff value for proning is less commonly standardized than PaO_2_/FiO_2_. Further high-quality evidence is needed to validate specific SpO2/FiO2; thresholds for guiding proning decisions. Define the optimal timing for initiating prone positioning. We need to ensure implementation of a structured checklist prior to initiating proning to ensure patient safety, optimize effectiveness, and standardize the process.
11. Full agreement.

**10.** If prone positioning leads to improved oxygenation and arterial blood gases (ABG), intubated patients with moderate to severe ARDS in low-resource settings (LRS) should be maintained in the prone position for more than 12 hours per day, provided that all necessary conditions related to staff expertise and patient safety are met.

| Status | Consensus | Agreement |
| --- | --- | --- |
| Consensus achieved | Yes | 86.04% |

**Anonymous comments**

1. No needs to hold breath for this.
2. I'm disoriented. I don't know who is treating the patient, a nurse, a doctor, or someone else. Maybe I take mechanical ventilation too seriously.
3. Lung protective ventilation includes the use of low VT (4-8 ml/kg PBW), a plateau pressure <29 cmH_2_O, and moderate levels of PEEP (8-12 cmH_2_O). In LRS, lack of staffing is common for putting a patient on prone. In addition, the PROSEVA trial must be validated!
4. 0.5-1 seconds is enough.
5. There is a good evidence base for this - globally.
6. Short paralysis of the patient?
7. This recommendation aligns with strong clinical evidence supporting prone positioning in moderate to severe ARDS with significant hypoxemia (PaO_2_FiO_2_<150). The landmark PROSEVA trial (NEJM 2013) demonstrated a significant mortality benefit when prone positioning was initiated early and applied for prolonged sessions in this patient group. Ensuring adequate staff expertise and strict adherence to safety protocols is essential to minimize complications such as pressure injuries or accidental dislodgement of tubes and lines. In resource-limited settings, investing in staff training and standard operating procedures can help safely implement this highly effective, low-cost intervention.
8. Good idea.
9. Prolonged prone positioning—up to 16 hours per day—has been shown to enhance oxygenation and may also offer protective effects on “right ventricular function“.
10. I would say more than 14 hours.
11. Totally agree for that statement. Evidence based despite the optimal duration and number of sessions remain areas where definitive evidence is limited. This highlights the need for ongoing research to clarify how long each prone session should optimally last beyond the 16-hour benchmark and how many total sessions are ideal before assessing treatment success or failure.
12. There is a clear and undeniable benefit of prone ventilation in moderate and severe ARDS.
13. I would add even mechanics improvements compliance and driving pressure even no oxygenation improvement.

**12.** The Berlin Definition has long served - and continues to serve - as the foundational framework for diagnosing acute respiratory distress syndrome (ARDS). However, the challenges highlighted by the COVID-19 pandemic have exposed important limitations of this definition. Specifically, it fails to account for the critical roles of non-invasive ventilation (NIV and HFNO) and point-of-care (POC) ultrasound in the modern diagnosis, monitoring, and management of ARDS. A redefinition of ARDS is warranted to integrate these widely adopted clinical practices and recent advancements, to improve diagnostic accuracy and patient outcomes.

| Status | Consensus | Agreement |
| --- | --- | --- |
| Consensus achieved | Yes | 84.29% |

**Anonymous comments**

1. It is possible that patients requiring NIV and HFNO do not have ARDS. What about if the ga-s exchange is higher than 300 mmHg in patients under HFNO? Although ARDS is a type of AHRF, most patients with AHRF DO NOT HAVE ARDS. Concerns with the ART trial design, methodology, data analyses, and results—in addition to possible major differences with health care systems—provide solid arguments to question the results of the ART trial.
2. While the Berlin Definition remains a foundational tool, its reliance on invasive ventilation and chest radiography limits its applicability in modern, resource-diverse settings. The widespread use of non-invasive modalities such as HFNO and NIV, along with the growing utility of point-of-care ultrasound for detecting pulmonary pathology, underscores the need for a more inclusive and flexible definition. Updating the criteria to reflect these advances would not only enhance diagnostic accuracy across varying levels of care but also facilitate earlier intervention and more tailored management strategies.
3. Yes, you are right.
4. My concern here is that the Global ARDS definition does already address these issues, so not sure we need another ARDS definition
5. Problem with the ARDS classification is that by including any kind of therapy in the definition influences the end result - aka Flow in HF or PEEP in NIV influence the PaO_2_/FiO_2_ thereby masking the actual disease severity - patient still has severe ARDS just the severity is masked by improvement in PaO_2_, SpO_2_ by flow/peep
6. A pragmatic definition of ARDS tailored to low-resource settings is urgently needed—particularly one that incorporates the SpO_2_/FiO_2_ ratio as a surrogate marker in situations where arterial blood gas analysis is not readily available. Including PEEP level in this revised definition could further enhance its diagnostic utility.
7. In my view recent “global“ definition is an improvement over the previous (Berlin).
8. Totally agree.
9. It can be used in intensive care units, but routine use is not recommended.
10. In my opinion it should not be done.
11. The mechanical ventilation device should be located where trained personnel are located.

**Domain 4 - other supportive therapies**

**Statements**

**1.** In patients with ARDS, the duration of corticosteroid therapy should not exceed 7 to 10 days, which corresponds to the exudative phase of the syndrome. Tapering is unnecessary when corticosteroids are administered within this timeframe.

| Status | Consensus | Agreement |
| --- | --- | --- |
| Consensus achieved | Yes | 82.73% |

**Anonymous comments**

1. The dose and duration of corticosteroid therapy in ARDS should be tailored to disease severity and the risk of relapse. The SCCM has published a comprehensive protocol for corticosteroid use in both community-acquired pneumonia (CAP) and ARDS. This protocol distinguishes between resolving and non-resolving ARDS and provides detailed guidance on dosing and duration. The duration of corticosteroids and consequently the tapering should be individualized.
2. There are multiple trials of Steroids for ARDS currently in progress, a clear indication that the role of steroids in ARDS is not resolved.
3. No reason not to taper
4. We do not have evidence to support this statement.
5. No evidence
6. This recommendation aligns with the pathophysiological understanding of ARDS and the current evidence base, which supports limiting corticosteroid therapy to the exudative phase—typically within the first 7 to 10 days. Short-course therapy during this phase can help control inflammation without increasing the risk of adverse effects associated with prolonged use. Studies, including the DEXA-ARDS trial (Lancet Respir Med 2020), support this time-limited approach. Additionally, when corticosteroids are discontinued within this duration, tapering is generally unnecessary, as the risk of adrenal suppression is minimal, especially with doses under 20 mg dexamethasone or equivalent. This strategy promotes safety and simplifies clinical management.
7. Around one week is sufficient, with de-escalation.
8. According to the DEXA-ARDS trial (Lancet Respir Med 2020) no more than 10 days has a beneficial effects in the primary and secondary outcomes.

**2.** In patients with moderate to severe A RDS, consideration should be given to using neuromuscular blocking agents (NMBAs) during the first 48 hours in cases of persistent ventilator dyssynchrony despite deep sedation. If muscle relaxation remains necessary after this period, based on the clinical condition, ventilator dyssynchrony, and arterial blood gas analysis, intermittent administration of NMBAs is preferred over continuous infusion.

| Status | Consensus | Agreement |
| --- | --- | --- |
| Consensus achieved | Yes | 80.29% |

**Anonymous comments**

1. While the likely mechanisms of action of NMBAs in early ARDS is to reduce ventilator dyssynchrony, there is no evidence for later use of NMBAs. Perhaps split this statement into 2 separate statements?
2. The ACURASYS trial showed that in patients with moderate-to-severe ARDS, a strategy of 48 hours of deep sedation with muscle paralysis induced by an intravenous infusion of cisatracurium resulted in a lower incidence of barotrauma and higher adjusted overall survival at 90 days than deep sedation alone. These results were unexpected, since the intervention was performed only for the first 2 days, yet the Kaplan–Meier survival curves were virtually superimposable for about 18 days before they separated.
3. I would only consider NMBA if there is ventilatory dyssynchrony despite deep sedaton.
4. Requires training.
5. and moderate severe.
6. However, intensivists should ensure that sedation is adequately optimized and ventilator settings are appropriately adjusted before initiating NMBA therapy. In addition, monitoring the depth and duration of neuromuscular blockade is essential. This monitoring is relatively inexpensive and can help guide therapy, minimizing the risk of both short-term complications and long-term consequences, such as ICU-acquired weakness.
7. This only has evidence for cisatracurium and extending this to all NMBA leads to overuse of NMBA and increased incidence of CINM, and increased LOS MV and LOS ICU.
8. If necessary.
9. This recommendation reflects a nuanced, evidence-informed approach to NMBA use in moderate to severe ARDS. Early administration within the first 48 hours—particularly in patients with persistent ventilator dyssynchrony despite deep sedation may improve oxygenation and facilitate lung-protective ventilation. While earlier studies such as the ACURASYS trial suggested potential benefit, more recent findings from the ROSE trial (NEJM 2019) highlight the importance of selective rather than routine NMBA use. Prolonged continuous infusion should be avoided due to the risk of ICU-acquired weakness; therefore, if ongoing neuromuscular blockade is needed, an intermittent strategy guided by clinical and gas exchange parameters is preferred to balance efficacy with safety.
10. I agree with the statement. The statement need to emphasize on monitoring of sedation and muscle relaxation. Targets of sedation during proning should be score defined such as RASS score or BIS monitor targets. Targets for muscle relaxation should be defined based on TOF for example.
11. Agreement.

## Domain 7 – Nutrition

**Statements**

**1.** Critically ill patients, especially in low-resource setting, are at risk of malnutrition and vitamin deficiencies. Risk assessment should be performed using validated tools such as the mNUTRIC score, NRS-2002, or Subjective Global Assessment (SGA) to identify patients at risk of malnutrition.

| Status | Consensus | Agreement |
| --- | --- | --- |
| Consensus achieved | Yes | 82.20% |

**Anonymous comments**

1. Initial dose is important - low dose better (6-10 kcal/kg/day)?
2. Good recommendation but needs to be simplified for clarity.
3. I agree that these tools are validated, but not specifically in LRS countries.
4. Initiation shall not be delayed.
5. Importantly, nutritional assessment should rely on basic clinical resources readily available in the setting, without requiring access to advanced laboratory or biological markers that may not be feasible in such environments.
6. assessment for risk of malnourishment should be done. All patients should be considered to be at risk for malnourishment. they may also present with vitamin deficiencies which would need replacement/ supplementation.
7. “Trophic“ (small amount) can be done without checking the aspirate. In general, there is no need for full feeding during the first week.
8. Gastric aspirate of 250mls every 4 hours.
9. This recommendation appropriately emphasizes the importance of early nutritional risk assessment in critically ill patients, especially in low-resource settings where malnutrition and micronutrient deficiencies are common and often overlooked. Among available tools, the modified NUTRIC (mNUTRIC) score is particularly practical, as it does not require laboratory values, making it suitable for use in settings with limited diagnostic capacity. It has been validated as a reliable predictor of nutrition-related outcomes in the ICU. While tools like NRS-2002 and SGA are also useful, the simplicity and applicability of mNUTRIC make it especially valuable for routine use in resource-constrained environments.
10. Totally agree for the statement.
11. Daily nutritional screening is required. When all conditions are met, nutritional therapy is initiated. Enteral nutrition is always the first choice.

**2.** In LRS, where indirect calorimetry or other precise assessment tools are unavailable, nutritional needs in ARDS patients can be estimated at 20–25 kcal/kg/day for energy and 1.2-2g/kg/day for protein. If predictive equations are used to estimate the energy need, hypocaloric nutrition (below 70% estimated needs) should be preferred over isocaloric nutrition for the first week of ICU stay.

| Status | Consensus | Agreement |
| --- | --- | --- |
| Consensus achieved | Yes | 84.06% |

**Anonymous comments**

1. 1.2 protein is enough based on last studies.
2. Agree.
3. As predictive equations have been shown to be inaccurate, a practical approach is to apply a simplified formula—20 to 25 kcal/kg/day for energy and 1 to 2 g/kg/day for protein—which is easy to use in daily clinical practice.
4. And regular monitor (gastric residual volume, abdominal distension, diarrhea, vomiting) should be done.
5. This recommendation provides a practical and evidence-informed approach to nutritional support in ARDS patients where indirect calorimetry is not available—a common scenario in low-resource settings. Estimating energy needs at 20–25 kcal/kg/day and protein at 1.2–2 g/kg/day aligns with international guidelines such as those from ESPEN and ASPEN. Early hypocaloric feeding (i.e., <70% of estimated energy needs) during the first ICU week has been shown to reduce complications such as overfeeding, hyperglycemia, and refeeding syndrome, particularly in critically ill patients. This strategy is both safe and feasible, supporting metabolic stability while minimizing risks in resource-limited environments.
6. Totally agree.
7. Introduce nutritional therapy gradually.

**3.** In hemodynamically stable patients with ARDS, enteral nutrition (EN) should be initiated within 48 hours of ICU admission. Initial nutrition should begin with a low-dose trophic approach (6–10 kcal/kg/day), with progressive advancement based on patient tolerance. Caloric and protein intake targets should be approached gradually, increasing intake by 5 to 10% of the calculated total caloric needs per day, aiming to reach full requirements by days 5 to 7 of ICU stay, as tolerated. Parenteral nutrition (PN) should only be considered when EN is contraindicated or not tolerated after 3-7 days.

| Status | Consensus | Agreement |
| --- | --- | --- |
| Consensus achieved | Yes | 80.24% |

**Anonymous comments**

1. Multiple different aspects covered here, which may account for the disagreement.
2. Nutrition in LRS depends on availability and costs. In case od hospital-prepared food (and the lack of commercial formulas), problem is calculation of calorie/proteins, it could be a problem. It is possible only in consultation with hospital nutritionist.
3. This recommendation reflects current best practices for nutritional support in ARDS patients, consistent with ESPEN and ASPEN guidelines. Initiating early enteral nutrition (EN) within 48 hours in hemodynamically stable patients supports gut integrity, reduces infectious complications, and improves outcomes. A trophic feeding strategy (6–10 kcal/kg/day) with gradual advancement allows for safe progression while minimizing the risk of intolerance. Targeting full caloric and protein needs by days 5–7 ensures adequate support without overfeeding. Parenteral nutrition (PN) should be reserved for cases where EN is not feasible after 3–7 days, reinforcing a cautious and stepwise approach in both high- and low-resource settings.
4. Clinicians should recognize that enteral nutrition is more cost-effective and generally safer than parenteral nutrition, making it the preferred option in both resource-rich and low-resource settings.
5. Totally agree, but in malnourished or high-risk patients, early supplemental PN (after 2–3 days) may be justified if EN is not feasible.
6. Use parenteral nutrition as a supplement to enteral nutrition to achieve nutritional goals.
7. Agree.

4. Enteral nutrition via nasogastric tube should be the standard approach. In LRS settings, where feeding pumps are often unavailable and there is a frequent shortage of nursing staff, gravity-assisted feeding is a practical alternative. Feeding volumes should be adjusted according to patient tolerance, and regular assessment for intolerance (gastric residual volume, abdominal distension, vomiting, and diarrhea) remains essential.

| Status | Consensus | Agreement |
| --- | --- | --- |
| Consensus achieved | Yes | 87.07% |

**Anonymous comments**

1. adjusted with progressive increase of caloric intake.
2. No need to routinely measure gastric residual volume.
3. Overall agree - statement covers 3 related but distinct issues, which may account for dissent.
4. Routine measurement of gastric residual volume is not necessary; monitoring for other signs of feeding intolerance—such as abdominal distension, vomiting, and diarrhea—remains essential for ensuring patient safety and effective nutritional support.
5. Regular assessment of gastric residual is not recommended for ICU patients.
6. constitution of Blenderised feeds should be done in consultation of a dietician.
7. I agree with this recommendation, particularly the emphasis on nasogastric feeding and the practicality of gravity-assisted delivery in low-resource settings. However, routine monitoring of gastric residual volume (GRV) is no longer supported by current evidence. Trials such as REGANE and NUTRIREA-1 have shown that skipping routine GRV checks does not increase the risk of aspiration and may improve overall nutrition delivery. Instead, clinical signs like abdominal distension, vomiting, and diarrhea should guide assessments of feeding tolerance.
8. No experience here.
9. Totally agree.
10. Same as previous comment.
11. Agree.

**ROUND 2 - Consensus not achieved**

**Domain 3 - Treatment – respiratory support**

**Statements**

4. The utilization of prone positioning may be considered in awake ARDS patients receiving non-invasive ventilatory support (NIV/HFNC), provided that all necessary conditions regarding staff expertise and patient safety are met, along with appropriate patient participation and tolerance.

| Status | Consensus | Agreement |
| --- | --- | --- |
| Consensus not achieved | No | 79.45% |

**Anonymous comments**

1. Data on long-term outcomes from awake proning in non-COVID-19 ARDS (intubation rates, mortality) are still limited and inconclusive.
2. according to the severity of disease, participation of the patient, agitation and skills of the team
3. in Resource-limited settings there is lack of personnel to do proning in patients with ARDS or in severe AHRF.
4. The use of prone positioning may be considered in awake ARDS patients receiving non-invasive ventilatory support (NIV or HFNC). However, the evidence supporting prone positioning in awake ARDS patients receiving invasive mechanical ventilation remains limited and is not strongly supported by high-quality evidence.
5. Need to be extra cautious.
6. This is pending further research confirming usefulness outside COVID-19.
7. Semi prone is OK.
8. If the expertise, safety and staffing requirements are available should be added to the recommendations for me to consider it.
9. very cheap and effective treatment should be done.
10. I agree with this statement, but patient tolerance is one of the key aspects of prone positioning and this should be closely monitored. And it is not a substitute for escalation to invasive mechanical ventilation when needed.
11. We do not have strong evidence in non-COVID ARDS patients.
12. Human resources, staffing levels and training - questionable, patient safety, risk?
13. Should this be only in awake patients? could also be applicable in intubated patients.
14. This recommendation is grounded in evidence from the COVID-19 pandemic, during which awake prone positioning demonstrated improved oxygenation and a potential reduction in intubation rates in selected patients with ARDS receiving NIV or HFNC. Building on this experience, prone positioning can be considered a feasible and low-cost strategy in non-intubated ARDS patients, especially in resource-limited settings. Its success depends on careful patient selection, tolerance, and the availability of trained staff to ensure safety and effectiveness. Establishing clear protocols can help integrate this practice into standard supportive care.
15. Yes, but with significant risk for patients.
16. I strongly agree with this statement - there is strong supporting evidence base globally for this.
17. Personally, I believe that conscious proning is beneficial in many ARDS cases. However, we urgently need well-defined criteria for initiation, monitoring (what and how to monitor), and termination of awake proning. A dedicated, structured tool or protocol is essential to support clinical decisions, ensure timely escalation to intubation when needed, and standardize care delivery across different settings. We also require a tool (Like Proning Score) that utilizes an oxygenation index (either the PaO_2_/FiO_2_; ratio or the SpO_2_/FiO_2_ ratio) with a defined threshold value along with other parameters such as the WOB to trigger the initiation of prone positioning in conscious patients with ARDS.

**11.** In a selected group of patients with severe ARDS who are hemodynamically stable and show improvement during prone positioning, the intervention should be repeated as often as the patient continues to benefit, or until the PaO_2_/FiO_2_ ratio remains stable and exceeds 150 mmHg after at least 8 hours in the supine position.

| Status | Consensus | Agreement |
| --- | --- | --- |
| Consensus not achieved | No | 79.40% |

**Anonymous comments**

1. This recommendation promotes a tailored, responsive approach to the use of repeated prone positioning in severe ARDS. Continuation of proning should be guided by sustained clinical improvement—particularly in oxygenation—and hemodynamic stability. Notably, the PROSEVA trial (NEJM 2013) demonstrated a significant mortality benefit from early and prolonged prone sessions, even though a substantial proportion of patients were receiving norepinephrine, indicating that prone positioning can be safely performed in hemodynamically supported patients. Repeating the intervention until the PaO_2_/FiO_2_; ratio consistently exceeds 150 mmHg after at least 8 hours in the supine position ensures that prone sessions are used efficiently, balancing therapeutic benefit with the risks of prolonged intervention.
2. It depends on the etiology of hypoxemia. For instance, recruitment will never be conducted in severe hypoxemia in PE.
3. It should be repeated until the patient improves or a decision is made to withhold further treatment.
4. My concern on this statement is that the evidence for PP from PROSEVA is that it should be continued until the patient is ready to wean.
5. this depends on the patient, hemodynamics and suitability for prone position
6. If the expertise exists
7. No sense. High risk.
8. The evidence on optimal stopping criteria and the frequency of repeat prone sessions remains limited. Therefore, there is a clear need to develop a comprehensive proning protocol that addresses initiation and discontinuation criteria, safety measures, oxygenation targets, sedation management, ventilation monitoring, and the goals of lung-protective ventilation during prone positioning. We need to define as previously mentioned the hemodynamic stability we need to define the prone response. Assessment of the response to proning should not rely solely on the PaO_2_/FiO_2_; (P/F) ratio. A more comprehensive evaluation like using other variables like PEEP level, compliance, DP and radiological improvement). Using a fixed P/F ratio threshold, such as 150 mmHg, as the sole criterion for proning initiation or cessation can be misleading. P/F ratio should be interpreted in context alongside ventilator settings like PEEP, compliance, DP and other physiological variables. What about the permissive hypoxemia targets during the proning.
9. Collapsed or atelectatic areas of the lung can be re-expanded by the application of a brief period of high transpulmonary pressure followed by the application of adequate levels of PEEP to maintain the new aerated regions open.
10. I agree with this statement. However, since some patients may deteriorate due to hypotension or worsening compliance, careful patient selection, close monitoring, timely de-escalation, and an individualized titration approach should be emphasized when considering RMs.
11. “Until the PaO_2_/FiO_2_ ratio remains stable and exceeds 150 mmHg after at least 8 hours in the supine position.“ I think FiO_2_ should also be <60% and PEEP <10.
12. The side effects and harmful effects of resulting hemodynamic instability following RM should be monitored.
13. Check on the ventilator if the lungs are recruitable.
14. What do you mean by selected For me it is for all patients In Prilosecs the mean was 4 sessions.
15. And this is a very serious procedure. Maybe do a “little“ recruitment.

**Domain 4 - other supportive therapies**

**Statements**

**1.** Based on evidence gathered during the COVID-19 pandemic, corticosteroids such as dexamethasone (at doses below 20 mg) or equipotent doses of other corticosteroids should be considered in managing moderate to severe ARDS, provided there are no contraindications and ideally administered within the first 7 days of ARDS onset.

| Status | Consensus | Agreement |
| --- | --- | --- |
| Consensus not achieved | No | 76.38% |

**Anonymous comments**

1. No strong evidence in other ARDS.
2. There are multiple trials of steroids for ARDS currently in progress, a clear indication that the role of steroids in ARDS is not resolved. Steroids likely harm some patients, so a subphenotyping approach may be needed to optimize their therapeutic potential.
3. We do not have evidence to support this statement. Also, it depends on the etiology of ARDS.
4. early?
5. Only in moderate-severe and non-influenza.
6. According to the DEXA-ARDS trial, and several metanalysis, this recommendation is strong. Recent meta-analysis in patients with ARDS caused by pneumonia or sepsis, recommends the use of corticosteroids.
7. This recommendation is grounded in robust evidence from the COVID-19 pandemic, particularly the RECOVERY trial (NEJM 2021), which demonstrated a mortality benefit from low-dose dexamethasone in patients with moderate to severe ARDS requiring oxygen or mechanical ventilation. Administering corticosteroids ideally within the first 7 days of ARDS onset may help mitigate the inflammatory response and improve clinical outcomes when no contraindications exist. The emphasis on using dexamethasone at doses below 20 mg, or equivalent alternatives, reflects a balance between efficacy and minimizing potential adverse effects such as hyperglycemia, immunosuppression, or secondary infections. This approach aligns with current international guidelines and evolving best practices.
8. Yes, for limited time of exudative phase of ARDS (early phase 5-7 days).
9. In low-resource settings, where dexamethasone may not be readily available, alternative corticosteroids may be used. Therefore, clinical guidelines should clearly specify appropriate dosing for these alternatives to ensure effective and safe treatment.
10. The selection criteria and contraindications need to be more clear and defined. The recommended dose and duration should be clarified and implemented based on being COVID and non-COVID ARDS. No statement regarding elegibilty criteria for steriods in Influenza-Associated ARDS

**4.** In low-resource settings (LRS), the use of inhaled vasodilators is generally not recommended due to limited access to appropriate equipment and monitoring. However, short-term use may be considered as a life-saving intervention, particularly when all other options have been exhausted or during transport to a Level 3 care facility.

| Status | Consensus | Agreement |
| --- | --- | --- |
| Consensus not achieved | No | 75.74% |

**Anonymous comments**

1. While the intent of this recommendation is understandable, I do not find it practical in the context of low-resource settings. Inhaled vasodilators such as nitric oxide are extremely costly, require specialized delivery systems, and necessitate continuous monitoring—resources that are rarely available in most LRS environments. Given their limited impact on long-term outcomes and high operational demands, I believe prioritizing more feasible and cost-effective interventions is a more appropriate and sustainable approach for these settings.
2. Dependence on the clinical picture, the results of gas analyses.
3. There is no RCT evidence to support the use of inhaled vasodilators in any context - so the resource issue does not come into play.
4. Limited availability, costs, lack of trained personnel?
5. No evidence for this.
6. The ROSE trial was performed to reexamine the benefits of cisatracurium-induced paralysis in patients early after the onset of ARDS. Patients with moderate-to-severe ARDS were assigned either to a 48-hour continuous infusion of cisatracurium with deep sedation or to a usual-care approach with light sedation and without routine NMBA blockade. The results were markedly different from those of the ACURASYS trial.
7. These agents remain largely unavailable in such contexts.
8. Also, for patients with increased respiratory effort and this should be objectively measured in some way.
9. Totally agree. Restricting use to rescue therapy or transport is aligned with expert consensus that inhaled vasodilators are adjuncts with no proven mortality benefit, but may transiently improve oxygenation in severe ARDS or refractory hypoxemia.
10. I believe this statement is accurate, but in situations where there is a shortage of bedside healthcare personnel, it is sometimes impossible to ensure timely intermittent administration of neuromuscular blockade.
11. Agree.

**5.** Early tracheostomy, particularly percutaneous, may provide clinical benefits for patients with ARDS being treated in LRS, especially when performed within the first 10 - 14 days and in patients with ventilator parameters including FiO2 below 60% and PEEP below 8.

| Status | Consensus | Agreement |
| --- | --- | --- |
| Consensus not achieved | No | 73.87% |

**Anonymous comments**

1. There is no conclusive evidence that early tracheostomy, whether percutaneous or surgical, offers clear clinical benefits—even in patients managed in low-resource settings (LRSs).
2. This has not yet been proven unequivocally, especially in LRS where training may be inadequate
3. Tracheostomy can be done even later.
4. Dependence on the clinical picture, the results of gas analyses.
5. We do not have strong evidence to support this.
6. Not sure if 10-14 days is “early“ but it is appropriate.
7. With such parameters, shouldn’t we consider extubation?
8. I agree overall with this statement, but would note there is no evidence to support percutaneous over standard surgical tracheostomy. This may account for some of the disagreement.
9. I recommend that NMBA not be used routinely in patients with moderate-to-severe ARDS. There is a rationale to consider neuromuscular blocking agents in any patient with ARDS who, despite carefully implemented ventilatory and sedation strategies, has a ventilatory pattern that confers a predisposition to VILI. NMBA may also be considered in patients with increased respiratory drive that could generate potentially injurious transpulmonary pressure swings. NMBA can sometimes be used, when physiologically and clinically indicated.
10. This recommendation aligns with current evidence suggesting that early percutaneous tracheostomy typically within 10 to 14 days of mechanical ventilation may offer benefits in selected ARDS patients. Compared to surgical tracheostomy, the percutaneous approach is less invasive, can be performed at the bedside, and is associated with reduced procedure time, lower infection rates, and decreased bleeding risk when done by trained personnel. Studies such as the TracMan trial (JAMA, 2013) and subsequent meta-analyses suggest early tracheostomy may reduce sedation requirements and facilitate weaning, though survival benefits remain uncertain. In low-resource settings (LRS), percutaneous tracheostomy is particularly valuable due to its cost-effectiveness, avoidance of operating room use, and ability to free up ICU resources when applied to patients with improving oxygenation (FiO2 <60%, PEEP <8 cm H2O). Proper training and patient selection are essential to ensure safety and efficacy.
11. The term early should be defined. In the literature the early tracheostomy is defined as less than 7 days. The clinical benefits should be also defined such as sedation weaning, oral care, and mobility, and survival benefit for example FiO2; and PEEP thresholds should not serve as absolute criteria for initiating tracheostomy, as patients may remain at risk for progression of ARDS. Instead, the decision to proceed with tracheostomy should be individualized, taking into account the broader clinical context, including trends in respiratory mechanics, gas exchange, and the overall trajectory of the disease.
12. It facilitates better oral hygiene and adds to patients' comfort. Other than this, there is no data.

**Domain 7 – Nutrition**

**Statements**

**5**. When commercial feeds are unavailable, hospitals should use safe local ingredients to prepare blenderized enteral formulas with adequate nutrient content and a known caloric value. The constitution of these blenderized feeds should be done in consultation with a clinical dietitian to ensure proper nutrient composition and safety.

| Status | Consensus | Agreement |
| --- | --- | --- |
| Consensus not achieved | No | 60% |

**Anonymous comments**

1. Totally agree.
2. This seems a statement for which its not possible to get supportive evidence.
3. “may use“ rather than “should use“.
4. I agree with this last statement.
5. Efforts should also be made to improve both the microbiological safety and the overall nutritional quality of locally prepared enteral formulas. Attention should be given to appropriate methods of preparation, handling, and storage to minimize the risk of contamination and nutrient degradation. Proper conservation techniques are essential to ensure the safety and effectiveness of these feeds, particularly in settings without access to commercial nutritional products.
6. We can be considered.
7. This recommendation provides a realistic and necessary solution for settings where commercial enteral formulas are unavailable. Blenderized feeds using local, safe ingredients can effectively meet nutritional needs when prepared with attention to hygiene and nutrient content. Involving a clinical dietitian is essential to ensure the formulation provides adequate calories, protein, and micronutrients while maintaining safety and consistency. Establishing standardized recipes with known caloric values can support better monitoring and reduce the risk of malnutrition or contamination.

# ROUND 3

## Domain 3 - Treatment – respiratory support

Statements

**1.** Based on the experience gained during the COVID-19 pandemic, using prone positioning in awake ARDS patients receiving non-invasive ventilatory support (NIV/HFNC) can be considered as part of treatment. This recommendation mainly relies on evidence that, in selected patients - when sufficient staffing, proper training, patient safety, and patient tolerance are ensured - awake prone positioning can enhance oxygenation.

| Status | Consensus | Agreement |
| --- | --- | --- |
| Consensus not achieved | No | 70.76% |

**Anonymous comments**

1. We need pragmatic RCT and large observational studies to state those issues. The COVID-19 pandemic was a special situation. Now, we are dealing with patients with ARDS from many etiologies.
2. The recommendation is reasonable and supported by current evidence, particularly when implemented in structured protocols with clear criteria for continuation or escalation. Awake prone positioning represents a low-cost, low-risk, physiologically sound intervention that can enhance oxygenation and potentially reduce intubation rates in selected patients with ARDS on HFNC or NIV.
3. Even though it help, the processing need staffing and is challenging.
4. Can be helpful, but wrought with potential complications and demanding sedation while unable to properly evaluate the depth of sedation, potential asphyxia or vomiting due to it being out of view.
5. APP improved and intubation rates without improvement of survival.
6. But no evidence in non-COVID-19 ARDS patients!
7. Yes, I agree.
8. This was practiced widely during the COVID pandemic in our ICU. It was simple and without a significant increase in workload. There were no safety issues, and a positive effect was generally experienced. We did allow changes in position according patient needs but encouraged the position. Our experience was that it was easier among patients on HFNC compared to NIV where we often accepted the supine position at 30 -45 degrees head up. These were all hemodynamically stable patients.
9. Large scale trial and individual patient meta-analysis support this recommendation.
10. The most important thing is that the patient cooperates and knows what is being done to him.
11. proper monitoring when patient is on NIV is necessary with scoring such as HACOR to detect early deterioration.
12. This was quite helpful in our unit.
13. Awake prone positioning in patients receiving NIV or HFNC has been shown to improve oxygenation and may reduce the need for intubation when used in carefully selected patients. that was supported by literature from COVID (*Lancet Respir Med 2021 Dec;9(12):1387-1395*)
14. We noticed sudden improvement of saturation in some patient after prone position.

**2.** In a selected group of patients with severe ARDS who are hemodynamically stable and show improvement during prone positioning, the intervention should be repeated, considering the risk/benefit ratio. This procedure should continue as long as there are signs of clinical improvement (such as a PaO_2_/FiO_2_ ratio above 150, reduction in PEEP levels, improved compliance, radiological improvement) or until a decision is made to withhold further treatment.

| Status | Consensus | Agreement |
| --- | --- | --- |
| Consensus not achieved | No | 78.98% |

**Anonymous comments**

1. Totally agree. As in patients with severe ARDS who are hemodynamically stable and demonstrated a response to prone positioning, repeating the intervention is appropriate, provided the risk–benefit profile remains favorable and objective markers of improvement persist. Discontinuation should be based on lack of efficacy or clinical futility.
2. I agree, I support it.
3. If they show improvement better to use it if all perquisites fulfilled.
4. Please, the PROSEVA trial MUST BE VALIDATED with an external validation. In the last 10 years, we have learnt a lot about lung-protective ventilation, and the 28-d mortality rate reported in the PROSEVA trial (16%) is UNSEEN in any study published since then. The ROSE trial rejected the conclusions of the ACCURACYS trial.
5. Clarification here - proning should be continued even if no clear improvement in P/F ratio. Should only be stopped if a complication arises Important to continue until sustained clinical improvement to extent that permits weaning.
6. I agree, I support it.
7. If it turns out that it gives a good result, we should continue with such a procedure.
8. Again, with close monitoring. This is an essential treatment method especially in RLS as it can avoid intubation. It can also buy time for preparing for intubation.
9. This was our general experience as outlined previously. Patients did change position intermittently for comfort, and we generally accepted this as long as the patient went back into the prone position. In some cases, we had to encourage patients to re-prone as saturations decreased with prolonged periods in the supine position.
10. This recommendation is supported by multiple meta-analyses demonstrating that prone positioning in severe ARDS improves oxygenation and reduces mortality, particularly when applied early and in prolonged sessions. Evidence from studies such as those by Beitler et al. (2014), Park et al. (2015), Munshi et al. (2017), and Deng et al. (2023) reinforces the clinical benefit of repeating prone positioning in patients who show sustained improvement.

## Domain 4 - Other supportive therapies

**Statements**

1. Percutaneous tracheostomy, when performed within the first 10-14 days, may offer clinical benefits for patients with ARDS being treated in low-resource settings (LRS). It may reduce the need for sedation and facilitate weaning in patients who have previously failed extubation.

| Status | Consensus | Agreement |
| --- | --- | --- |
| Consensus not achieved | No | 73.32% |

**Anonymous comments**

1. I would add bronchoscope guidance during PT as obligatory.
2. As far as I know, no RCT has reported that a tracheostomy <10 days is not beneficial.
3. The statement reflects a reasonable clinical consideration, particularly in LRS. So in selected ARDS patients in LRS, percutaneous tracheostomy at 10–14 days may provide clinical benefits such as reduced sedation needs and facilitated weaning, particularly in the context of failed extubation or prolonged ventilation, provided adequate procedural safety is ensured.
4. The assumption that percutaneous tracheostomy expertise and logistics are readily available and sustainable in LRS is probably wrong,
5. It makes sense, but no clear evidence supporting this in the literature.
6. No strong evidence of 10-14 days, may be beyond that.
7. I have no much experience on this. We usually to surgical tracheotomy by general surgeons as there is no ENT surgeons in our set up. The few tracheostomies we did were converted to surgical tracheotomy the following days.
8. Agree - caveat is the training requirement for performing perc trachys
9. we have good experiences with this approach.
10. Early tracheostomy helps for weaning and prevent from ETT blockage
11. This is our general experience especially since theatre time is limited due to high demand. Delays waiting for open tracheostomies are commonplace. We generally have semi-open or percutaneous procedures done depending on equipment and consumable availability.
12. Percutaneous tracheostomy is feasible in low-resource settings as it can be safely performed at the bedside without the need for an operating room or surgical team. With basic equipment, proper training, and adherence to protocols, it offers a cost-effective and practical alternative to surgical tracheostomy, reducing both procedural burden and patient transport risks
13. I would add bronchoscope guidance during PT as obligatory.
14. This procedure certainly helps patients adapt better to mechanical ventilation and facilitates weaning from the machine.
15. Provided the patient is not on very high FiO_2_ or PEEP. there could be sudden cardio respiratory arrest during periods of no ventilation or disconnection. hence to be tried only in patients on low FiO_2_ and PEEP
16. I would say even earlier if expected time of intubation is more than 15 days.

## Domain 7 – Nutrition

**Statements**

1. When commercial feeds are unavailable, hospitals may use safe, local ingredients to prepare blenderized enteral formulas. In such cases, it is essential to involve a clinical dietitian to ensure that the formulas are prepared, stored, and handled appropriately to provide adequate energy, protein, and micronutrient intake, while maintaining microbiological safety and consistency. Developing standardized recipes with known caloric values can improve monitoring and help reduce the risk of malnutrition or contamination.

| Status | Consensus | Agreement |
| --- | --- | --- |
| Consensus not achieved | No | 67.80% |

**Anonymous comments**

1. Use of hospital-prepared blenderized enteral formulas is strongly discouraged by professional societies. There is a risk of infection, as well as variations in osmolarity, caloric, and protein content.
2. The statement is well-grounded and aligns with best practices for enteral nutrition in LRS or during supply shortages. It highlights the essential role of clinical dietitians when commercial enteral formulas are unavailable, emphasizing that hospital-prepared blenderized feeds are a viable interim option, but only when formulated and overseen by a dietitian to ensure nutritional adequacy, hygiene, and patient-specific customization.
3. Since commercial feeds are expensive even if it is available, using locally made nutrients help in feeding of critical ill patients.
4. Here there are too many assertions Is it the fact to use blenderized formulas or and to have a dietitian or both. In LRS I think the use of blenderized is very risky…. Having a dietitian who can manage all of that is a well challenging but might not be realistic. In my opinion it is not specific for ARDS and should be removed.
5. I am not sure, this is a good way. Enteral nutrition and pharmaco-nutrition by nutrition industry is better and more safe.
6. In the end I think it's cheaper to just buy commercial feeds.
7. Prepared nutrition formulas is not the problem. Whether a dietitian should be a mandatory personnel in LRS is a complex issue.
8. It is very difficult to comment on this as we have no experience.
9. Not an evidence-based recommendation, but is a pragmatic and logical approach.
10. The person administering or preparing the feeds should do the same in a very sterile manner. care should be taken to maintain appropriate osmolarity and the exact calories and proteins should be known.
11. The statement is well-grounded and aligns with best practices for enteral nutrition in LRS or during supply shortages. It highlights the essential role of clinical dietitians when commercial enteral formulas are unavailable, emphasizing that hospital-prepared blenderized feeds are a viable interim option, but only when formulated and overseen by a dietitian to ensure nutritional adequacy, hygiene, and patient-specific customization.
12. Most LRS do not have commercial feeds or even if available are very expensive and so the above statement is very true.

# e-Table 9. ACCORD (ACurate Consensus Reporting Document) checklist for reporting Delphi studies

|  | Section | Checklist Item (*help text*) | Page No. |
| --- | --- | --- | --- |
| T1 | **Title** | Identify the article as reporting a consensus exercise and state the consensus methods used in the title.  *For example, Delphi or nominal group technique.* | 1 |
| I1 | **Introduction** | Explain why a consensus exercise was chosen over other approaches. | 5,6 |
| I2 |  | State the aim of the consensus exercise, including its intended audience and geographical scope (national, regional, global). | 5,6 |
| I3 |  | If the consensus exercise is an update of an existing document, state why an update is needed, and provide the citation for the original document. | 5,6 |
| M1 | **Methods**  Registration | If the study or study protocol was prospectively registered, state the registration platform and provide a link. If the exercise was not registered, this should be stated.  *Recommended to include the date of registration.* | Methods, page 9: "This Delphi study was not prospectively registered." |
| M2 | Selection of SC and/or panellists | Describe the role(s) and areas of expertise or experience of those directing the consensus exercise.  *For example, whether the project was led by a chair, co-chairs or a steering committee, and, if so, how they were chosen. List their names if appropriate, and whether there were any subgroups for individual steps in the process.* | 3,8 |
| M3 |  | Explain the criteria for panellist inclusion and the rationale for panellist numbers. State who was responsible for panellist selection. | 8,9 |
| M4 |  | Describe the recruitment process (how panellists were invited to participate).  *Include communication/advertisement method(s) and locations, numbers of invitations sent, and whether there was centralised oversight of invitations or if panellists were asked/allowed to suggest other members of the panel.* | 9 |
| M5 |  | Describe the role of any members of the public, patients or carers in the different steps of the study. | N/A |
| M6 | Preparatory research | Describe how information was obtained prior to generating items or other materials used during the consensus exercise.  *This might include a literature review, interviews, surveys, or another process.* | e-Supplement 2 |
| M7 |  | Describe any systematic literature search in detail, including the search strategy and dates of search or the citation if published already.  *Provide the details suggested by the reporting guideline PRISMA and the related PRISMA-Search extension.* | e-Supplement 2 |
| M8 |  | Describe how any existing scientific evidence was summarised and if this evidence was provided to the panellists. | e-Supplement 2 |
| M9 | Assessing consensus | Describe the methods used and steps taken to gather panellist input and reach consensus (for example, Delphi, RAND-UCLA, nominal group technique).  *If modifications were made to the method in its original form, provide a detailed explanation of how the method was adjusted and why this was necessary for the purpose of your consensus-based study.* | 9,10 |
| M10 |  | Describe how each question or statement was presented and the response options. State whether panellists were able to or required to explain their responses, and whether they could propose new items.  *Where possible, present the questionnaire or list of statements as supplementary material.* | 9,10 |
| M11 |  | State the objective of each consensus step.  *A step could be a consensus meeting, a discussion or interview session, or a Delphi round.* | 9,10 |
| M12 |  | State the definition of consensus (for example, number, percentage, or categorical rating, such as ‘agree’ or ‘strongly agree’) and explain the rationale for that definition. | 9,10 |
| M13 |  | State whether items that met the prespecified definition of consensus were included in any subsequent voting rounds. | 9,10 |
| M14 |  | For each step, describe how responses were collected, and whether responses were collected in a group setting or individually. | 9,10 |
| M15 |  | Describe how responses were processed and/or synthesised.  *Include qualitative analyses of free-text responses (for example, thematic, content or cluster analysis) and/or quantitative analytical methods, if used.* | e-Supplement 1 |
| M16 |  | Describe any piloting of the study materials and/or survey instruments.  *Include how many individuals piloted the study materials, the rationale for the selection of those individuals, any changes made as a result and whether their responses were used in the calculation of the final consensus. If no pilot was conducted, this should be stated.* | .3 |
| M17 |  | If applicable, describe how feedback was provided to panellists at the end of each consensus step or meeting.  *State whether feedback was quantitative (for example, approval rates per topic/item) and/or qualitative (for example, comments, or lists of approved items), and whether it was anonymised.* | 10 |
| M18 |  | State whether anonymity was planned in the study design. Explain where and to whom it was applied and what methods were used to guarantee anonymity. | 9 |
| M19 |  | State if the steering committee was involved in the decisions made by the consensus panel.  *For example, whether the steering committee or those managing consensus also had voting rights.* | 3 |
| M20 | Participation | Describe any incentives used to encourage responses or participation in the consensus process.  *For example, were invitations to participate reiterated, or were participants reimbursed for their time.* | N/A |
| M21 |  | Describe any adaptations to make the surveys/meetings more accessible.  *For example, the languages in which the surveys/meetings were conducted and whether translations or plain language summaries were available*. | N/A |
| R1 | Results | State when the consensus exercise was conducted. List the date of initiation and the time taken to complete each consensus step, analysis, and any extensions or delays in the analysis. | 9 |
| R2 |  | Explain any deviations from the study protocol, and why these were necessary.  *For example, addition of panel members during the exercise, number of consensus steps, stopping criteria; report the step(s) in which this occurred.* | N/A |
| R3 |  | For each step, report quantitative (number of panellists, response rate) and qualitative (relevant socio-demographics) data to describe the participating panellists. | e-Supplement 1 |
| R4 |  | Report the final outcome of the consensus process as qualitative (for example, aggregated themes from comments) and/or quantitative (for example, summary statistics, score means, medians and/or ranges) data. | Tables 1–3 and the supplementary files. |
| R5 |  | List any items or topics that were modified or removed during the consensus process. Include why and when in the process they were modified or removed. | 11,12 |
| D1 | Discussion | Discuss the methodological strengths and limitations of the consensus exercise.  *Include factors that may have impacted the decisions (for example, response rates, representativeness of the panel, potential for feedback during consensus to bias responses, potential impact of any non-anonymised interactions).* | 15 |
| D2 |  | Discuss whether the recommendations are consistent with any pre-existing literature and, if not, propose reasons why this process may have arrived at alternative conclusions. | 13-15 |
| O1 | Other information | List any endorsing organisations involved and their role. | N/A |
| O2 |  | State any potential conflicts of interests, including among those directing the consensus study and panellists. Describe how conflicts of interest were managed. | 3 |
| O3 |  | State any funding received and the role of the funder.  *Specify, for example, any funder involvement in the study concept/design, participation in the steering committee, conducting the consensus process, funding of any medical writing support. This could be disclosed in the methods or in the relevant transparency section of the manuscript. Where a funder did not play a role in the process or influence the decisions reached, this should be specified.* | 3 |

From: PLoS Med 21(1): e1004326. <https://doi.org/10.1371/journal.pmed.1004326> For more information see: <https://www.ismpp.org/accord>

# RESEARCH IN CONTEXT

**Evidence before this study**

On January 1, 2025, we started a search in PubMed, Embase, Web of Science, and Google Scholar for studies published in English from Jan 1, 2010, to April 30, 2025, using the terms (“acute respiratory distress syndrome” OR “ARDS”) AND (“low-resource settings” OR “resource-limited settings” OR “low- and middle-income countries” OR “developing countries”) AND (“management” OR “guidelines” OR “critical care” OR “Delphi”). Evidence from HICs consistently shows that interventions such as low-tidal volume ventilation, prone positioning, neuromuscular blockade, and conservative fluid strategies improve outcomes in well-resourced ICUs. However, most ARDS research originated from health systems with advanced diagnostics, reliable oxygen delivery, and adequate staffing. Studies from LRS reported considerably higher mortality and important barriers for applying international guidelines, including shortages of oxygen, limited monitoring, insufficient trained personnel, and infrastructural constraints. No prior study has systematically developed consensus-based ARDS management statements tailored to these settings. Additionally, expert-driven guidance on diagnostic tools, sedation, liberation from mechanical ventilation (weaning), nutrition, and complication prevention in LRS is largely absent. No previous Delphi exercise has generated a global multidisciplinary consensus, specifically focused on ARDS management in LRS.

Identification of studies via databases and registers

- PubMed,

- Embase,

- Web of Science and

- Google Scholar

Jan 1, 2010, to April 30, 2025

Screening

65 references were identified through screening

15 were excluded

Included

50 references were included in the study

# e-Figure 11. Prisma Flowchart of studies included for Qualitative Evidence Synthesis

# e-Table 10. Final studies included for drafting statements of the Delphi rounds

| **First author, year of publication** | **Type of study** | **Country** | **Number of patients (articles)** | **Intervention** | **Comparator** | **Main findings** | **Relevant domains** |
| --- | --- | --- | --- | --- | --- | --- | --- |
| Ma W. 2025. (1) | Narrative review | Upper-Middle Income Country, MIC | N/A (review article) | Lung-protective ventilation (low tidal volume 4-8 ml/kg, Pplat <30 cmH2O), prone positioning (12-16h/day for moderate-severe ARDS), mesenchymal stem cells (MSCs), C5aR1 antagonists, high PEEP in recruitable lungs, ECMO for refractory cases | Standard ventilation (higher tidal volumes), supine positioning, placebo/usual care in trials (e.g., MSCs vs. placebo; dexamethasone vs. usual care showing mortality benefit in early ARDS). | ARDS exhibits heterogeneity (subphenotypes: hyperinflammatory/hypo-inflammatory), pathophysiology (alveolar injury, immunothrombosis, NETs, endothelial barrier via NOX4/CaMKII, METTL3-m6A pathways), and therapies (MSCs for immunomodulation, complement blockade C5aR1, personalized ventilation by subphenotypes; prone positioning beneficial in exudative phase) | 1,3,4 |
| Ferguson ND, 2012. (2) | Consensus statement/review with expanded rationale | Canada/International (High-Income Country, HIC; multinational panel). | N/A (consensus study) | N/A (definitional; supports low tidal volume ventilation, PEEP titration). | Original AECC definition (lacking PEEP/O2 requirements, poor mild ARDS separation); cardiogenic edema exclusion via echo/PCWP | Provides detailed justification for Berlin ARDS criteria (mild/moderate/severe based on PaO2/FiO2: 200-300/≤200/≤100 mmHg with PEEP≥5 cmH2O), emphasizing acute onset (<1 week), bilateral opacities, non-cardiogenic edema; improves prognostic stratification over AECC (mortality 27%/31%/45%) and guides trial enrollment. | 3 |
| Sadana D., 2023. (3) | Systematic review and meta-analysis | Australia (High-Income Country, HIC) | 24,158 patients across 102 studies (34 RCTs, 68 observational). | N/A (meta-analysis of varied ARDS management; reflects real-world lung-protective ventilation era) | N/A (pooled observational/RCT data; higher mortality in observational vs interventional trials) | Pooled ARDS mortality rate 2009-2019 was 39.4% (95% CI 37.0-41.8%); higher in observational studies (41.8%) vs RCTs (34.5%); minimal temporal decline post-2009 H1N1, stabilizing around 39%; calls for phenotype-specific reporting. | 3 |
| Kovacevic P., 2020. (4) | Retrospective observational cohort study | Western Balkans/Bosnia and Serbia (Upper-Middle Income Country, MIC) | 43 critically ill patients | Oseltamivir (antiviral therapy), mechanical ventilation (invasive/non-invasive), prone positioning, ECMO in refractory cases, supportive ICU care (fluids, vasopressors). | N/A (single-arm observational; subgroups by oseltamivir timing, ventilation type, comorbidities) | High mortality (37.2%) among H1N1 critically ill patients in 2019 post-pandemic season; delayed oseltamivir (>48h) associated with worse outcomes; bacterial coinfections common; mechanical ventilation required in 88%; early antivirals and lung-protective strategies improved survival in this MIC setting. | 3,4 |
| Čučković M., 2022. (5) | Retrospective observational cohort study | Croatia (High-Income Country, HIC) | 692 critically ill COVID-19 patients | High-flow nasal oxygen (HFNO, 61.8%), mechanical ventilation (MV, 80.5%), supportive ICU care; median HFNO 3 days, MV 7 days | Survivors vs non-survivors (subgroup analysis by age, comorbidities, lab parameters, ventilation status) | Median 3 comorbidities, Charlson Comorbidity Index 5; high ICU burden with 67.1% men; survivors vs non-survivors differed by age, CRP, D-dimer, lactate; mortality predictors align with global data; calls for multivariate risk factor analysis in Croatian COVID-19 cohort | 3,4 |
| Sharma S., 2023., (6) | Editorial/commentary | Australia/Nepal (High-Income Country, HIC; authors from LMICs represented) | N/A (editorial; references global data, e.g., low back pain research gaps in LMICs) | N/A (advocacy for research support: funding, mentorship, journal fee waivers, EDI policies for LMIC physiotherapy research). | N/A (contrasts HIC vs LMIC research output: LMICs 85% world population but minimal publications). | LMIC physiotherapy research underrepresented despite high disability burden; barriers include funding lack, weak research culture, journal biases; calls for journals/editors to waive fees, mentor LMIC authors, prioritize local priorities for global health equity. | 1 |
| Kovacevic P., 2023., (7) | Letter/commentary/editorial | Bosnia and Herzegovina (Upper-Middle Income Country, MIC) | N/A (conceptual discussion; no primary data) | N/A (advocacy for precise terminology in ICU research) | "Low resource setting" (LRS: focuses on infrastructure/equipment limitations, can occur in any income level) vs "low-income country" (LIC: World Bank GDP-based classification). |  | 1 |
| Kwizera A ., 2023. (8) | Multicenter prospective observational study | Uganda (Low-Income Country, LIC) | 499 patients with COVID-19-associated ARDS across 13 hospitals | Standard oxygen (SOX), high-flow nasal oxygen (HFNO), CPAP, non-invasive ventilation (NIV), invasive mechanical ventilation (IMV) | Non-invasive (HFNO/CPAP/NIV) vs invasive mechanical ventilation; SOX vs advanced respiratory support | NIV/HFNO used in 40%, IMV in 25%; overall mortality 52%; IMV associated with higher mortality (68%) vs NIV success (42%); resource constraints limited advanced care; early NIV reduced intubation need in Sub-Saharan African ICUs. | 3, 4 |
| Kovacevic P., 2022., (9) | Single-center experience report/review | Bosnia and Herzegovina (Upper-Middle Income Country, MIC) | N/A (programmatic description; covers 10-year development treating hundreds of patients). | Multidisciplinary critical care training (Mayo Clinic mentorship), lung-protective ventilation, noninvasive/invasive ventilation, ECMO, CRRT, targeted temperature management; staff education, FCCS courses, subspecialty curriculum. | Pre-implementation (limited MICU capacity, high mortality) vs post-development (expanded 24-bed level III MICU with full organ support) | Successful MICU establishment in LMIC via HIC mentorship, local authority support, multidisciplinary training; grew from 8 to 24 beds with vvECMO/iNO/CRRT; ISO-certified regional referral center; model emphasizes trained staff over equipment, rapid COVID-19 expansion to regional hospitals. | 1,4,5 |
| Inglis R., 2019., (10) | Narrative review | International/LMIC-focused (United Kingdom authors, global LMIC context) | N/A (review; synthesizes data from multiple observational studies and trials in resource-limited settings) | Low-cost oxygen delivery (concentrators, nasal prongs), non-invasive ventilation (CPAP/BiPAP with improvised circuits), lung-protective mechanical ventilation protocols adapted for basic ventilators, prone positioning, training for non-specialist staff | High-resource standard care (advanced ventilators, ECMO) vs resource-adapted strategies (manual ventilation, helmet NIV vs standard masks) | Respiratory support in LMICs feasible with simplified protocols; oxygen therapy reduces mortality by 30-50% in pneumonia; NIV/CPAP effective alternative to intubation (success rates 60-80%); emphasizes staff training, equipment maintenance, and avoiding over-reliance on scarce advanced tech for better outcomes in acute respiratory failure. | 1, 2, 3, 4, 5 |
| Grasselli G., 2023., (11) | Clinical practice guideline (ESICM Taskforce consensus) | Europe/International (High-Income Countries, HIC) | N/A (guideline; based on systematic reviews of >100 RCTs and observational studies) | Lung-protective ventilation (low tidal volume 4-8 mL/kg PBW, Pplat ≤30 cmH2O), higher PEEP in moderate-severe ARDS (PEEP-FiO2 tables), prone positioning ≥12h/day (strong recommendation), neuromuscular blockade (early cisatracurium), conservative fluid strategy post-exudative phase, HFNC/NIV in mild ARDS or post-extubation | Low vs higher PEEP (recruitment-guided), prone vs supine positioning, neuromuscular blockade vs placebo, liberal vs conservative fluids (FACTT trial), HFNC/NIV/CPAP vs standard oxygen. | Updates 2017 guidelines with strong recommendations for prone positioning (mortality reduction in moderate-severe ARDS), higher PEEP titration, early neuromuscular blockade; weak for ECMO (rescue), corticosteroids (moderate evidence); emphasizes ARDS phenotyping (Berlin+ subphenotypes, ultrasound/CT for diagnosis); no pharmacologic cures, focus on ventilator strategies reducing VILI | 1,2,3,4,5,6 |
| Serpa Neto A., 2021., (12) | Pragmatic guideline/consensus recommendations | International/LMIC-focused (MORU Thailand, global task force) | N/A (guideline; synthesizes evidence from early COVID-19 observational data and RCTs in resource-limited contexts | Lung-protective ventilation (tidal volume 4-6 mL/kg PBW, plateau pressure ≤30 cmH2O), prone positioning (even awake patients), high-flow nasal cannula (HFNC) or CPAP for mild-moderate hypoxemia, early dexamethasone (6 mg/day for 10 days if ventilated), simplified sedation protocols, oxygen concentrators/manual ventilation backups | Standard/higher tidal volume ventilation, supine positioning, low-flow oxygen/conventional masks vs HFNC/CPAP, no steroids vs dexamethasone (RECOVERY trial adaptation). | Resource-adapted ARDS/COVID-19 management feasible in LMICs using basic ventilators and non-invasive strategies; prone positioning reduces mortality (12-16h/day); early steroids improve survival in ventilated patients; emphasizes training non-ICU staff, avoiding over-sedation/intubation, and local protocols over HIC standards for better outcomes in low-resource ICUs. | 1,2,3,4,5,6 |
| Duarte PAD, 2022., (13) | Commentary/letter (call to action | Brazil (Upper-Middle Income Country, MIC) | N/A (editorial; references global MIC data on ventilated patients). | N/A (advocacy for improved mechanical ventilation access, training, and protocols in MICs) | MIC ventilated patient mortality (50-70%) vs HIC (30-40%); COVID-19 era disparities. | Mechanically ventilated patients in MICs face 2x higher mortality than HICs due to resource gaps, delayed care, and suboptimal protocols; COVID-19 exposed vulnerabilities; urgent need for MIC-specific trials, training, affordable ventilators, and global equity in ICU research/practice | 1,3,4 |
| Matthay MA, 2021., (14) | Commentary/proposal (viewpoint article) | USA (High-Income Country, HIC) | N/A (conceptual; references trials like Frat et al. HFNO study with ~800 patients) | N/A (discussion on diagnostic criteria expansion) | Current Berlin definition (requires PEEP/CPAP ≥5 cmH2O via mask/ventilation) vs proposed broadened definition including HFNO ≥30 L/min | Berlin ARDS definition should expand to include HFNO patients (≥30 L/min) meeting other criteria (acute onset, bilateral opacities, PaO2/FiO2 ≤300, non-cardiogenic); enables earlier ARDS recognition, trial enrollment, and resource-limited settings (Kigali modification alignment); addresses COVID-19 hypoxemia treated without intubation; calls for consensus on SpO2/FiO2 ≤315 integration | 1,2,3,4 |
| Ranieri VM, 2023., (15) | Viewpoint/commentary | Italy/International (High-Income Country, HIC) | N/A (conceptual; references COVID-19 ARDS datasets and Berlin definition trials) | N/A (discussion on ARDS diagnostic framework post-COVID) | Berlin definition (PEEP ≥5 cmH2O requirement) vs proposed global definition (including HFNC/NIV without PEEP, SpO2/FiO2); COVID-19 ARDS vs classical ARDS phenotypes | COVID-19 ARDS crisis reveals Berlin definition limitations (excludes early/mild cases on HFNC, hinders global applicability); advocates psychologist-developed framework prioritizing clinical utility over precision; calls for "better" definition answering specific questions (epidemiology, trials, resource allocation) rather than universal one; supports Kigali/global modifications for LMICs | 1,2,3,4,5 |
| Chebib N., 2018., (16) | Secondary analysis/economic perspective (LUNG SAFE dataset) | International (High/Middle-Income Countries, HIC/MIC) | 2,377 ARDS patients from LUNG SAFE study | N/A (observational economic analysis of ARDS care patterns) | High-income regions (rWORLD-High, Europe-High) vs Middle-income countries; evidence-based practices (low tidal volume ventilation, higher PEEP) vs usual care | RDS hospital mortality lower in high-GDP regions (adjusted OR 0.32 rWORLD-High, 0.51 Europe-High vs Middle); geo-economic disparities persist after adjustment; limited adherence to evidence-based ventilation explains outcome gaps; calls for global implementation strategies to reduce economic burden and improve equity. | 1,3,4 |
| Vukoja M., 2018., (17) | Narrative review | Serbia/International LMIC-focused (Upper-Middle Income Country, MIC) | N/A (review; synthesizes data from multiple LMIC ICU studies) | Task-shifting to nurses/non-physicians, protocolized care (sepsis bundles, lung-protective ventilation), low-cost monitoring (vital signs-directed protocols), quality improvement initiatives, early warning systems | HIC outcomes (lower mortality ~20-30%) vs LMIC (higher ~50-60%); resource-rich vs adapted interventions (e.g., advanced ventilators vs basic oxygen/NIV) | Critically ill patients in LMICs have higher mortality than HICs due to delayed recognition, limited resources, and poor adherence to evidence-based care; feasible improvements via training, checklists, and context-specific scores; calls for LMIC-focused epidemiology, severity scoring, and long-term outcome studies beyond in-hospital mortality. | 1,2,3,4, |
| Matthay MA, 2017., (18) | Narrative review | USA (High-Income Country, HIC) | N/A (review; discusses >50 failed ARDS trials like FACTT, ALVEOLI, ART) | Lung-protective ventilation (low tidal volume), higher vs lower PEEP, prone positioning, neuromuscular blockade (cisatracurium), conservative fluids (FACTT), statins (SAILS), omega-3 (OMEGA), beta-agonists (BETA-AGENT), corticosteroids, ECMO | Low vs high tidal volume (6 vs 12 mL/kg), higher vs lower PEEP tables, prone vs supine, cisatracurium vs placebo, liberal vs conservative fluids, active drugs vs placebo | ARDS trials fail due to heterogeneity (subphenotypes), small effect sizes (~5% mortality reduction), underpowered studies; successes limited to ventilation strategies; opportunities in biomarkers/ML for phenotyping, precision medicine, larger adaptive/platform trials targeting hyper/hypo-inflammatory subgroups | 1,3,4,5,6, |
| Cardiff University (institutional guide, no individual author), 2017., (19) | Methodological guide/handbook | United Kingdom (High-Income Country, HIC) | N/A (practical methodology manual) | N/A (step-by-step Delphi process: expert recruitment, round 1 open questions, controlled feedback, 2-3 rating rounds, stability/consensus criteria) | Ad-hoc expert consensus vs structured anonymous Delphi (reduces bias, improves stability tracking via medians/IQR) | Delphi achieves consensus through 2-4 iterative anonymous rounds with summarized feedback; key steps include panel selection (10-20 experts), consensus threshold (70-80% agreement or IQR<2), quasi-anonymity for accountability; addresses common pitfalls like attrition, scale design, and reporting transparency for reproducible health research | Delphy metodology |
| Calibrum (company/institutional resource, no individual author) (20) | N/A ( Software description/promotional material) | USA (High-Income Country, HIC; St. George, UT) | N/A (software tool; applications in clinical trials, guidelines, COS development). | N/A (Delphi platform features: real-time consensus building, multi-round surveys, live analytics with 30+ statistics like median/IQR, stakeholder feedback, anonymity). | Classic multi-round Delphi vs Real-Time Delphi (RTD with continuous feedback/snapshots); overcomes social bias in group decisions | Surveylet enables efficient group consensus via Delphi method for complex decisions (healthcare, policy, forecasting); supports core outcome sets, clinical guidelines, quality indicators; real-time stats accelerate agreement without undue influence; applicable to trials lacking RCTs by standardizing outcomes and prioritizing measures. | Delphi metodology |
| Ranieri VM, 2012., (21) | Consensus guideline | International (High-Income Countries, HIC) | N/A (consensus; validated on ~4,000 patients from prior cohorts) | N/A (diagnostic criteria development) | AECC definition (1994) vs Berlin (mild/moderate/severe ARDS by PaO2/FiO2: 200-300/100-200/≤100 mmHg with PEEP≥5 cmH2O). | Berlin definition improves reliability/validity over AECC; requires acute onset (<1 week), bilateral opacities, non-cardiogenic edema; mortality stratification 27%/32%/45%; feasible for trials/clinical use; excludes mild ARDS without PEEP. | 2,3,4 |
| Gattrell WT, 2024., (22) | Reporting guideline (developed via modified Delphi) | United Kingdom/International (High-Income Country, HIC) | N/A (guideline; based on systematic review of 234 consensus studies). | N/A (ACCORD checklist with 36 items across 10 domains for consensus reporting) | Poorly reported consensus studies vs ACCORD-compliant reporting (e.g., Delphi, nominal group, RAND/UCLA) | ACCORD is first universal reporting guideline for all biomedical consensus methods; addresses gaps in transparency (e.g., panel selection, consensus definition, attrition); improves reproducibility, reader interpretation, and applicability of recommendations when evidence limited/inconsistent; endorses EQUATOR registration | Delphy metodology |
| Gawda R., 2022., (23) | Feasibility and safety study (retrospective cohort) | Poland (High-Income Country, HIC) | 30 patients treated with ECMO | Veno-venous ECMO (VV-ECMO) using ICU-based multidisciplinary team (intensivists, anesthesiologists, perfusionists), mobile retrieval team, standardized protocols for cannulation/anticoagulation | Multidisciplinary (separate ECMO team) vs ICU-based approach (integrated within existing ICU structure) | ICU-based ECMO center feasible/safe with 70% survival to discharge; rapid setup (3 months) via training/simulations; lower costs/staff burden than multidisciplinary model; effective referral system (website/phone) with 30-min decisions; proves scalability in resource-constrained settings | 1,3,4 |
| Losonczy LI, 2021., (24) | White paper/policy recommendations | International/LMIC-focused (Low/Middle-Income Countries, LMIC) | N/A (policy document; synthesizes LMIC critical care data). | Early critical care bundles (oxygen, fluids, vasopressors), task-shifting to non-physicians, low-cost monitoring, pre-hospital EMS, integrated hospital models, nurse:patient ratios 1:4-6. | Fragmented care vs system-level early critical care (EU-ICU integration, mobile teams); HIC models vs LMIC-adapted approaches | LMICs face high critical illness burden/worse outcomes due to fragmented systems, workforce gaps, costs; early critical care cost-effective via education/research/policy pillars; no single "best" model—emphasize local context, triage, simple tools; policy needs include funding access, ethical withdrawal guidelines, broad stakeholder input. | 1 |
| Kovacevic P., 2024., (25) | Modified Delphi consensus study | International/LRS-focused (Bosnia and Herzegovina lead, MIC) | N/A (consensus; 43 experts from 20 countries). | N/A (consensus statements on MICU organization: multispecialty units, formal training, authority support) | Fragmented care vs structured medical ICUs; HIC models vs LRS-adapted (telemedicine, task-shifting) | 13 consensus statements (≥80% agreement) across organization/staffing/education; favors medical intensivist-led MICUs with multidisciplinary support; emphasizes health authority recognition, professional societies, HIC-trained educators, tele-education for sustainable LRS critical care post-COVID. | 1 |
| Carvalho EB, 2022., (26) | Narrative review | Brazil (Upper-Middle Income Country, MIC) | N/A (review; analyzes preclinical/clinical datasets) | N/A (evaluation of SpO2/FiO2 as PaO2/FiO2 surrogate in ARDS diagnosis/severity) | PaO2/FiO2 (gold standard, invasive) vs SpO2/FiO2 (non-invasive); varying FiO2, hemoglobin, PEEP levels, shock states | SpO2/FiO2 correlates moderately with PaO2/FiO2 (r=0.66-0.89) but limited by oxyhemoglobin dissociation curve shifts (acidosis, temperature, 2,3-DPG), FiO2>60% saturation plateau, anemia/hypoxemia inaccuracies; useful in resource-limited settings (S/F<235≈P/F<200 for mild ARDS) but not interchangeable without corrections; recommends validation per context. | 2,3 |
| Erlebach R., 2025., (27) | Observational cohort study | Switzerland/Germany (High-Income Country, HIC) | 708 ARDS patients | N/A (evaluation of SpO2/FiO2 for ARDS monitoring) | SpO2/FiO2 vs PaO2/FiO2 ratios; varying FiO2 levels | SpO2/FiO2 misclassifies ARDS severity in 33% of datapoints (84% overestimated); poor trending ability (<20% accurate progression tracking); highly FiO2-dependent (worst at 70% FiO2); limited surrogate due to SpO2 imprecision and transformation equations; cautions against interchangeable use for trials/treatment decisions. | 2,3 |
| Wick KD, 2022., (28) | Narrative review | USA (High-Income Country, HIC) | N/A (review; synthesizes data from multiple ARDS cohorts and trials). | N/A (pulse oximetry indices: SpO2/FiO2, OSI for ARDS diagnosis/monitoring) | PaO2/FiO2 (arterial blood gas) vs SpO2/FiO2 (pulse oximetry); continuous vs intermittent monitoring. | SpO2/FiO2 valid surrogate for PaO2/FiO2 (SpO2/FiO2 ≤235≈PaO2/FiO2 ≤200 for Berlin ARDS); enables earlier diagnosis, continuous monitoring, LMIC applicability; limitations include skin pigmentation bias, FiO2>60% inaccuracy, saturation plateau >97%; supports expanded ARDS criteria integration for global epidemiology and trials | 2,3 |
| Matthay MA, 2024., (29) | Consensus guideline (global expert conference) | International (High/Low/Middle-Income Countries, global) | N/A (definition; validated on prior ARDS cohorts) | N/A (expanded diagnostic criteria for ARDS) | Berlin definition (2012: requires PEEP≥5, CXR, PaO2/FiO2) vs Global definition (includes HFNC≥30 L/min, SpO2/FiO2≤315, lung ultrasound) | New Global ARDS definition incorporates HFNC, pulse oximetry (SpO2/FiO2), ultrasound imaging for resource-limited applicability; maintains acute onset, bilateral opacities, non-cardiogenic edema; enables earlier diagnosis, global epidemiology, trial enrollment without invasive monitoring; prognostic validation pending prospective studies | 1,2,3,4,5 |
| Bass CM, 2015., (30) | Prospective pilot observational study | USA (High-Income Country, HIC) | 100 ICU assessments (62 patients with respiratory failure) | N/A (diagnostic comparison study) | Pulmonary ultrasound + pulse oximetry (SpO2/FiO2 ≤315, UIS-3 bilateral) vs chest radiography + arterial blood gas (PaO2/FiO2 ≤300, bilateral opacities | Ultrasound + SpO2/FiO2 combination shows 83% sensitivity/62% specificity for ARDS oxygenation/radiographic criteria; SpO2/FiO2 ≤235 + UIS-3 specific (88%/69%) for moderate-severe ARDS; feasible bedside alternatives in resource-limited settings but moderate overlap suggests complementary use | 2,3,4 |
| Smit MR, 2024., (31) | Commentary/review | Netherlands (High-Income Country, HIC) | N/A (perspective on LUS evidence synthesis) | N/A (lung ultrasound protocols for ARDS diagnosis/phenotyping) | Chest X-ray/CT vs lung ultrasound (LUS) for bilateral opacities, focal/non-focal patterns. | LUS highly specific (94%) but moderately sensitive (63%) for ARDS per Berlin criteria; excels at subphenotyping (focal vs diffuse loss of aeration); bedside utility for dynamic monitoring, recruitment assessment, VILI avoidance; supports Global ARDS definition integration despite protocol heterogeneity. | 2 |
| Plantinga C., 2024., (32) | Observational cohort study | Netherlands (High-Income Country, HIC) | Not specified in available data (prospective ICU cohort) | N/A (diagnostic comparison using new ARDS definitions) | Traditional Berlin definition (CXR + PaO2/FiO2) vs updated definitions incorporating lung ultrasound (LUS) + SpO2/FiO2 | Incorporating lung ultrasound into new ARDS definitions significantly increases ARDS occurrence rate (detection rather than true incidence); LUS identifies more bilateral abnormalities than CXR; supports Global definition utility for earlier diagnosis but highlights potential overdiagnosis risk without validation | 2,3,4 |
| Riviello ED, 2016., (33) | Prospective observational cohort study | Rwanda (Low-Income Country, LIC) | 487 hospitalized adults screened, 118 with ARDS by Kigali modification | N/A (epidemiological assessment using modified diagnostic criteria) | Berlin definition (requires PEEP≥5 cmH2O, PaO2/FiO2) vs Kigali modification (SpO2/FiO2≤315, no PEEP requirement, CXR/lung US). | Hospital ARDS incidence 18.7/1000 using Kigali modification (vs Berlin underestimation in LMICs); 64% mortality; validates SpO2/FiO2 and imaging alternatives for resource-limited diagnosis; captures untreated/mild cases missed by Berlin criteria. | 2 |
| Vukoja M, 2021., (34) | Multicenter quality-improvement study (before-after) | International (variable resources, HIC/LMIC ICUs) | 5,942 ICU admissions across 34 ICUs (15 countries) | CERTAIN checklist (web-based decision support for admission/rounding: ABCDE survey, evidence-based prompts for DVT prophylaxis, sedation, weaning, catheters) | Pre-implementation (usual care) vs post-CERTAIN (structured checklist use after remote training). | CERTAIN reduced nonadherence to processes (e.g., DVT prophylaxis 26% improvement, sedation assessment 38%); associated with shorter ICU LOS (14% reduction), hospital LOS (8%), and mortality (19% OR reduction); feasible across resource-variable ICUs via train-the-trainer model. | 4,5,6 |
| Kovačević P., 2019., (35) | Before-after quality improvement study | Bosnia and Herzegovina (Upper-Middle Income Country, MIC) | 1,013 ICU admissions (pre: 2015 n=406; post: 2016-17 n=607) | Weekly 45-min case-based tele-education rounds using CERTAIN checklist (US intensivists as preceptors via video, structured patient presentations/discussions) | Pre-tele-education (usual care) vs post-intervention (structured tele-rounds with evidence-based prompts). | Tele-education reduced ICU mortality (43%→27%), hospital mortality (51%→44%), ICU LOS (8.3→3.6 days), costs ($400K savings over 2 years); improved processes (DVT prophylaxis, sedation assessment); high staff satisfaction; feasible in resource-limited MICU via train-the-trainer. | 5,6 |
| Addison JD, 2024. (36) | Multicenter retrospective cohort study | USA (High-Income Country, HIC) | 109 mechanically ventilated ARDS patients requiring continuous neuromuscular blockade | Propofol continuous infusion for deep sedation | Midazolam continuous infusion for deep sedation (both with neuromuscular blocking agents | No difference in time to ventilator liberation (propofol 121h vs midazolam 98h, p=0.72); propofol associated with faster sedation emergence post-NMBA discontinuation (12.9h vs 31.5h, p<0.01); similar ICU/hospital LOS, mortality, adverse events; propofol viable alternative to midazolam in ARDS with NMBA | 5,6 |
| Choi HR, 2022., (37) | Narrative review | South Korea (High-Income Country, HIC) | N/A (review of remimazolam trials and pharmacology) | Remimazolam continuous infusion for ICU sedation | Midazolam, propofol, dexmedetomidine (pharmacokinetics, efficacy, safety profiles) | Remimazolam (ultra-short-acting benzodiazepine) offers rapid onset/offset via esterase metabolism (independent of liver/kidney function); comparable sedation depth to propofol/midazolam with less hypotension/respiratory depression; promising for procedural sedation and ICU use (phase II trials successful); requires dosing studies for prolonged infusions in ARDS/sepsis. | 5 |
| Chanques G, 2022., (38) | Expert consensus review/guideline | International (High-Income Countries, HIC) | N/A (guideline; synthesizes ARDS sedation trials) | Analgesia-first (opioids: fentanyl/remifentanil), tiered sedation (mild RASS -1/+1 → moderate -2/-3 → deep -4/-5), short-acting agents (propofol, dexmedetomidine), NMBA (cisatracurium) for severe ARDS as rescue. | Deep continuous sedation vs minimal sedation; opioids vs sedatives first; NMBA vs no NMBA (ACURASYS vs ROSE trials). | ARDS requires tailored analgesia/sedation beyond minimal strategies; prioritize ventilator optimization/asynchrony control before deepening; analgesia-first reduces sedative needs; deep sedation/NMBA justified short-term in severe cases but daily interruption essential; monitor RASS/pain/delirium; no survival benefit from routine deep sedation. | 5,6 |
| Rasulo FA, 2022. (39) | Prospective cohort comparison study | Italy/Europe (High-Income Country, HIC) | 101 critically ill ARDS patients (COVID-19 vs non-COVID-19 cohorts) | N/A (observational assessment of sedation depth via processed EEG and CAM-ICU). | Excessive sedation (deep sedation beyond target) in COVID-19 ARDS vs non-COVID-19 ARDS patients. | Excessive sedation independently associated with delirium (OR 10.59, p=0.001), longer ICU-LOS (β=10.24 days, p=0.002), and higher ICU mortality (OR 6.78, p=0.009) specifically in COVID-19 ARDS (72.3% oversedated); no significant association in non-COVID cohort; recommends continuous EEG monitoring in COVID-19 patients | 5,6 |
| Shah FA, 2017. (40), | Narrative review | USA (High-Income Country, HIC) | N/A (review of ARDS sedation trials) | Light sedation protocols (non-benzodiazepine regimens: propofol/dexmedetomidine), daily interruptions, protocol-directed titration to RASS/SAS targets. | Deep sedation vs light/minimal sedation; usual care vs protocolized approaches (e.g., SEDCOM, ABC trials) | Deep sedation linked to prolonged mechanical ventilation, ICU-LOS, and mortality in ARDS; light sedation strategies reduce ventilator days, LOS, and tracheostomy needs without increasing adverse neuropsychological outcomes; prioritize analgesia-first, avoid benzodiazepines; ongoing trials test light sedation vs early NMBA in severe ARDS. | 5,6 |
| Fu PK, 2021. (41), | Retrospective cohort study | Taiwan (High-Income Country, HIC) | 109 ARDS patients receiving prone position therapy | High-ratio enteral nutrition (EN ≥70% of total caloric intake) during prone positioning. | High EN ratio vs low EN ratio (<70%); total caloric intake stratified by EN proportion. | High EN ratio associated with lower hospital mortality (adjusted OR 0.25, 95% CI 0.08-0.74, p=0.013); adequate caloric intake (≥25 kcal/kg/day) with high EN further reduced mortality; no increase in complications like aspiration or feeding intolerance | 7 |
| Torres JA, 2023. (42), | Retrospective post-hoc analysis of EDEN trial | USA (High-Income Country, HIC) | 1,000 ARDS patients from EDEN RCT (trophic vs full enteral feeding arms) | Early full enteral calories (>3000 kcal in first 6 days of MV) vs lower caloric intake | High early calories subgroup vs low/medium; original trophic (400 kcal/d) vs full feeding (1300 kcal/d) from EDEN. | >3000 kcal within first 6 MV days associated with lower mortality (OR 0.52, p<0.05) in ARDS; challenges EDEN's null primary result by identifying high-calorie responders; no ventilator-free days benefit but suggests caloric thresholds matter beyond trophic/full dichotomy. | 7 |
| Singer P., 2023. (43), | Practical guideline (partially revised ESPEN ICU nutrition guideline). | Europe/International (High-Income Countries, HIC) | N/A (guideline synthesizing ICU nutrition RCTs and meta-analyses) | Early nutrition (within 48h), hypocaloric feeding (≤70% energy needs week 1), high protein (1.3 g/kg/day), enteral preferred over parenteral, progressive EN advancement. | Early vs delayed nutrition; hypocaloric vs isocaloric; EN vs PN; permissive underfeeding vs full feeding (e.g., EPaNIC, CALORIES trials) | All ICU patients >48h at malnutrition risk; oral/EN first if feasible; hypocaloric early (20-70% needs) over full calories to avoid harm; protein targets 1.3g/kg regardless of energy; monitor NRS2002≥5 for high-risk; supplement glutamine/omega-3 conditionally; special considerations for obesity/ARDS (early trophic EN) | 7 |
| Schenck EJ, (44), 2019. | Secondary analysis of pooled individual patient data from six RCTs (ARDSNet trials) | USA (High-Income Country, HIC) | 4,361 adults with ARDS enrolled in ARMA, ALVEOLI, FACTT, ALTA, EDEN, SAILS | Various trial interventions (e.g., low vs traditional tidal volume, conservative vs liberal fluid, high vs low PEEP, trophic vs full EN, rosuvastatin vs placebo); focus of this paper is not a new intervention but classification of rapidly improving ARDS (riARDS). | riARDS vs non‑riARDS. riARDS defined as patients extubated or no longer meeting Berlin oxygenation criteria within 1 day after randomization | Future ARDS therapeutic trials should consider excluding or stratifying riARDS patients to improve power and better target higher‑risk populations. | 3,4,5 |
| Roncon-Albuquerque R Jr, 2023., (45), | Prospective multicenter observational cohort study. | Portugal, two tertiary ECMO centers (High-Income Country, HIC) | 254 adults with severe ARDS supported with venovenous ECMO over 6 years; 54 (21%) were extubated during ECMO. | Standardized daily protocol for assessing readiness for extubation while still on ECMO, based on adapted ELSO criteria: cooperative patient without high risk of cannula dislodgement, manageable secretions, no airway obstruction, and predefined gas exchange thresholds (e.g., PaO₂/FiO₂ ≥150 on PEEP ≤10 cmH₂O). High-flow nasal cannula was initiated immediately after extubation, with ECMO sweep gas titrated to control work of breathing and gas exchange. | EXT group: patients extubated during ECMO.  CTRL group: patients kept intubated until after ECMO weaning, following a conventional “ECMO first, extubation later” strategy. | A structured “awake-ECMO” extubation protocol can safely identify a subset of severe ARDS patients who benefit from earlier liberation from invasive ventilation, with shorter ECMO/ICU courses and improved survival, but requires careful selection and vigilant thrombosis prevention and airway/cannula management. | 1,4 |
| Patel D., 2024., (46) | Retrospective single‑center cohort study. | USA, large academic tertiary care center (High‑Income Country, HIC) | 98 adults with COVID‑19 who underwent tracheostomy during the first pandemic year (out of 406 mechanically ventilated COVID‑19 patients, ≈24%) | Surgical and percutaneous tracheostomies performed under a standardized institutional COVID protocol; patients stratified by timing of tracheostomy (early ≤14 days vs late >14 days from intubation) | Early vs late tracheostomy timing.  Tracheostomy cohort vs contemporaneous ventilated COVID‑19 patients without tracheostomy for some outcomes. | In a high‑volume tertiary center, protocolized early tracheostomy for selected COVID‑19 patients requiring prolonged ventilation appears safe and is associated with shorter ventilation and ICU/hospital courses, without increasing mortality. | 1,4 |
| Mohsen S., 2022., (47), | Retrospective multicenter cohort study | Canada (medical–surgical ICUs in Alberta; High-Income Country, HIC). | 25,537 unique adult ICU patients; 14,847 had at least one episode of delirium and were included in duration analyses. | None (observational). | Primary outcome: prevalence of delirium (ever vs never delirious), analyzed by admission type and admission GCS.  Secondary outcome: duration of delirium (days with positive ICDSC), comparing physical presence, phone-only, and no contact. | Family engagement—either in person or by phone—is associated with a clinically meaningful reduction in delirium duration in ICU patients, while its impact on delirium incidence depends on admission type and neurologic status at ICU entry. | 1,3,4,5 |
| Paton M, 2024,. (48), | Narrative review | Australia (High-Income Country, HIC) | N/A (review synthesizing ICU rehab trials in ARDS) | Early rehabilitation (active mobilization, exercise, neuromuscular electrical stimulation) during acute ARDS phase, tailored to disease severity (mild-moderate vs severe), ventilator status, and medical therapies (e.g., proning, ECMO, NMBA). | Early vs delayed rehab; usual care vs protocolized mobility; considerations for respiratory compromise limiting exercise tolerance. | Early rehab is best practice in ICU but ARDS respiratory failure often restricts participation; feasible even in moderate-severe cases using low-intensity bedside exercises; improves physical function, muscle strength, and weaning success without excess adverse events; disease severity guides technique selection (e.g., passive for deep sedation, cycling for awake patients); integrates with ARDS management to optimize recovery.  Clinicians should use severity-stratified, safe rehab protocols in ARDS to enhance survivor outcomes, with practical tools for patient identification and progression despite respiratory limitations. | 1,5, |
| Koff MD, 2011., (49) | Prospective before-after intervention study | USA (High-Income Country, HIC) | 3,754 ventilator days (1,993 pre-intervention, 1,761 post-intervention) in a mixed medical-surgical ICU. | Novel hand hygiene program including alcohol-based hand rub dispensers at each bed, visual reminders, staff education, and compliance monitoring with immediate feedback. | Pre-intervention (standard hand hygiene) vs post-intervention (enhanced program) periods. | Targeted, multifaceted hand hygiene interventions can substantially reduce VAP rates in mixed ICUs through sustained compliance improvements. | 8 |
| Han J., 2010. , (50) | Systematic review and meta-analysis. | International (High-Income Countries, HIC) | 7 RCTs with 1,606 mechanically ventilated patients. | No routine ventilator circuit changes vs routine changes (daily, every 2 days, every 3-7 days). | Frequent/routine circuit changes vs infrequent/no routine changes on VAP incidence. | Routine ventilator circuit changes are unnecessary and potentially harmful; change only when visibly contaminated or malfunctioning to avoid VAP risk from handling. | 8 |

# References

1. Ma W, Tang S, Yao P, et al. Advances in acute respiratory distress syndrome: focusing on heterogeneity, pathophysiology, and therapeutic strategies. Signal Transduct Target Ther. 2025;10:75.
2. Ferguson ND, Fan E, Camporota L, et al. The Berlin definition of ARDS: an expanded rationale, justification, and supplementary material. Intensive Care Med. 2012;38:1573-82.
3. Sadana D, Kaur S, Sankaramangalam K, et al. Mortality associated with acute respiratory distress syndrome, 2009-2019: a systematic review and meta-analysis. Crit Care Resusc. 2023;24:341-351.
4. Kovacevic P, Matijasevic J, Dragic S, et al. Characteristics and outcomes of critically ill patients with influenza A (H1N1) in the Western Balkans during the 2019 post-pandemic season. Indian J Med Microbiol. 2020;38:415-420.
5. Čučković M, Drmić Ž, Pražetina M, Tipura D, Ćurčić M, Miko I, Mihelčić A, Romić A, Kukoč A, Blagaj V, Lasić H, Dolenc E, Hleb S, Almahariq H, Šribar A, Peršec J, Lukšić I. Epidemiological characteristics, baseline clinical features, and outcomes of critically ill patients treated in a coronavirus disease 2019 tertiary center in continental Croatia. Croat Med J. 2022 Feb 28;63(1):6-15.
6. Sharma S, Verhagen AP, Elkins M, Brismée JM, Fulk GD, Taradaj J, Steen L, Jette A, Moore A, Stewart A, Hoogenboom BJ, Söderlund A, Harms M, Pinto RZ. Research from low-income and middle-income countries will benefit global health and the physiotherapy profession, but it requires support. J Man Manip Ther. 2023 Oct;31(5):305-310.
7. Kovacevic P. Can the terms "low resource setting" and "low-income country" be used interchangeably in the context of intensive care medicine? Intensive Care Med. 2023;49:1274-1275.
8. Kwizera A, Kabatooro D, Atumanya P, Tumukunde J, Kalungi J, Mwanje AK, Obua D, Agaba P, Sendagire C, Nakibuuka J, Owachi D, Dünser MW, Alenyo-Ngabirano A, Olaro C, Kyobe-Bosa H, Kirenga BJ, Nakiyingi L, Kiwanuka N, Kateete DP, Joloba M, Sewankambo N, Summers C. Respiratory Support Techniques for COVID-19-Related ARDS in a Sub-Saharan African Country: A Multicenter Observational Study. Chest. 2023;164:369-380.
9. Kovacevic P, Meyer FJ, Gajic O. Successful implementation of modern critical care in the low-resources country Bosnia and Herzegovina : Single-center experience. Med Klin Intensivmed Notfmed. 2022;117:269-275.
10. Inglis R, Ayebale E, Schultz MJ. Optimizing respiratory management in resource-limited settings. Curr Opin Crit Care. 2019;25:45-53.
11. Grasselli G, Calfee CS, Camporota L, et al.; European Society of Intensive Care Medicine Taskforce on ARDS. ESICM guidelines on acute respiratory distress syndrome: definition, phenotyping and respiratory support strategies. Intensive Care Med. 2023;49:727-759.
12. Serpa Neto A, Checkley W, Sivakorn C, Hashmi M, Papali A, Schultz MJ; COVID-LMIC Task Force and the Mahidol-Oxford Research Unit (MORU). Pragmatic Recommendations for the Management of Acute Respiratory Failure and Mechanical Ventilation in Patients with COVID-19 in Low- and Middle-Income Countries. Am J Trop Med Hyg. 2021;104:60-71.
13. Duarte PAD, Rea-Neto A. Mortality in mechanically ventilated patients in middle-income countries: a call for action. Lancet Glob Health. 2022;10:e163-e164.
14. Matthay MA, Thompson BT, Ware LB. The Berlin definition of acute respiratory distress syndrome: should patients receiving high-flow nasal oxygen be included? Lancet Respir Med. 2021;9:933-936.
15. Ranieri VM, Rubenfeld G, Slutsky AS. Rethinking Acute Respiratory Distress Syndrome after COVID-19: If a "Better" Definition Is the Answer, What Is the Question? Am J Respir Crit Care Med. 2023;20:255-260.
16. Chebib N, Kreitmann L, Guérin C. Acute respiratory distress syndrome-a worldwide economic perspective. J Thorac Dis. 2018;10:570-575.
17. Vukoja M, Riviello ED, Schultz MJ. Critical care outcomes in resource-limited settings. Curr Opin Crit Care. 2018;24:421-427.
18. Matthay MA, McAuley DF, Ware LB. Clinical trials in acute respiratory distress syndrome: challenges and opportunities. Lancet Respir Med. 2017;5:524-534.
19. Cardiff University**.** How To Conduct a Delphi Study. Cardiff University; 2017. Available from: https://www.cardiff.ac.uk/__data/assets/pdf_file/0010/1164961/how_to_conduct_a_delphistudy.pdf
20. Calibrum. Surveylet: real-time Delphi survey software [Internet]. St. George (UT): Calibrum; 2020 [cited 2025 Mar 13]. Available from: [https://calibrum.com](https://calibrum.com?utm_source=chatgpt.com)
21. ARDS Definition Task Force; Ranieri VM, Rubenfeld GD, Thompson BT, Ferguson ND, Caldwell E, Fan E, Camporota L, Slutsky AS. Acute respiratory distress syndrome: the Berlin Definition. JAMA. 2012;307:2526-33.
22. Gattrell WT, Logullo P, van Zuuren EJ, Price A, Hughes EL, Blazey P, Winchester CC, Tovey D, Goldman K, Hungin AP, Harrison N. ACCORD (ACcurate COnsensus Reporting Document): A reporting guideline for consensus methods in biomedicine developed via a modified Delphi. PLoS Med. 2024 Jan 23;21(1):e1004326.
23. Gawda R, Piwoda M, Marszalski M et al. Establishing a New ECMO Referral Center Using an ICU-Based Approach: A Feasibility and Safety Study. Healthcare (Basel). 2022;10:414.
24. Losonczy LI, Papali A, Kivlehan S et al. White Paper on Early Critical Care Services in Low Resource Settings. Ann Glob Health. 2021;87:105.
25. Kovacevic P, Vidovic J, Tomic B, Mallat J, Hssain AA, Rotimi M, Akindele OT, Doi K, Mishra R, Meyer FJ, Palibrk I, Skrbic R, Boloña E, Kilickaya O, Gajic O. Consensus statements for the establishment of medical intensive care in low-resource settings: international study using modified Delphi methodology. Crit Care. 2024;28:323.
26. Carvalho EB, Leite TRS, Sacramento RFM, et al. Rationale and limitations of the SpO2/FiO2 as a possible substitute for PaO2/FiO2 in different preclinical and clinical scenarios. Rev Bras Ter Intensiva. 2022;34:185-196.
27. Erlebach R, Pale U, Beck T, et al. Limitations of SpO_2_ / FiO_2_-ratio for classification and monitoring of acute respiratory distress syndrome-an observational cohort study. Crit Care. 2025;29:82.
28. Wick KD, Matthay MA, Ware LB. Pulse oximetry for the diagnosis and management of acute respiratory distress syndrome. Lancet Respir Med. 2022;10:1086-1098.
29. Matthay MA, Arabi Y, Arroliga AC, et al. A New Global Definition of Acute Respiratory Distress Syndrome. Am J Respir Crit Care Med. 2024;209:37-47.
30. Bass CM, Sajed DR, Adedipe AA, West TE. Pulmonary ultrasound and pulse oximetry versus chest radiography and arterial blood gas analysis for the diagnosis of acute respiratory distress syndrome: a pilot study. Crit Care. 2015;19:282.
31. Smit MR, Mayo PH, Mongodi S. Lung ultrasound for diagnosis and management of ARDS. Intensive Care Med. 2024;50:1143-1145.
32. Plantinga C, Klompmaker P, Haaksma ME, et al. Use of Lung Ultrasound in the New Definitions of Acute Respiratory Distress Syndrome Increases the Occurrence Rate of Acute Respiratory Distress Syndrome. Crit Care Med. 2024;52:e100-e104.
33. Riviello ED, Kiviri W, Twagirumugabe T, Mueller A, Banner-Goodspeed VM, Officer L, Novack V, Mutumwinka M, Talmor DS, Fowler RA. Hospital Incidence and Outcomes of the Acute Respiratory Distress Syndrome Using the Kigali Modification of the Berlin Definition. Am J Respir Crit Care Med. 2016;193:52-9.
34. Vukoja M, Dong Y, Adhikari NKJ, et al. CERTAIN Investigators of the SCCM Discovery Network. Checklist for Early Recognition and Treatment of Acute Illness and Injury: An Exploratory Multicenter International Quality-Improvement Study in the ICUs With Variable Resources. *Crit Care Med*. 2021;49:e598–e612.
35. Kovačević P, Dragić S, Kovačević T, et al. Impact of weekly case-based tele-education rounds on quality of care in a resource-limited medical intensive care unit. *Crit Care*. 2019;23:220.
36. Addison JD, Daley MJ, Curran M, Hodge EK. A Comparison of Midazolam and Propofol for Deep Sedation in Patients with Acute Respiratory Distress Syndrome Requiring Neuromuscular Blocking Agents. J Pharm Pract. 2024;37:271-278.
37. Choi HR, Song IA. Review of remimazolam and sedatives in the intensive care unit. Acute Crit Care. 2022;37:151-158.
38. Chanques G, Constantin JM, Devlin JW, et al. Analgesia and sedation in patients with ARDS. Intensive Care Med. 2020;46:2342-2356.
39. Rasulo FA, Badenes R, Longhitano Y, et al. Excessive Sedation as a Risk Factor for Delirium: A Comparison between Two Cohorts of ARDS Critically Ill Patients with and without COVID-19. Life (Basel). 2022;12:2031.
40. Shah FA, Girard TD, Yende S. Limiting sedation for patients with acute respiratory distress syndrome - time to wake up. Curr Opin Crit Care. 2017;23:45-51.
41. Fu PK, Chao WC, Hsu CY, Wang CH, Wang CY. Caloric Intake with High Ratio of Enteral Nutrition Associated with Lower Hospital Mortality for Patients with Acute Respiratory Distress Syndrome Using Prone Position Therapy. Nutrients. 2021;13:3259.
42. Torres JA, Pak Y, Tayek JA. Does early enteral calories in ARDS improve outcome? A retrospective evaluation of the EDEN trial. Clin Nutr Open Sci. 2023;49:86–92.
43. Singer P, Blaser AR, Berger MM, et al. ESPEN practical and partially revised guideline: Clinical nutrition in the intensive care unit. Clin Nutr. 2023;42:1671-1689.
44. Schenck EJ, Oromendia C, Berlin DA, Choi AMK, Siempos II. Rapidly improving ARDS in therapeutic randomized trials. *Chest*. 2019;155:474-482.
45. Roncon-Albuquerque R Jr, Bastos J, Rocha C, et al. Standardized approach for extubation during extracorporeal membrane oxygenation. *Ann Intensive Care*. 2023;13:93.
46. Patel D, McGuire SP, Menon J, et al. The COVID-19 tracheostomy experience at a large tertiary care center. *J Clin Med*. 2024;13:2130.
47. Mohsen S, Moss SJ, Lucini F, et al. Impact of Family Presence on Delirium in Critically Ill Patients: A Retrospective Cohort Study. Crit Care Med. 2022;50:1628-1637.
48. Paton M, Hodgson CL. Early Rehabilitation in Acute Respiratory Distress Syndrome. Clin Chest Med. 2024;45:895-904.
49. Koff MD, Corwin HL, Beach ML, Surgenor SD, Loftus RW. Reduction in ventilator associated pneumonia in a mixed intensive care unit after initiation of a novel hand hygiene program. J Crit Care. 2011;26:489-495.
50. Han J, Liu Y. Effect of ventilator circuit changes on ventilator-associated pneumonia: a systematic review and meta-analysis. Respir Care. 2010;55:467-74.
